# Proteomic data and structure analysis combined reveal interplay of structural rigidity and flexibility on selectivity of cysteine cathepsins

**DOI:** 10.1038/s42003-023-04772-8

**Published:** 2023-04-24

**Authors:** Livija Tušar, Jure Loboda, Francis Impens, Piotr Sosnowski, Emmy Van Quickelberghe, Robert Vidmar, Hans Demol, Koen Sedeyn, Xavier Saelens, Matej Vizovišek, Marko Mihelič, Marko Fonović, Jaka Horvat, Gregor Kosec, Boris Turk, Kris Gevaert, Dušan Turk

**Affiliations:** 1 Jožef Stefan Institute, Department of Biochemistry and Molecular and Structural Biology, Jamova cesta 39, 1000 Ljubljana, Slovenia; 2 Centre of Excellence for Integrated Approaches in Chemistry and Biology of Proteins (CIPKeBiP), Jamova cesta 39, 1000 Ljubljana, Slovenia; 3 The Jožef Stefan International Postgraduate School, Jamova cesta 39, 1000 Ljubljana, Slovenia; 4 VIB-UGent Center for Medical Biotechnology and UGent Department of Biomolecular Medicine, Technologiepark-Zwijnaarde 75, 9052 Ghent, Belgium; 5 VIB-UGent Center for Medical Biotechnology and, Department for Biochemistry and Microbiology, Ghent University, 9052 Ghent, Belgium; 6 Acies Bio d.o.o., Tehnološki park 21, 1000 Ljubljana, Slovenia; 7 Faculty of Chemistry, University of Ljubljana, Večna pot 113, SI-1000 Ljubljana, Slovenia

**Keywords:** Proteases, Drug discovery

## Abstract

Addressing the elusive specificity of cysteine cathepsins, which in contrast to caspases and trypsin-like proteases lack strict specificity determining P1 pocket, calls for innovative approaches. Proteomic analysis of cell lysates with human cathepsins K, V, B, L, S, and F identified 30,000 cleavage sites, which we analyzed by software platform SAPS-ESI (Statistical Approach to Peptidyl Substrate-Enzyme Specific Interactions). SAPS-ESI is used to generate clusters and training sets for support vector machine learning. Cleavage site predictions on the SARS-CoV-2 S protein, confirmed experimentally, expose the most probable first cut under physiological conditions and suggested furin-like behavior of cathepsins. Crystal structure analysis of representative peptides in complex with cathepsin V reveals rigid and flexible sites consistent with analysis of proteomics data by SAPS-ESI that correspond to positions with heterogeneous and homogeneous distribution of residues. Thereby support for design of selective cleavable linkers of drug conjugates and drug discovery studies is provided.

## Abstract (web summary)

Proteomics analysis, machine learning, and crystal structures reveal the substrate specificity of cysteine cathepsins.

## Introduction

In drug discovery projects it is crucial to allocate and tap the potential structural area for binding of drug candidates. Fragment screening is a structure-driven approach which assesses the potential of patches on a target protein surface for binding of functional groups^1^. Massive peptide screening offers an alternative that may be particularly beneficial for targeting enzymes that modify proteins such as proteases. Cysteine cathepsins appear particularly suitable for demonstrating the potential of such an approach because of their lack of a specificity pocket^2^, which results in elusive specificity profiles, making prediction of their cleavage sites in substrates far from obvious^3^. Moreover, cysteine cathepsins are relevant drug targets given their specific physiological roles, such as cleavage of collagen by cathepsin K during bone remodeling^4,5^ and cleavage of Ii during MHC class II maturation by cathepsins S and V^6^, and pathological roles in diseases such as tumor progression^3,5^ and activation of viruses such as corona and Ebola^7–11^.

The quest for specific protein sequences cleaved by proteases dates back to the origins of their biochemistry from the first established assays onward including the pioneering work of Schechter and Berger who made the first attempt to match positions of substrate residues and their subsites on the protein surface using papain as an example^12^. Positional preference for amino acid residues was established by displaying the distribution of residues in the form of sequence logos, which were initially developed for nucleic acids by Schneider^13^ and later enhanced in the advanced iceLogo application^14,15^. A number of screens and approaches have been applied, each biased in its own way either due to sequence limitations or presentation and analysis of data^16–20^. Moreover, such studies focused on the specificity of substrate positions, whereas a view integrating data from substrate cleavages and their interactions with the underlying protease surface made visible by structure determination is lacking.

## Results

**Fig. 1:**
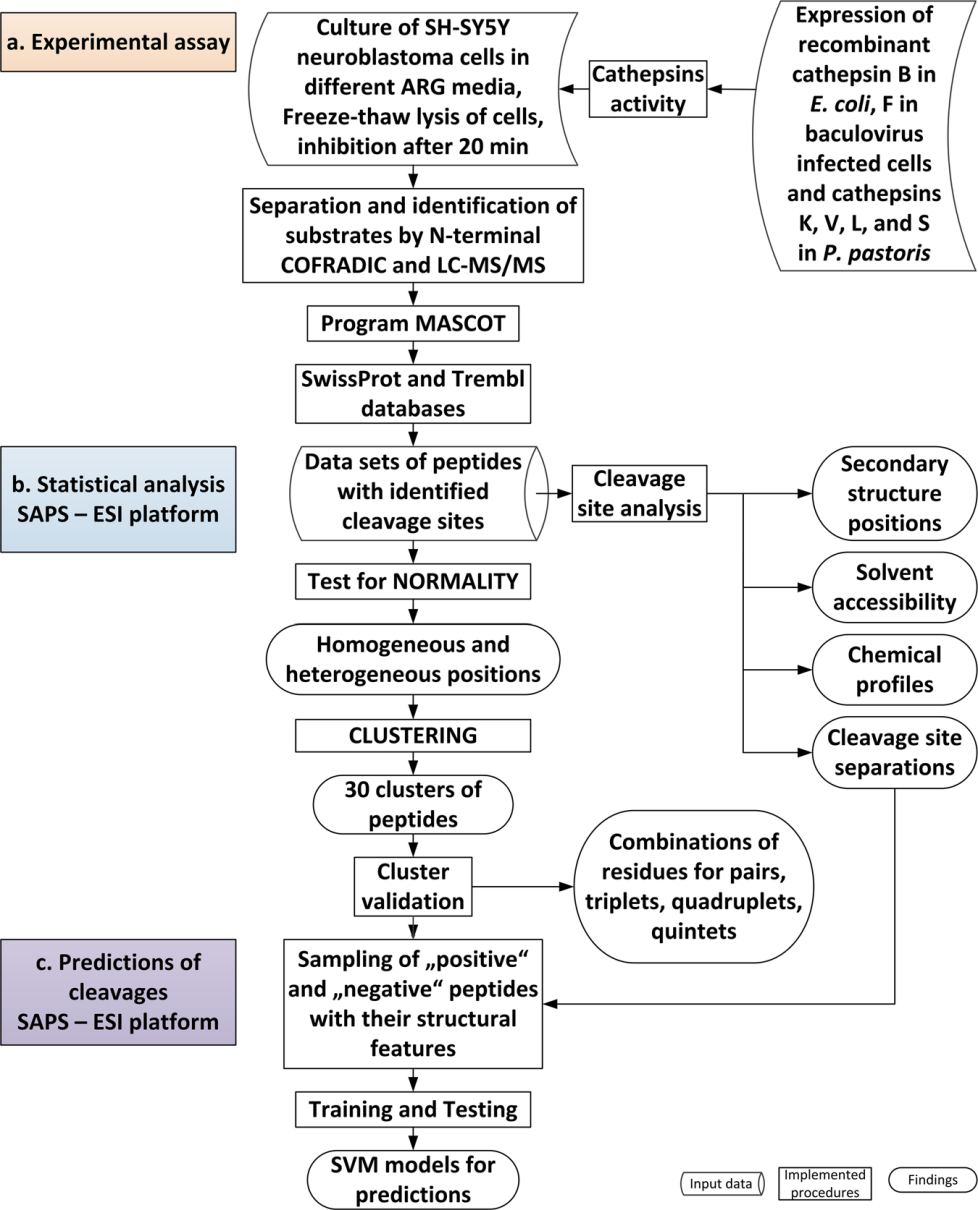
Flowchart of the procedure. The procedure consisted of three steps: **a** Experimental assay, **b** SAPS-ESI statistical analysis, and **c** SAPS-ESI predictions, which are highlighted in **a** orange, **b** blue and **c** violet. SAPS-ESI platform was used in combination with MAIN program^59^ and PCSS server^16^.

In the experimental part of our study (Fig. 1a), human neuroblastoma SH-SY5Y cell lysates were treated separately with recombinant human cathepsins K, V, L, S, F, and B. Cleavage sites from the endoproteolytic activity of these cathepsins were determined by N-terminal combined fractional diagonal chromatography (COFRADIC)^21^. To ensure that endogenous proteolytic activity was excluded we used metabolic labelling (SILAC-based) and thus always a control condition (a cell lysate not incubated with a cathepsin) to compare against^22^. The data were analyzed by our software platform SAPS-ESI (Statistical Approach to Peptidyl Substrate-Enzyme Specific Interactions), generated for this purpose (Fig. 1b, c). The platform SAPS-ESI consisted of methods described in details in Methods. Briefly, the platform contains two parts: statistical analysis and prediction of cleavages. Statistical analysis part assigns cleavage sites structural features such as secondary structure and solvent exposure, and establishes chemical profiles of residues, and separation between cleavage sites on the same protein substrate. Next normality of amino acid residue distribution for each position is assessed independently. The positions with non-normal distributions, called heterogeneous, then enter the clustering. In the prediction of cleavages, the assigned structural features, distribution of amino acid residues, chemical profiles, cluster assignments, and distance from the cleavage site are combined to generate positive and negative sequences of peptides for training the SVM models. The success of predictions platform was later experimentally validated.

The total number of analyzed cleavage sites was 29,674. Each among cathepsins K, V, B, L, S, and F contributed from 9583 to 3500 cleavage sites, which belonged to 3167 different proteins (Supplementary Data 1 and Supplementary Table 1). Among them 1592 had at least partial structures deposited in protein structure database^23^. In the case of multiple entries of the same protein, only the entry with the maximum sequence coverage and highest resolution were included. Many cleavages occurred close to each other (Supplementary Fig. 1a). Separation of cleavages was first analyzed for each cathepsin independently and later also in combinations of two (Supplementary Fig. 1b, c). All cathepsins have most cleavages occurring one residue apart, however, drop in the number of cleavages at longer separations is more gradual for those of cathepsins L and V, than those of cathepsins K, B, S, and F, which exhibit sudden drop of the cleavages after one residue separation (Supplementary Fig. 1b). Due to the prevailing number of the cleavages one residue apart it is unlikely that they originated from the exopeptidase activity of cathepsins, even though such activity cannot be completely ruled out. This view is supported by the procathepsin B activation study^24^ which showed that mutations of residues to proline shifted the cleavage site to adjacent positions. The majority of cleavage site residues were surface accessible (total 94%) and 43% of cleavages lied outside elements of secondary structures (Supplementary Fig. 1d, e). The view combining cleavages of all cathepsins enabled us to discriminate between shared, unique and single cleavages of the studied cathepsins (Supplementary Data 1). Shared cleavages are those generated by more than one cathepsin cleaving the same site of the same protein (19,088 or 64% of cleavages) (Supplementary Table 1). Unique cleavages are those performed by only one of the cathepsins (10,586 or 36% of the cleavages) on a protein that was cleaved at more than one site by one or more cathepsins, and single cleavages are those performed by a single cathepsin on a single site in a given protein (941 or 3% of the cleavages) (Supplementary Data 2). We believe that at least some of these single cleavages hint to biological roles that may facilitate further studies (Supplementary Data 2). Supplementary Fig. 2 shows heat shock cognate 71 kDa protein with 21 shared (among them are 2 cleavages for all six cathepsins colored dark red) and 18 unique cleavages colored pink (PDB code 3LDQ; Macias et al. 2011^25^), whereas the Supplementary Fig. 3 contains three examples of single cleavages.

### Heterogeneous and homogeneous positions of peptidyl substrates

**Fig. 2:**
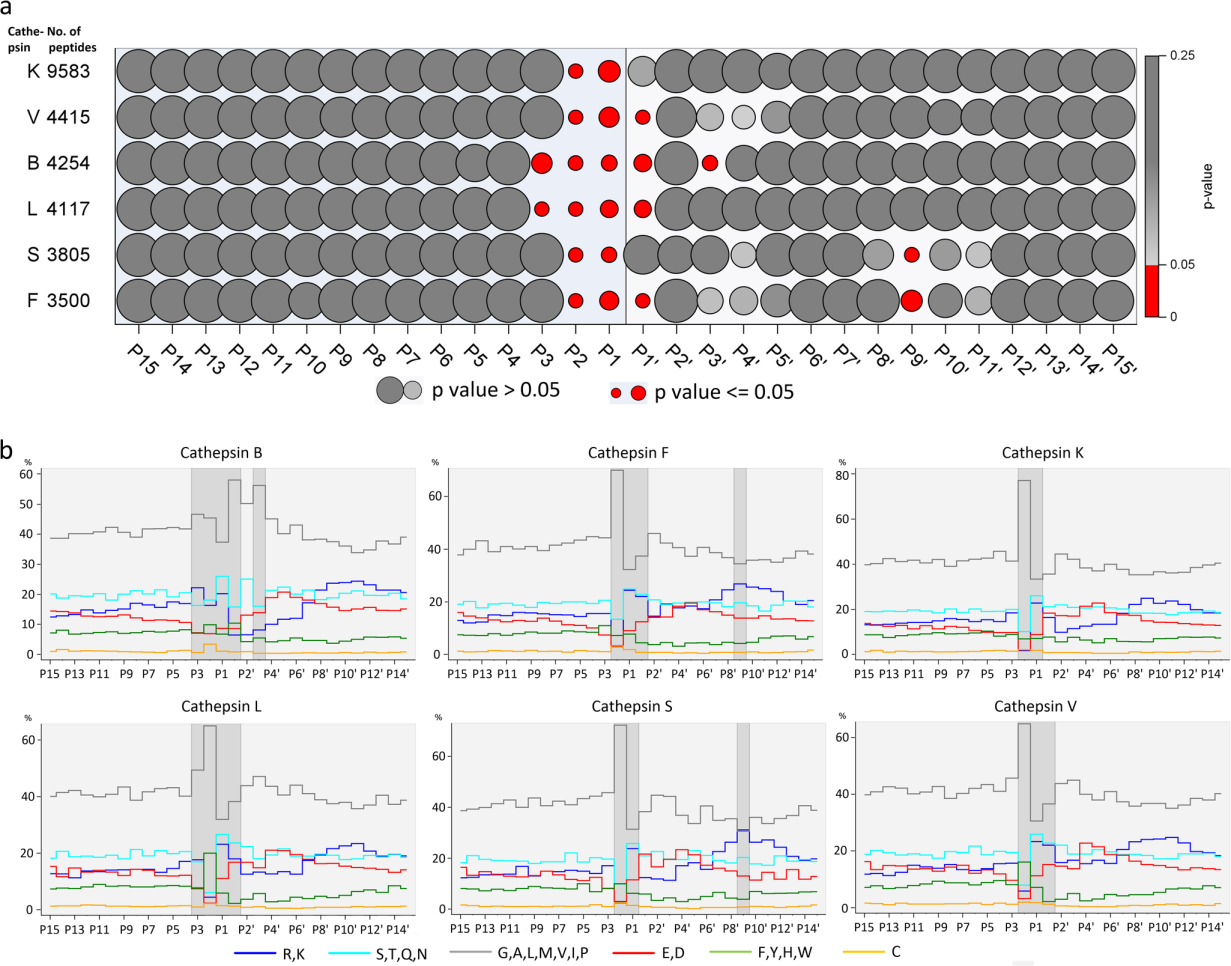
Heterogeneous positions indicate specificity positions. **a** Normality of distributions. *P*-values for normality of distributions of residues at positions from P15 to P15′ (columns) for each cathepsin (rows) are indicated by the size of circles. The red and grey circles present p-values equal to or less than 0.05 (non-normal), larger than 0.05 and equal to 1.00 (normal), respectively. **b** Profiles of amino acid residue types. The amino acid residues were classified into 6 groups with share of each presented by a colored step chart: positively charged LYS, ARG (blue), neutral hydrophilic SER, THR, GLN, ASN (cyan), hydrophobic GLY, ALA, LEU, MET, VAL, ILE, PRO (light grey), negatively charged ASP, GLU (red), aromatic PHE, HIS, TYR, TRP (green), and CYS (yellow). The positions in the non-normal distributions of amino acid residues are shaded.

To identify important residue positions contributing to a protease’s specificity, we applied the Anderson–Darling test for normality (Gaussian distribution) of the appearance of residues in a 30-amino acids wide window (from P15 to P15′, according to Schechter and Berger nomenclature^12^). As expected, the appearance of amino acid residues distant from the cleaved sites was consistent with the normal distribution with probability values (*p*-values) above 0.05. We refer to these positions as homogeneous and represent them as large and intermediate gray circles in Fig. 2a. In contrast, several positions near the cleaved sites did not have normal distribution of residues. We refer to these positions as heterogeneous and represented them with small red circles. Interestingly, the range of heterogeneous positions of cathepsins varied.

To provide an insight in heterogeneous positions based on chemical understanding we generated a plot of amino acid residues grouped according to their types (negatively and positively charged, hydrophilic, hydrophobic, neutral, aromatic) (Fig. 2b). The plots showed that the hydrophobic types exhibited the largest deviations at heterogeneous positions and were followed by negatively charged, positively charged, and hydrophilic types. The heterogeneous position P9′ of cathepsins S and F originates from a maxima of positively charged residues as seen from their plots (Fig. 2b). However, due to the distance to the cleavage site they were not included in further analysis. For comparison, Supplementary Fig. 4a shows the distribution of amino acid residues with iceLogo plots^14^.

### From clusters to SVM models

**Fig. 3:**
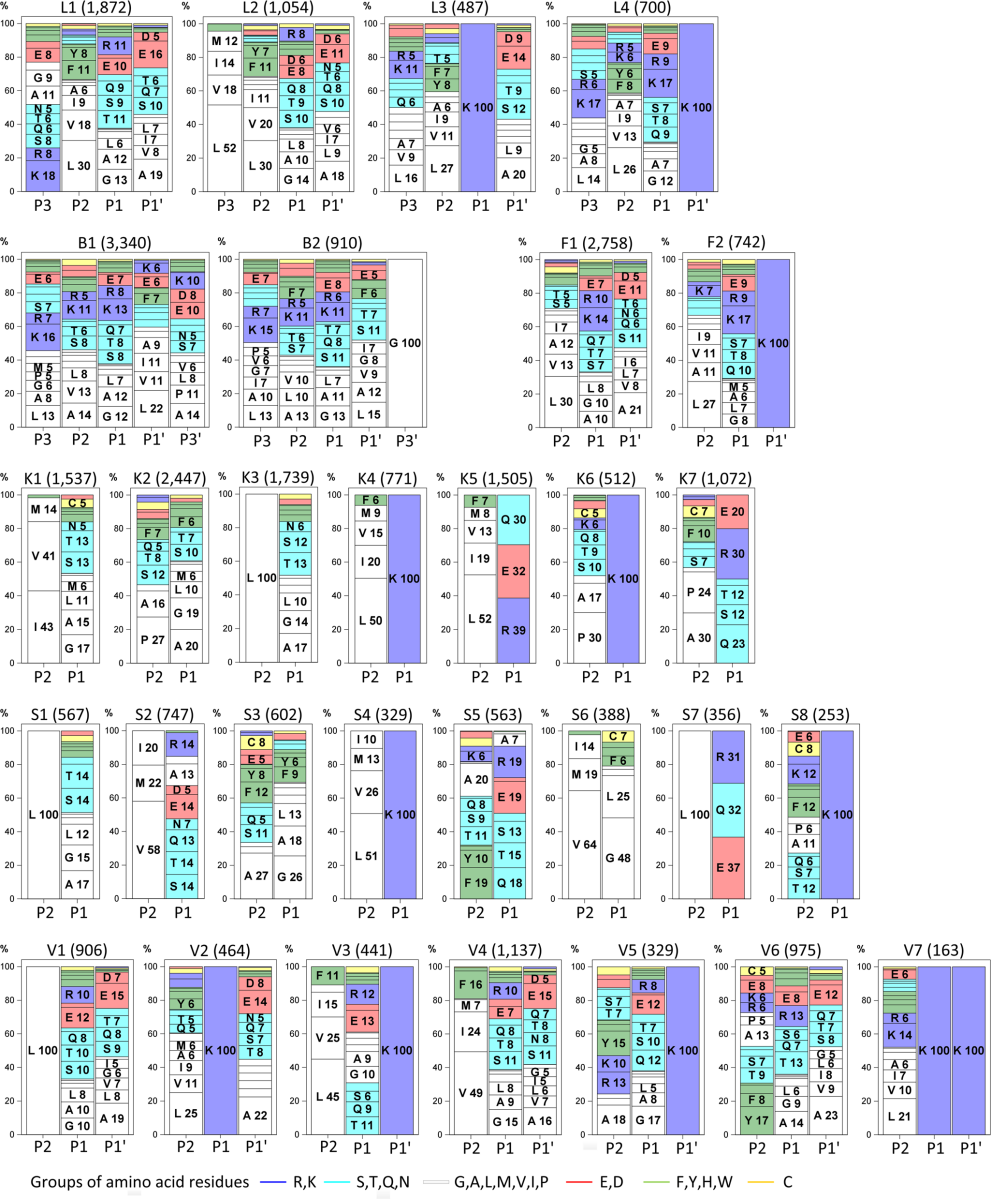
Relevant clusters of substrate specificity. Each frame describes one cluster. The cathepsin identifier, cluster number and the number of the peptides in a cluster are indicated on top of each figure. The blocks of residues are sorted according to their share and the background color represents the chemical type of the amino acid residue.

To gain insight into the diversity of the cleavage sites, the sequences of the heterogeneous substrate regions were clustered using Ward’s minimum variance method. Cubic clustering criterion (CCC) was used for the determination of the number of clusters. By the rule of thumb, CCC values larger than 2.0 indicated defined number of clusters, CCC values between 0.0 and 2.0 corresponded to less defined number of clusters, and negative CCC values corresponded to possible outliers. During optimization of clustering, we optimized distance calculation by using various scoring matrices (BLOSUM62^26^ turned out best), normalization of distances, and positions of amino acids included (heterogeneous positions gave the best outcome). After the first branching, thirty clusters were obtained with CCC values 11.8, 6.69, 12.0, 9.7, 11.6, and 4.51, for cathepsins K, V, B, L, S, and F, respectively. Besides CCC we included another cluster optimization criterion, which we called well-defined cluster. A cluster is well-defined, when it has at least one position with a recognizable pattern. For clustering to work well, at least half of clusters should be well-defined. In our case, 22 out of 30 clusters were well defined: cathepsin S has eight, followed by cathepsins K and V with seven, cathepsin L with four and cathepsins B and F with two. They had at least one heterogeneous position that was filled with one prevailing residue or several residues from the same residue type. Hydrophobic residues (shown in white) predominated throughout the clusters, especially at P2. There were four clusters with dominating preferences for leucine at one position and seven clusters with the prevailing combination of hydrophobic and aromatic residues. Eleven clusters had prevailing lysine at positions P1 or P1′ (shown in blue). Four clusters had two heterogeneous positions occupied either as pair of lysins or hydrophobic positions or their combination (Fig. 3). The preference for glycine at P3′ as the dominant residue of the B2 cluster was previously observed^17^ as well as the cathepsin K preference for proline at P2 present in clusters K2, K6 and K7, which is crucial for its collagenolytic properties^27^. The clusters with a dominating pair of basic residues at P1 and P1′ of cathepsins L and V (L4, V3, V5, and V7) correspond to their cleavage sites in brain pro-hormone processing^28^. For comparison the Supplementary Fig. 4b, c, d, e shows the distribution of amino acid residues of clusters with iceLogo plots^14^.

### SVM models

For the prediction of cleavage sites, we used the publicly available PCSS server (https://modbase.compbio.ucsf.edu/peptide//)^16^. We found the approach of Barkan et al.^16^ most suitable for our needs as it allows remote input of user-provided cleavage site data and matches this input with sequence and structural information and model cleavage site predictions using the support vector machine (SVM) method^29^. The best resolution of SVM models were obtained, when only the heterogeneous positions of substrate sequences were used in clustering. The final training sets contained optimized shares of clustered cleaved peptides (positive peptides) and a considerable number of most likely uncleaved peptides (negative peptides), which were at least 30 residues away from any identified cleavage site (Supplementary Table 2a). The achieved sensitivity or true positive rate (TPR) was in the range of 0.802 to 0.848, and the accuracy ranged from 80 to 91% (Supplementary Data 3, Supplementary Fig. 5). This is comparable with predictions in the literature that include proteases with highly specific P1 residues such as caspases (https://prosper.erc.monash.edu.au/help.html)^18,30^.

### Prediction of cathepsin cleavages of viral proteins

**Fig. 4:**
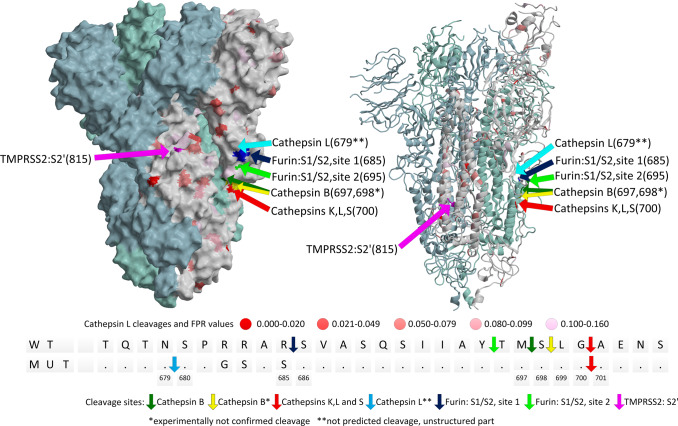
Cleavages of SARS-CoV-2 wild-type (WT) and mutated furin cleavage site (MUT) S protein (6vsb, Wrapp et al. 2020^61^). S protein is trimer, cleavages are presented in the single protomer colored white (chain A). Chain B and C are colored light blue and light green, respectively. The S protein was generated with MAIN^59^ and rendered with RASTER 3D^66^.

Cleavages of SARS-CoV-2 S protein by cathepsins were studied before^31,32^, yet physiologically relevant processing site is most likely only the first cut performed under physiological conditions. To validate our prediction model and propose a possible role for cathepsins, we predicted cleavage sites in the S proteins of the SARS-CoV-2, SARS-CoV and MERS-CoV viruses (Supplementary Table 2b). SVM models predicted cleavages in a large area in the SARS-CoV-2 S protein with the G700-A701 as the most likely cleavage site for cathepsins K, V, L, S and F. To validate our predictions experimentally, we treated furin cleavage site mutated (MUT) and wild-type (WT) S protein with cathepsins B, K, L, S, and V. The bands of S protein fragments on SDS-PAGE (Supplementary Fig. 6) with molecular weights of approximately 55 and 70 kDa were suspected to be the result of the first cathepsin cleavage and were analyzed by N-terminal sequencing (Fig. 4). G700-A701 was indeed identified as the primary cleavage site of cathepsins L (SLG ↑ A is one of most likely sequences in cluster L1), K (LG ↑ is one of the most likely sequences in cluster K3) and S (LG ↑ is one of the most likely sequences in cluster S1), whereas N679-S680 was the secondary cleavage site for cathepsin L, but only in the MUT S protein. The cleavage site for cathepsin V could not be determined due to noisy data, which suggests several neighboring cleavages. Cathepsin B cleaved at the M697-S698 site. These cleavages confirmed our predictions, except for the cathepsin B cleavage, which was one residue away from the neighboring highest ranked predicted cleavage site, S698-L699 (Supplementary Table 2b) and the secondary cathepsin L cleavage in the MUT S protein. The explanation for the failed prediction of the TQTN-SPRR/SPGS (N679-S680) secondary cleavage site on the native/mutated protein is rather simple. It exposes limitations of our training sets. Our data do not contain a matching or similar sequence of such cleavage site, which is consistent with its high FPR value (0.996). Hence, the SVM network did not learn about such cleavage and therefore was not able to predict it. The established cleavage area of cathepsins is vicinal to the furin cleavage site (R685-S686) and distant to the TMPRSS2 cleavage site (R815-S816)^9^. Hence, cathepsins may contribute to the generation of S1 fragments of the tested S protein variants.

### The features of the substrate binding subsites

Mass spectrometry delivers data on the cleaved positions within the substrate sequences, yet these data do not provide insight in interaction of the substrate residues with the features of the protease subsites. Hence, we set out to determine the crystal structures of cathepsin complexes with representative peptides of cleaved substrates. As a working model, cathepsin V was chosen because of the resolution of diffracting crystals (up to 1.3 Å) and crystal packing with accessible active site cleft. 41 different peptide sequences were synthesized: 30 sequences matching cathepsin V cleaved sites were selected among the cleavage sites of all cathepsin V clusters, six of which were also synthesized without termini protection. Four additional sequences that did not match any sequence from protein analysis, as well as the sequence LLKAVAEKQ, were also included in synthesis. The latter was engineered as a hybrid of the LLKVAL and AVAEKQ peptides (Supplementary Table 3). Reactive site mutants C25A and C25S of cathepsin V were used in structural studies. Soaking of cathepsin V crystals and co-crystallization yielded 21 structures of cathepsin V-peptide complexes and 28 unique peptide binding geometries. Of these, 26 bound to the active site in a substrate-like manner and several of them with a different binding geometry in each molecule in the asymmetric unit (Supplementary Table 4). Data collection and refinement statistics are provided in Supplementary Tables 5–15. Not unexpectedly, the cleaved peptides indicated that both cathepsin V mutants retained their residual proteolytic activity under crystallization conditions as observed previously^33,34^.

In addition, we could detect partial processing of some peptides with mutated cathepsin V C25A in solution by detection of peptide fragments with MALDI-TOF but only with prolonged incubation (after approximately 1 day), which is in the scale of cathepsin crystallization and soaking experiments (data now shown).

Peptides exhibited equivalent binding at subsites S2–S2′. At S3, S4, S3′, and S4′, the binding of most peptides still followed the same direction. Beyond S4 and S4′, the electron density maps of most structures worsened, and the noisy maps indicated that there were no clearly defined binding areas. At S1, N and O of the P1 residue formed H-bond to O of D163 and ND of Q19, respectively, and the carboxylic end of cleaved peptides or amide protective groups of protected peptides interacted with NE of H164 (Supplementary Fig. 7). At S2, the carbonyl O of the P2 residue formed an H-bond either to the N of G68 or N of W26, and the N of the P2 residue formed an H-bond with the O of G68. Additionally, the amino group of Lys residues at S2 interacted with the carbonyl O atoms of cathepsin V L162 and peptide P4 residues. At S3, no main-chain interactions occurred, but the side chains of longer residues at P3 interacted with the side chains of Q63 and N66. Two peptide fragments, VACK and TAHE, were the exceptions because their main chain ran into the S3 binding area. At S1′, the carbonyl O of the P1′ residue formed an H-bond with the NE of W190. At S2′, the carbonyl O of the P2′ residue formed an H-bond with the NE of Q145. At S3′, the carbonyl O of the P3′ residue formed an H-bond with the NE atom of Q21. At S4′, N of P4′ formed an H-bond with OE of Q145. The first notable exception in the primed site binding was the peptide RLSAKP with deviation at P4′, where the side chain of Ala was placed instead of H-bonding to Q145, and at P6′, where Pro formed electrostatic interactions with its carboxylic terminal to the amino group of K20. The second exception was the peptide AVAEKQ, which formed an internal H-bond between the O of the P3′ residue and the amino group of P6′, instead of to the side chain amide group of Q21. It also formed additional interactions with neighboring molecules in the crystal.

**Fig. 5:**
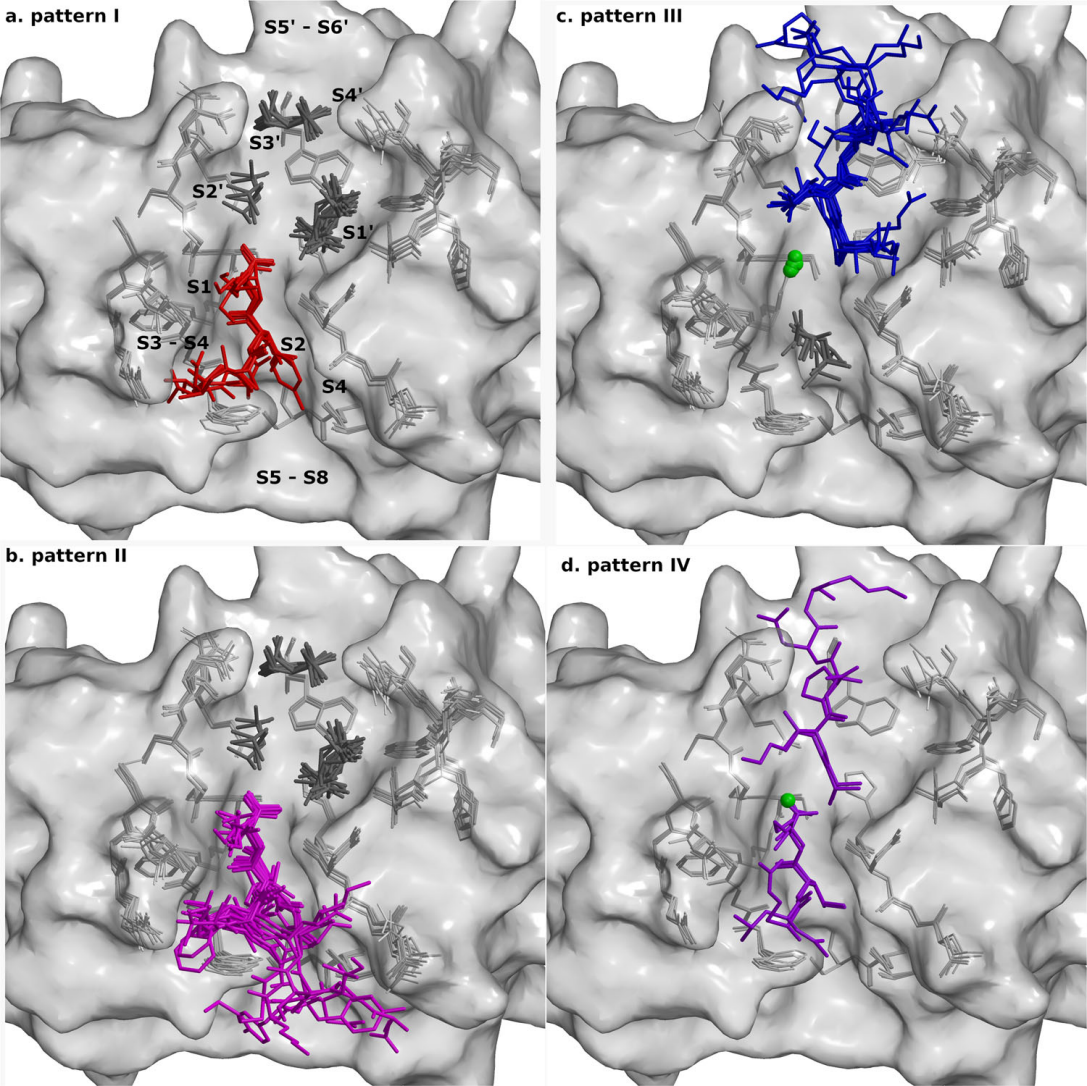
Binding geometry of peptides. Crystal structures of all complexes of cathepsin V were superimposed. Bound peptides are shown on the background of cathepsin V active site residues shown as gray sticks and the surface is shown in semi-transparent gray. The bound peptides are shown as stick models colored according to the patterns (**a**) I (red), (**b**) II (purple), (**c**) III (blue) and (**d**) IV (violet). Chlorine ions are shown as green balls and MPD molecules as dark gray sticks. The figures were generated with MAIN^59^ and rendered with RASTER 3D^66^.

Based on the location of peptide binding and cleavage (Supplementary Table 4), we grouped the peptides in four binding patterns: I. Peptides were cleaved and only their N-terminal fragments remained bound in the non-primed binding sites (Fig. 5a, Supplementary Fig. 8); II. The uncleaved peptides were bound to the non-primed binding sites only (Fig. 5b, Supplementary Fig. 9); III. The uncleaved peptides were bound to the primed binding sites only (Fig. 5c, Supplementary Fig. 10); IV. Peptides were cleaved and both fragments remained bound to the non-primed and primed binding sites (Fig. 5d, Supplementary Fig. 11). Nevertheless, all bound along the active site cleft in accordance with the conserved hydrogen bonding pattern of corresponding positions from P2 to P1'^35^ (Supplementary Fig. 7).

The **pattern I group** (binding of cleaved peptide fragments to the non-primed side) consisted of structures of six peptides that were cleaved, and only their N-terminal fragments remained bound in the non-primed sites S4–S1. Electron density maps of all but one peptide enabled an unambiguous interpretation of the modeled residues from S3–S1, whereas the electron density map for the peptide fragment VACK was weaker, suggesting that the main chain of Ala and Val binds to the S3 binding site. The MPD molecules were bound to the primed site region. The peptide LLKVAL was cleaved and bound to non-primed sites only when co-crystallized, whereas soaking yielded binding in the primed binding area (Supplementary Table 4, pattern I; Fig. 5a, and Supplementary Fig. 8).

The **pattern II group** (binding shifted to the non-primed side) consisted of structures of nine protected peptides and one non-protected peptide that all bound uncleaved into the non-primed sites. Electron density maps of the peptides enabled the unambiguous interpretation of residues from P3–P1. All peptides bound to cathepsin in the same manner, except for fragment TAHE, which bound to cathepsin like fragment VACK (described in the pattern I group). The nitrogen of the amide protection group at the peptide C-terminus was bound approximately 3 Å away from the ND of catalytic H164. The amide likely shared a hydrogen bond with the deprotonated His residue. At P4 and beyond, the features of the electron density maps became weaker and less precise; however, it was possible to model the peptides KPKKKTK, RLSAKP, GNYKEAKK, and EVCKKKK up to P8 in the averaged kick Fo-Fc omit maps^36^. MPD molecules were bound to the primed sites of all structures (Supplementary Table 4, pattern II; Fig. 5b, and Supplementary Fig. 9).

The **pattern III group** (binding shifted to the primed side) consisted of structures of eight non-protected peptides that all bound uncleaved into the primed sites. Electron density maps in the region from P1′–P3′ and partly P4′ of molecule A enabled an unambiguous interpretation. In molecule B, as mentioned previously, the electron density maps enabled the unambiguous interpretation of two further residues, P5′ and P6′, of the peptides AVAEKQ and RLSAKP. On the non-primed side, the MPD molecule occupied the S2 site, and the CL^-^ anion occupied the same position as the carbonyl oxygens of P1 residues in pattern groups I and II. Its negative charge appeared to mimic the absent negatively charged SG atom of the reactive site cysteine. The positively charged amino group of the N-terminal residues of the peptides interacted with the negatively charged CL^-^ and ND of H164 at approximately 3 Å (Supplementary Table 4, pattern III; Fig. 5c and Supplementary Fig. 10).

The **pattern IV group** (binding of cleaved peptide fragments at primed and non-primed side) included structures of two peptides, LLKAVAEKQ and RLSAKP, which were both bound along the active site cleft of cathepsin V. LLKAVAEKQ was designed as a hybrid containing the LLK-fragment from the LLKVAL peptide, which was cleaved and remained bound to cathepsin V on the non-primed side, and AVAEKQ, which bound non-cleaved to the primed side. Overall, their electron densities were weak, and the fragments were refined with partial occupancies. As expected, the fragment LLK bound to the non-primed subsites S3–S1, whereas the fragment AVAEK bound to the primed subsites S1′–S5′, as resolved by the averaged kick omit map. Despite the continuous electron density at the cleavage site, the distance of 2.4 Å between the C atom of Lys at P1 and the N atom of Ala at P1′ was too wide to support a covalent bond between the fragments. However, in the middle there was sufficient space and density to attach the OXT atom to the Lys residue. In the structure of RLSAKP, the fragment RLS bound to the non-primed subsites S3–S1 and the fragment AKP to the primed subsites S1–S3′. The distance of 2.6 Å between C of Ser at P1 and N of Ala at P1′ and the continuous electron density between them resembled the LLKAVAEKQ structure. In both structures, the MPD molecules competed with peptide binding at subsites S2 and S1′–S3′ and CL^–^ ions at the S1 site (Supplementary Table 4, pattern IV; Fig. 5d, and Supplementary Fig. 11).

Peptides from patterns I and IV bound to cathepsin V consistent with the observed protein cleavage sites, whereas the peptides from patterns II and III did not bind according to their cleavage site found in proteins. Their binding appeared to be biased by the absence of the negative charge of the reactive site Cys, which caused a shift in binding. The structure of cathepsins apparently facilitates the presence of a negative charge at the position of C25. In its absence the affinity for the negative charge, as manifested by the binding of Cl^-^ ion, prevailed over amino acid sequence of peptides of group III. Similarly, the binding of C-terminal carbonyl from amide of peptides of group II, as well as the binding of carboxylic terminus from the cleaved peptides of groups I and IV, can be explained. In addition, the differences between the binding of peptides and proteins might reflect the differences between peptide and protein substrate interactions with the protease, which we observed also when we treated peptides with native cathepsins K, V, and L. Several protein and peptide cleavages were the same, whereas a substantial part was unique for protein and peptidyl substrates (Supplementary Data 4 and Supplementary Tables 16 and 17). Peptides were cleaved at more places than proteins, including neighboring cleavages and cleavages at terminal residues. Three sequences TRESEDLE, EVCKKKK, and IILKEK were among examples of cathepsin V single protein cleavages, but they were cleaved two or three times in the peptidyl form. For detailed description of peptide cleavage analysis, see Supplementary Note 1.

### Specific interactions render substrate positions heterogeneous

**Fig. 6:**
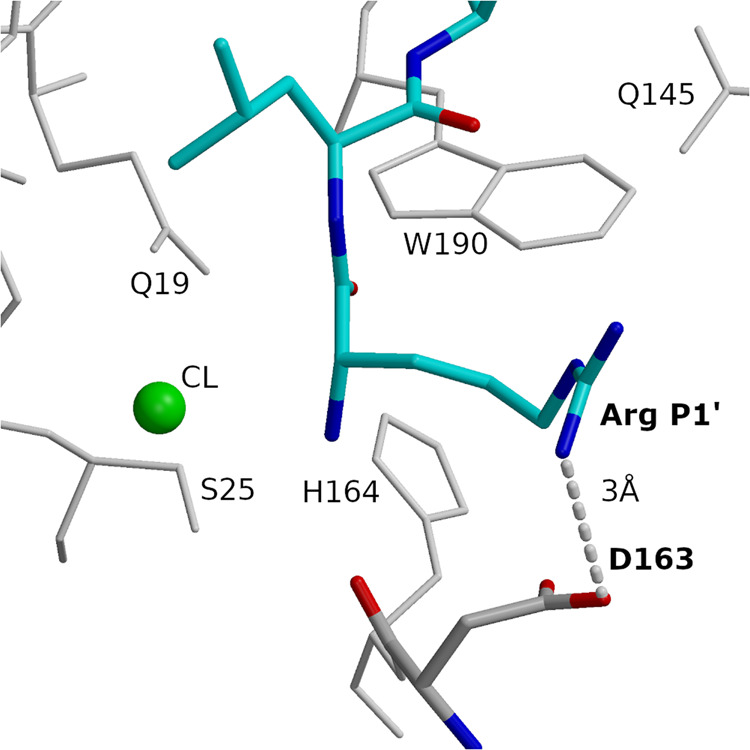
Binding specificity between Arg at P1′ and D163 (cathepsin V). Arg and Leu of peptide RLSAKP (non-protected), bound at subsites S1′ and S2′ are shown in the bond model in blue (nitrogen), red (oxygen), and cyan (carbon). D163 is shown in the bond model in blue (nitrogen), red (oxygen), and gray (carbon). Hydrogen bond is shown with a dashed line. Neighboring residues and the chlorine ion are also provided. Figures in the panel were prepared using MAIN^59^ and rendered using Raster 3D^66^.

**Fig. 7:**
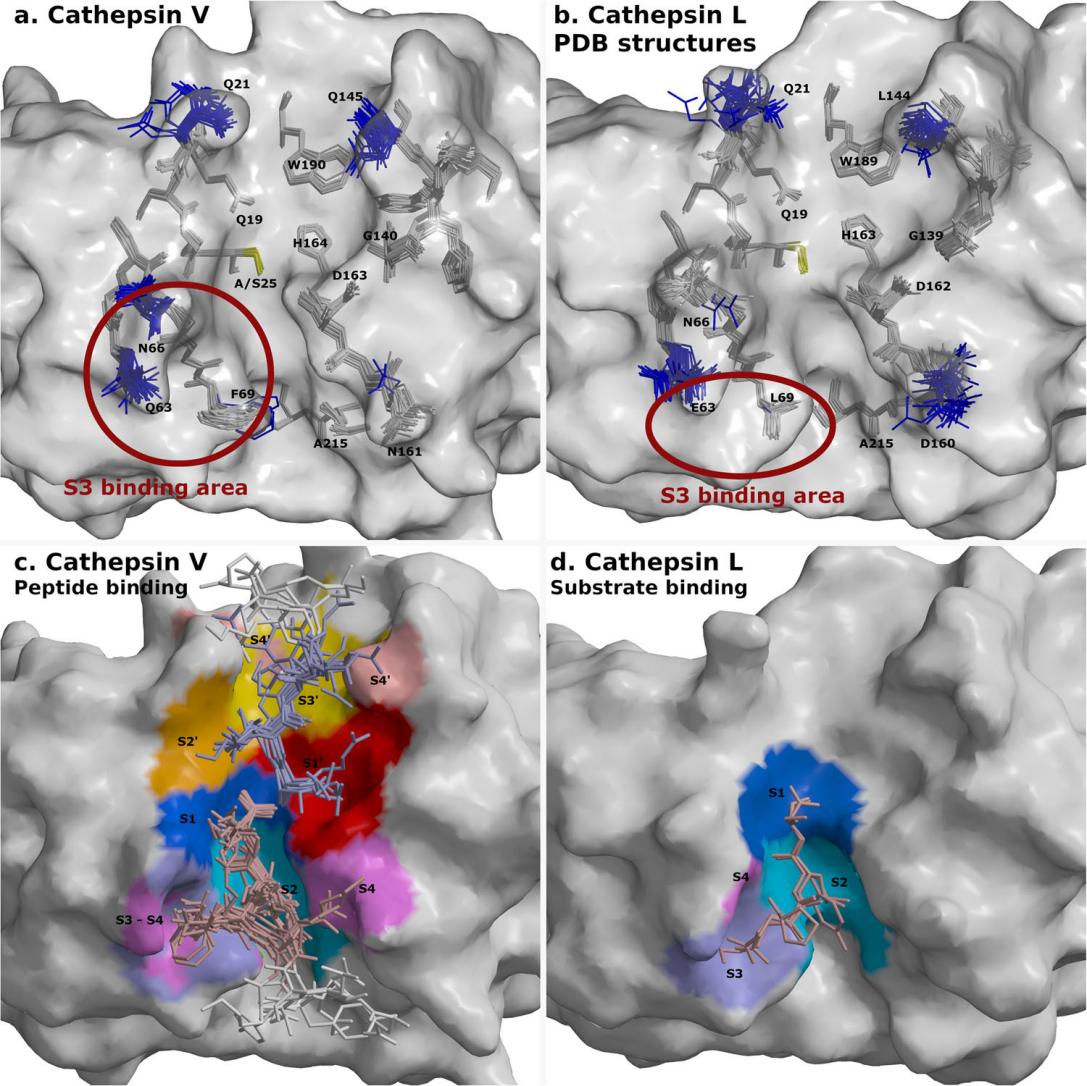
Flexible and rigid residues of cathepsins V and L and their substrate-binding areas. Surfaces of cathepsins V and L are shown in gray. Catalytic residues at site 25 are shown in yellow. Peptides and cathepsin residues are presented as sticks. **a** Superimposed structures of cathepsin V-peptide complexes. Flexible cathepsin V residues that provided versatile binding area for peptide binding are shown in blue. Rigid residues are shown in gray. **b** Superimposed structures of cathepsins L from PDB database with the equivalent labeling (*PDB* entries *1CJL, 1CS8, 1ICF, 1MHW, 2NQD, 2XU1, 2XU3, 2XU4, 2XU5, 2YJ2, 2YJ8, 2YJ9, 2YJB, 3BC3, 3H89, 3H8B, 3H8C, 3HHA, 3HWN, 3K24, 3KSE, 3OF8, 3OF9, 4AXL, 4AXM, 5F02, 5MAE, 5MQY, 6EZP, 6EZX, 6F06, 6JD0*, and *6JD8)*. The structure of C25A mutant with SO42- ion in the active site (*3IV2*) is not included due to distorted active site. Red circles in panels a and b depict S3 binding area of both cathepsins. **c** Binding areas of peptides at positions from P4-P4′ are shown in color spectra from blue to magenta at the non-primed side and from red to rose at the primed side. Peptide residues from P1-P4 and P1′-P4′ are shown in pale pink and pale blue, respectively, whereas the residues beyond P4 and P4′ are in white. **d** Processed peptide and protein substrates of cathepsin L structures (*3K24, 5I4H)* at the non-primed side. Their binding areas are presented with the same coloring annotation as in panel c. The figures were generated with MAIN^59^ and rendered with RASTER 3D^66^.

The hydrogen bonding pattern of P1 and P2 residues restraints the positioning of their side chains, favoring solvent exposure of the P1 residue and binding into the hydrophobic pocket of the P2 residue. The heterogeneity of the P2 position is evidently a consequence of the prevalence of hydrophobic residues. Due to the adverse exposure of their side chains to the solvent molecules, the exclusion of bulky aromatic and hydrophobic residues appears to be the factor behind the heterogeneity of the P1 position. The heterogeneity of the P1′ position in cathepsin V is explained by the specific interaction between the negatively charged D163 residue and the positive charge of the substrate Arg side chain. Their ionic interaction at a distance of 3 Å is visible in the crystal structure of cathepsin V - RLSAKP peptide complex (Fig. 6). Comparison of structures with other cathepsin endopeptidases showed that cathepsins V (D163, Fig. 7a), L (D162, Fig. 7b), and F (D160, Supplementary Fig. 12a) share the Asp and analogously share the heterogeneous positions at P1′ as evident form the prevailing Lys residue in clusters V3, V5, V7, L4 and F2 (Fig. 3). In contrast, cathepsins K (N161 Supplementary Fig. 12b) and S (N163 Supplementary Fig. 12c) have at the equivalent position the neutral Asn residue, which cannot engage in ionic interaction with the substrate’s basic residues such as Arg and Lys, which in turn render their P1′ positions homogeneous.

The flexible side chains of cathepsin V shown in blue (Fig. 7a) provide a versatile binding surface capable of adapting to the binding of different residues at its homogeneous positions. The ambivalence of the Gln and Asn side-chains (Q63 and N66 at S3 and S4, N161 at S4, Q145 at S2′ and S4′ and Q21 at S3′), which provide hydrogen donors and acceptors, are well suited for this purpose. N161 for example assists in the binding of two Lys residues at S4.

The F69 side chain is in the three structures replaced by the Cl^-^ ion at the bottom of the S2 pocket. Furthermore, the binding geometries of the peptides in the regions outside the P2–P2′ range diverged to the extent that the S4 and S4′ surfaces were found in two separate areas on the left and right of the active site cleft (Fig. 7b).

Comparison of all superimposed structures of cathepsin V-peptide complexes and cathepsin L (Fig. 7c, d) structures from PDB (two of them in complex with a peptidyl ligand) exposed a hydrophobic patch on the cathepsin L surface which can explain the heterogeneity of the cathepsin L-specific P3 position evident in the cluster L2 (Fig. 3). The S3 binding area of cathepsin L (elliptical red circle in Fig. 7b) is positioned below its flexible residues E63 and N66, whereas in cathepsin V, residue F69 (L69 in cathepsin L) facilitates the binding of P3 side chains toward residues Q63 and N66. The homogeneity of P3 for cathepsin S and K substrates can likely be explained by the similarity of cathepsin K Y67 and cathepsin S F70 to the equipositioned cathepsin V F69 and the flexibility of cathepsin K D61 and cathepsin S K64 residues (Supplementary Fig. 12).

## Discussion

With the here presented analysis integrating mass spectrometry data and structural biology approaches, we surpassed the current limitations in understanding the yet elusive specificity of cysteine cathepsins. The platform SAPS-ESI was specifically developed for this analysis. A crucial step was the separation of substrate residue positions in heterogeneous, thus selective, and homogeneous, thus non-selective. We achieved this, to the best of our knowledge, with our novel approach based on Anderson-Darling tests for normality of amino acid residue distribution at substrate positions (Fig. 2a). This separation of positions enabled data clustering, which reveal dominant positions and their combinations (Fig. 3, Supplementary Fig. 4), while considering their partially promiscuous character. None of this would have been possible without sufficient amount of data which surpasses the number and diversity of cleavages performed by the cysteine cathepsins stored in the MEROPS database^37^. For comparison, Anderson-Darling analysis of cleavages from the MEROPS database^37^ on a number of cases was unable to clearly differentiate homogeneous from heterogeneous positions (Supplementary Fig. 13). In spite that the number of substrates for cathepsins K, V, L, and S, and some other proteases downloaded from MEROPS were quite large. Exceptions were peptidyl-Lys metalloprotease and glutamine endopeptidase, with a strict P1 specificity, and cathepsins D and E.

Another corner stone was the biologically realistic experimental model, unbiased by the limitations of experimental models that are based on either synthetic^38–40^ or proteome-derived peptide libraries^17,41^. For, example, the proteome based peptide library picked 132 cathepsin K substrates^41^. This study of Schilling and Overall (2006)^41^ indicated preferences of cathepsin K positions for glycine at P1, glutamate at P1′, and aspartate at P5′. For comparison with our study which was based on almost 10,000 cleavages the glycine participation at P1 was noticeable in clusters K1, K2, and K3, however, the P1′ and P5′ positions were not recognized as heterogeneous.

When sequences of clusters individually were mapped into pairs (Supplementary Fig. 14) only a minor fraction of clusters indicated that pairs of residues appear notable – mainly due to the dominance of the prevailing residues. Hence, one can assume that only a small number of cases provide experimental basis for positional cooperativity as described for cathepsin B cleavage sequences with aromatic residues in P2 and P1'^17^. In our analysis of five times larger numbers of cathepsin B cleavages, the pairing of P2 and P1′ residues are evident (Supplementary Fig. 14, B1 cluster) however, the P1 and P1′ positions have higher co-occurrence, for hydrophobic but not aromatic residues though. Consistent with the analysis of Biniossek et al. (2011)^17^, also our analysis shows preference for glycine at P3′ as the dominant residue of the B2 cluster. In contrast our analysis of cathepsin L specificity exposed P3 as heterogeneous position and preference for hydrophobic over aromatic residues at P2. The dominating lysine residues at P1 and P1′ (clusters L3 and L4) was not observed in previous studies. In our study the dominant residue at P2 was leucine (clusters L1 – L4). At positions from P3 to P1′, at P1 and P1′ a preference for small hydrophobic residues such as glycine or alanine frequently appeared (Supplementary Fig. 14, L1-L4 clusters). As our clustering is based on BLOSUM62 alignment score matrix and the score for two sequences determine their evolutionary relation this strongly suggests that for cathepsin L aromatic residues at P2 are not the major specificity factor. Moreover, the pair combination analysis in the column P2-P1 also showed that the pair of hydrophobic/aromatic - positively charged residues at these two positions appear in a limited set of combinations with all other residues. Hence, the characteristic aromatic P2 and positively charged P1 thought as typical pair for cysteine cathepsin substrates exploited in the most commonly used substrate Z-Phe-Arg-AMC^42^ is not the most common sequence. Similarly, the sequences in other commonly used substrates such as Bz-Phe-Val-Arg-NMeC for cathepsins B, S, and L^42^, Z-Gly-Pro-Arg-AMC for cathepsin K^43^, and Z-Val-Val-Arg-AMC for cathepsin S^44^ represent only a fraction of cleavage site variability.

For the cases, such as Granzyme B and caspases, where the P1 position is occupied only by Asp, the prediction of cleavage sites is rather straight forward. However, cysteine cathepsins do not possess such preferences. Also, tools such as PACMANS^45^, Site Prediction^46^, PROSPER^30^, iProt-Sub^18^ are based on SVM. However, to use the self-learning SVM method in the most successful way, it was essential to provide a training set that realistically covered as completely as possible the sequential space of all possible cleavages as well as the optimized list of non-cleavable sequences. Once trained, the SVM-based model was applied to predict cleavages of cysteine cathepsins on several proteins from deadly viruses. The most probable cleavage sites predicted for cathepsins L, S, K and B on the SARS-CoV-2 S protein were experimentally confirmed (Supplementary Fig. 6). The accuracy of our predictions appears to be a considerable improvement when compared with the predictions of PACMANS^45^, which combined prediction of cleavage sites for cathepsins B, K, L, S and V with molecular docking analysis, however, the predicted cleavage positions were not ranked, nor they corresponded to experimentally determined positions, respectively. Moreover, the size of data set effect reliability of predictions. For example, the PROSPER database was built from only 85, 157, 124, and 139 substrates of cathepsins K, L, S and B, respectively^30^, whereas the iProt-Sub database was built from 6,637 cleavages in 3,688 substrates performed by 38 proteases, including cathepsin L as the only representative of cysteine cathepsins, with 17 substrates and 63 cleavages (http://iprot-sub.erc.monash.edu.au/downloads.html)^18^. However, it is not only the size, but also difference in approach. For example, in the iProt-Sub^18^, clustering was used only to avoid bias of similar sequences by removing sequences with 70% or greater identity. In contrast, our statistical analysis indicated that differentiation of heterogeneous and homogeneous positions stabilized after sufficient number of sequences, depending on how representative each selection became (Supplementary Fig. 15). Moreover, our analysis resulted in two cathepsin L clusters, L3 and L4, with dominating lysine residues at P1 and P1′, not observed in previous studies.

Furthermore, having structural insights, enabled us to pinpoint the key residues responsible for heterogeneity of the P1′ position of cathepsins V, L (Fig. 7) and F (Supplementary Fig. 12), and the P3 position of cathepsin L (Fig. 7c). Over 20 cathepsin V-peptide crystal structures exposed interplay between rigid and variable parts of the structure (Fig. 7a). Their analysis indicated that the rigid lock-and-key model of Fischer^47^ may be applicable to the substrate-binding sites that bind the corresponding heterogeneous composition of substrate residues. Additionally, the dynamic nature of proteins, first proposed through the Koshland induced-fit model^48^ and later extended by the model of protein flexibility in solution^49^, which suggests that enzymes adopt different conformations to adjust to different binding partners, is applicable to the binding of residues at homogeneous positions of substrates.

The fact that we were able by using developed platform SAPS-ESI to recognize crucial cathepsin regions and residues that endow them with specific properties suggests the potential relevance of this study for drug discovery projects and for direct application of cleaved sequences in prodrug conjugates, where the selective cleavable linkers are one of the major obstacles for targeted drug delivery. Finally, the proteomics and structural insights combined delivered the concept of heterogeneous versus homogeneous positions which on the one hand points out the sites of selectivity and the complexity of underlaying combinations of patterns indicated by the clusters, and on the other hand provides experimental exposure of inducible conformational changes within the active site of a protease using a modest number of ligands. We think that the approaches demonstrated here can address enzyme and their protein substrate interactions in general.

## Methods

### Experimental assay

Human cysteine cathepsins K, V, L, and S were expressed in *Pichia pastoris*^50,51^, cathepsin F was expressed in baculovirus infected cells^52^, and cathepsin B was expressed in *Escherichia coli*^24^, as described earlier. The concentration of all active enzymes was determined with active site titration with E-64 (cathepsins K, V, L, S, and F) and CA074 (Cathepsin B). Human SH-SY5Y neuroblastoma cells were cultured and isotopic forms of arginine were used for stable isotope labeling by amino acids (SILAC)^22^ for each cathepsin. From every culture a given number of cells were harvested and lysed by three rounds of freeze-thawing. The mass of proteins in each sample was approximately 400 µg. After the samples were acidified to pH 5.5 by adding HCl, they were exposed to cathepsins at 37 °C at 200 nM final concentration. After 20 min, cathepsins K, V, L, S, or F were inhibited by the addition of E-64 and cathepsin B by the addition of CA074. Solid guanidinium chloride was added to a concentration of 4 M to all samples to denature all proteins. Protein mixtures were then reduced and alkylated by addition of 15 mM tricarboxyethylphosphine and 30 mM iodoacetamide and incubated for 20 min at 30 °C to block all activity of cathepsins. The separation and identification of neo-N-terminal peptides as proxies for cleavage sites and substrates were performed as described^21,53–56^. In brief, the cathepsin induced cleavages were determined using consecutive reverse phase high performance liquid chromatography (RP-HPLC) and liquid chromatography-tandem mass spectroscopy (LC–MS/MS) analysis on an LTQ Orbitrap XL or Orbitrap Velos mass spectrometer (Thermo) by applying the N-terminal Combined Fractional Diagonal Chromatography (COFRADIC) protocol. The recorded MS/MS spectra were searched using the Mascot program^57^ in the SwissProt and TrEMBL databases^58^, to identify cathepsin-derived neo-N-terminal peptides. The identified peptides were evaluated and stored in data sets separately for each cathepsin. The data sets for further analysis consisted of 30 residue-long peptides that corresponded to the sites from P15 to P15′ (Supplementary Table 1). For each identified UniProt protein sequence, we gathered available PDB protein database codes^23^ by using the SwissProt/TrEMBLe search tool (Retrieve/ID mapping) and the corresponding protein sequence length, mass and name information. The corresponding PDB entries were downloaded using PDB tools.

### Cleavage site separation

The distributions of separations between the cleavage sites of each cathepsin and between the cleavage sites of all combinations of pairs of cathepsins were analyzed by counting the residues between each pair of cleavage sites. The separations from P1 to cleavages at the primed and non-primed sides were labeled as positive and negative, respectively. For example, the separation between the cleavages at P1 and P2′ was 2, and that between P1 and P2 was −1 (Supplementary Fig. 1a). The distribution of separations was generated with code written in Fortran 90 (Supplementary Fig. 1b, c).

### Share of cleavages that were exposed to the solvent

The accessible surfaces were calculated using MAIN with a solvent sphere size of 1.6 Å^59^. The accessible surfaces of residues at positions P1 and P1′ were divided into three groups: not exposed (surfaces were zero at P1 and P1′), exposed (not zero at positions P1 and P1′) and partially exposed (zero at P1 or P1′) (Supplementary Fig. 1d). The share of cleavages that were exposed to the solvent was generated by combining scripts for statistical analyses of the cleavage sites in the secondary structures in the MAIN program^59^ and codes that were written in Fortran 90.

### Share of cleavages in α-helices, β-sheets and loops

Secondary structures of residue assignment were obtained from PDB entries and further used in the analysis of the locations of cleavage sites in α-helices, β-sheets and loops using MAIN^59^. The shares were obtained for three cases: positions P1 and P1′ were in α-helices or β-sheets, and the rest were declared loop locations (loop P1 – loop P1′, loop P1 – α-helices P1′ and vice versa, and loop P1 - β-sheets P1′ and vice versa) (Supplementary Fig. 1e). The shares of cleavages in α-helices, β-sheets and loops were generated by combining scripts for statistical analyses of the cleavage sites in the secondary structures in the MAIN program^59^ and codes in Fortran 90.

### Clustering of cleavage sites

For clustering of cleavage sites of cysteine cathepsins, we conducted statistical clustering with codes that were written in SAS for Windows^60^. The criteria that were used in the optimization process of the clustering were the cubic clustering criterion (CCC) and sufficient number of well defined clusters (definition is in Results section). Less well-defined clusters had at heterogeneous positions at least 20% of residues from the same chemical group (Fig. 3, for example cluster **K1** after the first branching and site **P1**). The success of the optimization outcome was measured by the increase in the share of well-defined clusters with CCC values of higher than 2. The clustering process consisted of three main steps: **1)** scoring of amino acid residues before clustering and optimization of the clustering algorithm design based on **2)** the selection of variables and **3)** the choice of algorithm.

1. We calculated the scoring function values of aligned pairs of residues between the cleaved peptide and the data set representative peptide (the most frequent residues from the set in the position span from P15 to P15′) using BLOSUM and PAM matrices. The BLOSUM62 matrix^26^ was finally selected because it delivered the best final outcome.
2. The variables for clustering were positions. The highest number of well-defined clusters was obtained by the selection of heterogeneous positions that were determined with the Anderson–Darling test (Fig. 2).
3. Optimization of clustering was performed by comparing the outcomes of the clustering algorithms based on the squared Euclidian distances between cluster centers (representative peptides of clusters), between similar peptides – one in each cluster, or between two very different peptides – one in each cluster; based on the average squared Euclidian distances between all pairs of peptides in the two clusters; or using Ward’s minimum variance method. Finally, Ward’s minimum-variance method algorithm for the bottom-up approach was selected because it delivered the highest share of well-defined clusters.

For the individual cluster justification, the combinations of 20 amino acid residues at combinations of positions in the region from P5 to P5′ were counted. There were 45, 120, 210, and 252 possible combinations of 2, 3, 4, and 5 positions, respectively. At each combination of positions, we explored all possibilities of combinations of residues; interestingly, not all combinations of amino acid residues appeared. Triplet, quadruple, and quintet residue combinations were sparsely populated in comparison to pairs; hence, the information content there was small. The combinations of residues at combinations of positions in the span P5 to P5′ were generated with codes that were written in Fortran 90 and bar codes in SAS for Windows^60^. The results for combinations of 2 positions and belonging amino acid residues for 30 clusters are presented in Supplementary Fig. 14.

### Predictions of cleavage sites

SVM models were developed using the PCSS server in the “training” mode^16^. In short, the PCSS server used three criteria to select the best quality comparative model to evaluate secondary structure and solvent exposure for a peptide in a protein: model score (the larger fraction of the target was included to obtain a general score for the reliability of the SVM model), model coverage (the fraction of the target protein sequence for which a comparative model was generated) and predicted native overlap. We used the predicted native overlap, namely, the fraction of Cα atoms in the model that were predicted to be within 3.5 Å of the native state. For training, we prepared lists of “positive” and “negative” peptides that were composed of eight amino acid residues from P4 to P4′ and optimized their compositions by preparing various numbers of testing peptides. For each data set, the positive peptides were selected from the cleaved peptides that belonged to all clusters of one cathepsin, whereas the negative peptides were selected from the regions of proteins that had at least 30 positions without identified cleavage sites of any of the cathepsins. On average, the number of negative peptides exceeded the number of positive peptides by 1,500 (Supplementary Table 2a). The random predictor line was between (0,0) and (1,1); the perfect line was from (0,0) over (0,1) to (1,1). The critical point was the intersection between the receiver operating characteristic (ROC) plot (Supplementary Fig. 5) and the line from (0,1) to (1,0). ROC plots were created by plotting the true positive rate (TPR) or sensitivity or probability of detection of cleavages against the false positive rate (FPR). They were programmed in SAS for Windows^60^. The PCSS server in the “application” mode was used for the prediction of cleavages (Supplementary Data 3).

### In vitro processing of mutated and wild-type S protein of SARS-CoV-2 by cathepsins B, L, K, V, and S

The recombinantly expressed extracellular domain of the SARS-CoV-2 S protein was purchased from GenScript (cat. number: Z03481-100). A soluble SARS-CoV-2 S protein version with a mutated furin cleavage site and 2 stabilizing proline mutations (K986P, V987P) in the S2 domain, called S-2P (Table I^61,62^ shows an overview of different stabilized S proteins, including the “S-2P” nomenclature for this variant), was produced in ExpiCHO-S cells. Upon harvest, the protein was purified by immobilized metal ion affinity chromatography (HisTrap^TM^ HP column), desalted by using a HiPrep^TM^ 26/10 column, concentrated on a Vivaspin®20 centrifugal concentrator (100,000 MWCO) and finally filtered over a low protein binding 0,2 µm filter. Recombinant cathepsins B, K, L, S, and V were expressed as described in methods. Cathepsins were activated in 50 mM MES pH 5.5, 100 mM NaCl, 1 mM EDTA, and 2.5 mM DTT. For the in vitro cleavage assay, the SARS-CoV-2 S protein was diluted in a reaction buffer that contained 50 mM MES pH 6.5, 100 mM NaCl, 1 mM EDTA, and 2.5 mM DTT to a concentration of 0.25 µg/µl. Cathepsins K, L, S or V were added at an E:S molar ratio of 1:20 and cathepsin B at a 1:5 ratio. The reaction mixtures were incubated at 37 °C for various time periods, inactivated by the addition of SDS–PAGE loading buffer and incubated at 95 °C for 2 min. For the control samples, cathepsins K, B, L, S, and V were inhibited by the addition of 25 µM E-64 to the activation reaction. Protein samples were separated by sodium dodecyl sulfate–polyacrylamide gel electrophoresis (SDS–PAGE) and stained by Coomassie staining or transferred to PVDF membranes for N-terminal sequence analysis.

### N-terminal sequence analysis of samples

Sequence analysis of samples of wild type (WT) and furin cleavage site mutated (MUT) S protein by cathepsins B, K, L, S, and V was performed at the Department of molecular and biomedical sciences at JSI, using a PPSQ-53A Gradient System apparatus (Shimadzu, Japan) that consisted of the following modules: a PPSQ-53A protein sequencer; an HPLC unit for analysis of PTH amino acid with LC-20AD binary pumps and a DGU-20A3R degasser; an SPD-M30A PDA detector; a thermostatic chamber for a CTO-20AC HPLC column, and the LabSolutions PPSQ computer program for the collection and analysis of data. The Edman method was used to prepare PTH amino acid derivatives. Analysis of PTH derivatives was performed on a Wakopak® Wakosil PTH-GR (S-PSQ) column (2.0 × 250 mm) (FUJIFILM Wako Pure Chemical Corporation, Japan). Solvents and reagents were of sequential purity (FUJIFILM Wako Pure Chemical Corporation, Japan).

### Peptide selection

Thirty sequences representing cathepsin V substrates (Supplementary Table 3, p1–p30) were selected among clusters V1 – V7:

- They contained various residues at cathepsin V heterogeneous positions P2, P1, and P1′ and represented all seven cathepsin V clusters. Clusters were named using cathepsin IDs, followed by their consecutive numbers. Cathepsin V clusters have the following pattern: the prevailing residue Leu at P2 (V1), Lys at P1 (V2), Lys at P1′ with either hydrophobic (V3) or hydrophilic residues (V5) at P2, hydrophobic residues other than Leu at P2 (V4), Lys at P1 and P1′ (V7), and the remaining cluster (V6).
- They contained sequences from shared cleavage sites (cleaved by several cathepsins) and from unique cathepsin V cleavages (cleaved by cathepsin V only).
- They contained sequences from cleavage areas (cleavages appeared adjacent to each other) and specific sites (no additional cleavages were observed in the neighborhood).
- They were of different lengths, from six to ten amino acid residues.

Four sequences that did not match any sequence from protein analysis, as well as the sequence LLKAVAEKQ, which was engineered as a hybrid of the two, were included in the synthesis (p31–p35). To mimic the polypeptide chain, the termini of most peptides from p1–p31 were protected by N-acetylation and C-amidation to neutralize charges. To assess the role of the charged termini, ten peptides (p7–p9, p14, p19, p23, and p32–p35) were also synthesized without termini protection. This yielded 41 synthesized peptides. Peptides p1 and p2 cannot be dissolved after synthesis; therefore, they were not used in this study.

### Peptide synthesis and purification

The peptides were synthesized using standard solid-phase fluorenylmethyloxycarbonyl chloride (Fmoc) chemistry on a SyroI (Biotage) instrument. The synthesis was started on 25 µmol of rink amide resin (Novabiochem). Amino acids were coupled in a 4-fold excess using HOBT/HBTU activation. The peptides were cleaved using TFA containing phenol, triisopropylsilane, and 5% water for 3 h. The peptides were then precipitated with tributyl methyl ether and recovered by centrifugation at 2000 × g. The ether washing/centrifugation step was repeated three times.

### Peptide purification

Peptides were purified on semi a semi-preparative Nucleodur C18 column (Macherey-Nagel) on RP-HPLC system with mobile phases A (0.1% v/v TFA in Milli-Q water) and B (0.1% v/v TFA in ACN). Gradient elution was applied at 0.5–2% ACN/min, depending on each peptide impurity profile. The separation was monitored at wavelengths of 214 and 280 nm. Purified peptides were then collected, placed on a Speedvac to remove ACN, lyophilized, weighed, and stored at –80 °C until further use.

### Procathepsin V expression and purification

All proteins were cloned into the pPIC9 vector and expressed in *P. pastoris* strain GS115 according to the Invitrogen Pichia Expression Kit (Invitrogen, K1710-01). Mutations were introduced at glycosylation sites N108Q and N179Q and at catalytic site C25S or C25A. The expression, purification, and activation protocols for cathepsins V were based on previously described procedures^33^. The expression medium of the procathepsin V mutant was concentrated to approximately 300 mL and dialyzed three times against activation buffer (100 mM NaOAc, pH 5, 1 mM EDTA, and 5 mM DTT). 5% (n/n) of activated cathepsin L was added to the sample to remove the propeptide region from the cathepsin V mutant. Activation was stopped the next morning with the addition of approximately 10-fold molar excess of inhibitor E-64 relative to cathepsin L. The sample was then applied to SP-Sepharose FF, where the cathepsin V mutant was eluted with 400 mM NaCl. The sample was then dialyzed into crystallization buffer (20 mM NaOAc, pH 4.5, 10 mM NaCl, 1 mM DTT, and 5% glycerol), concentrated to approximately 40 mg/mL, and stored at –80 °C until crystallization trials.

### Cathepsin V crystallization

For crystallization purposes, two active site mutants of cathepsin V were prepared: one with Cys 25 mutated to Ser and the other to Ala. Crystals of both mutants were grown in 77% of 2-methyl-2,4-pentanediol (MPD) and 23% of 60 mM (hydroxymethyl)aminomethane (Tris) buffer (pH 8.0). The sitting drop technique was used for crystallization, and the crystals grew from different solutions at 5 °C, all of which contained precipitant MPD. The optimized conditions were 23% of 60 mM TRIS, pH 8, and 77% of MPD. The first crystals appeared after 24 h and continued to grow for 1–2 weeks.

### Peptide soaking and co-crystallization

Stocks of lyophilized peptide powders were prepared in 60 mM Tris at pH 8 (hereafter referred to as peptide tris stocks). The final peptide molarity in the stocks was 20–90 mM, depending on the solubility properties of each peptide. Peptides were soaked in pre-formed cathepsin V mutant crystals by adding a drop of crystallization solution, made of MPD and peptide tris stock, on top of the crystals. At time points of approximately 1, 10, and 24 h, multiple crystals were collected, and the best diffracting crystal was used for structure determination. Similarly, the tris buffer component was replaced with a peptide tris stock for peptide co-crystallization. The crystals were harvested and flash-cooled after they reached their final size.

### Data collection, structure determination, and refinement

Data were collected at BESSY (Berlin) and Elettra (Trieste) synchrotrons under cryogenic conditions (100 Kelvin). Data from the best diffracting crystal of each cathepsin-peptide complex were collected and processed using XDS software^63^. Crystals with space group P43212 diffracted from 1.3 to 2.1 Å and contained two molecules in the asymmetric unit, referred to as molecules A and B (RMSD between them was ~0.4 Å). The first structure was solved by molecular replacement with Phaser^64^ using PDB ID entry 3H6S as the model. All the subsequent structures were determined using the first solved structure as the starting model. MAIN software^59^ was used for the subsequent steps of structure determination: map calculation, model building, refinement, validation, and deposition. For refinement, the maximum likelihood (ML) free-kick target function was applied, which uses all structural factors instead of a fraction of the test set of data for phase error estimates calculation^65^. Thus, the atom coordinates are slightly perturbed (kicked) to free the model from its bias. Therefore, the α and β of the ML target function were more accurately assessed. For map calculation, the model is returned to the state before the kick, and α and β values are applied. Ramachandran plot showed that 95–98% of residues bound to favorable regions, 5–2 % of residues to allowed regions, whereas three structures had one outlier (Cathepsin V – VPCGTAGE, Cathepsin V – ALAASS and Cathepsin V – VACKSSQP complexes) and two structures had two outliers each (Cathepsin V – LLKAVAEKQ and Cathepsin V – QLRQQE complexes). Cathepsin V – peptide complexes were deposited on Protein Data Bank (PDB) and were assigned following entries: Cathepsin V – EVCKKKK (7Q8H), Cathepsin V – TRESEDLE (7Q8D), Cathepsin V - GNYKEAKK (7Q8F), Cathepsin V – VPCGTAHE (7Q8L), Cathepsin V – KPKKKTK (7Q8M), Cathepsin V – KKYDAFLA (7Q8N), Cathepsin V – AVAEKQ (7Q8I), Cathepsin V - RLSAKP (protected; 7Q9C), Cathepsin V - RLSAKP (non-protected; 7Q8Q), Cathepsin V - LLKAVAEKQ (7Q9H), Cathepsin V - GAKSAA (7QHJ), Cathepsin V – LLKVAL (co-crystallized; 7Q8K), Cathepsin V - LLKVAL (soaked; 7Q8P), Cathepsin V - VACKSSQP (7QFF), Cathepsin V - AYFKKVL (7QFH), Cathepsin V - ALAASS (7Q8G), Cathepsin V – LLSGKE (7Q8O), Cathepsin V – IILKEK (7Q8J), Cathepsin V - QLRQQE (7QHK), Cathepsin V - VYEKKP (7QNS), and Cathepsin V - GAKSAA (7QO2). Structural images were generated with MAIN^59^ rendered with RASTER 3D^66^.

### Peptide cleavage analysis of cathepsins V, L, and K

Peptides were treated with cathepsin K and L (1 μM) and cathepsin V (2 μM) in a buffer consisting of 30 mM NaOAc, 30 mM NaCl, 5 mM DTT and pH 5.5 at 37 °C for 2 h. We empirically chose the concentration of each peptide to be used in the assay based on the abundance of its signal on RP-HPLC. The cleaved peptide fragments were separated on an analytical Nucleodur C18 column (Macherey-Nagel). Mobile phases A and B consisted of 0.1% TFA in deionized and degassed H_2_O and acetonitrile (ACN), respectively. The separation of fragments was followed by the absorption of peptide bonds at a wavelength of 214 nm or absorption corresponding to the Trp and Tyr residues at a wavelength of 275 nm. Peptides were eluted with an ACN gradient of 1% / min, starting with 5% of ACN. Several peptides were not retained in the column but were captured in the void volume. Others were eluted at ACN concentrations ranging from 5 to 35%. The sequence of each collected fragment and the cleavage sites of the treated peptides were determined using MALDI-TOF mass spectrometry.

### Sample preparation and MS MALDI-TOF/TOF analysis

HCCA matrix (1.4 mg/mL) was prepared in a solvent mixture of 85% acetonitrile, 15% water, 0.1% TFA, and 1 mM NH_4_H_2_PO_4_. For sample preparation, equal volumes of captured separated peptide fragments from RP-HPLC and the matrix solution were mixed, and 1 μL was applied to the ground steel plate. The solution was dried at 20 °C prior to measurement. Mass measurements were performed using an UltrafleXtreme III MALDI-TOF/TOF mass spectrometer (Bruker; Billerica, MA, USA). The spectra were acquired, processed, and calibrated using FlexControl 3.0 and FlexAnalysis software (Bruker). The parameters used for positive ion measurements in the range from 0 to 3500 Da were the following: ion source 1, 25 kV; ion source 2, 22.30 kV; lens, 7.5 kV; reflector, 26.4 kV; reflector 2, 13.3 kV; pulsed ion extraction, 60 ns; and reflector detector voltage, 2230 V. External calibration was carried out using bradykinin (1–7), angiotensine I and angiotensine II and internally using 4-HCCA.

### Superimposition of cathepsins K, L, S, and F from PDB database

Search across the PDB database was conducted using 95% cutoff sequence identity with wild-type cathepsins K, L, S, and F. All the entries that satisfied the criteria were downloaded and superimposed using MAIN software^59^. The procathepsin entries that satisfied the search criteria were excluded from the comparison.

### Statistics and reproducibility

The existing datasets of substrates of cathepsins K (9583), V (4415), B (4254), L (4117), S (3805), and F (3500) were used for analysis. For modelling by using Support Vector Machine algorithm the training set consisted of “positive” peptides of cathepsins K (2253), V (2081), B (4006), L (1938), S (1792), and F (3277) and “negative” peptides 3526 (K), 4508 (V), 4508 (B), 3948 (L), 3526 (S), and 4508 (F) separately for individual cathepsins. The total number of peptides of cathepsins K, V, B, L, S, and F for testing of SVM models were 10,466, 2002, 1326, 1978, 1773, and 993, respectively.

For validation of heterogeneous and homogeneous positions the substrates of K (2190), V (1649), B (581), L (2911), and S (3112) cathepsins downloaded from MEROPS (https://www.ebi.ac.uk/merops/) were used. Additionally, heterogeneous and homogeneous positions were tested for substrates of the following enzymes: peptidyl-Lys metallopeptidase (2104), caspase-3 (680), granzyme B (1900), caspase-7 (499), glutamyl endopeptidase I (4324), cathepsin E (1586), cathepsin D (899), and cathepsin G (447).

35 selected peptides of cathepsin V were used for determination of complexes cathepsin V - peptide. Identification of cleaved selected peptides (28) by cathepsins K, L, V resulted in 150 peptides.

In the case of clusters, 4 peptides out of 4254 of cathepsin B and 4 peptides out of 4117 for cathepsin L were excluded because they didn’t have all heterogeneous positions. An example of excluded peptide is peptide M P - V K K K R K S P G V A A A V A.

In the case of peptides selected for training SVM models, some peptides were excluded due to mismatches between peptide sequences read from the input file and what was stored in modbase of PCSS server. From training sets for SVM models 80 proteins were excluded in total because they contained some of 35 peptides selected for structural studies (21) and additional cleavages of native cathepsins K, V, and L (sample of 150 peptides). S protein of SARS-CoV-2 in total was not included into training sets of any cathepsin.

Anderson-Darling test for normality was calculated and described in Methods as well as clustering and development of SVM models.

### Reporting summary

Further information on research design is available in the Nature Portfolio Reporting Summary linked to this article.

## Supplementary information

Supplementary Information (pdf file)

Supplementary Data 1

Supplementary Data 2

Supplementary Data 3

Supplementary Data 4

Reporting Summary

## Data Availability

Data sets of K, V, B, L, S, and F cathepsins substrates (excel files), SVM models (zip file with txt files) and results of HPLC analysis (pdf file) are available as Supplementary Data 1 to 4. The data sets from MEROPS database are publicly available (https://www.ebi.ac.uk/merops/). Cathepsin V – peptide complexes were deposited to Protein Data Bank (PDB)^24^ and were assigned following entries: Cathepsin V – EVCKKKK (7Q8H), Cathepsin V – TRESEDLE (7Q8D), Cathepsin V - GNYKEAKK (7Q8F), Cathepsin V – VPCGTAHE (7Q8L), Cathepsin V – KPKKKTK (7Q8M), Cathepsin V – KKYDAFLA (7Q8N), Cathepsin V – AVAEKQ (7Q8I), Cathepsin V - RLSAKP (protected; 7Q9C), Cathepsin V - RLSAKP (non-protected; 7Q8Q), Cathepsin V - LLKAVAEKQ (7Q9H), Cathepsin V - GAKSAA (non-protected; 7QO2), Cathepsin V - GAKSAA (protected; 7QHJ), Cathepsin V – LLKVAL (co-crystallized; 7Q8K), Cathepsin V - LLKVAL (soaked; 7Q8P), Cathepsin V - VACKSSQP (7QFF), Cathepsin V - AYFKKVL (7QFH), Cathepsin V - ALAASS (7Q8G), Cathepsin V – LLSGKE (7Q8O), Cathepsin V – IILKEK (7Q8J), Cathepsin V - QLRQQE (7QHK), and Cathepsin V - VYEKKP (7QNS). The mass spectrometry proteomics data have been deposited to the ProteomeXchange Consortium via the PRIDE^67^ partner repository with the dataset identifier PXD041185 and 10.6019/PXD041185.

## Code availability

Data sets were processed with SAPS-ESI software platform (Statistical Approach to Peptidyl Substrate-Enzyme Specific Interactions) consisting of codes in GNU fortran 10.2.0 (gcc 10.2.0) and SAS for Windows (SAS 9.4 TS Level 1M7, X64_10PRO platform, licensed to CIPKeBiP, site 70126232), with programs MAIN (released in 2021) and PCSS server (https://modbase.compbio.ucsf.edu/peptide/). The results were post-processed, analyzed and plotted by using SAS for Windows (SAS 9.4 TS Level 1M7, X64_10PRO platform, licensed to CIPKeBiP, site 70126232), MAIN program (release in 2021 https://www-bmb.ijs.si/), RASTER 3D 3.0 (September 2020 http://www.bmsc.washington.edu/raster3d/), Microsoft Office LTSC Professional Plus 2021: Excel (licensed to IJS) and Microsoft Visio Premium 2010 (licensed to IJS). Computer codes in SAS and Fortran as part of SAPS-ESI platform are available upon request. MAIN program and RASTER 3D 3.0 are publicly available. The following procedures was implemented by publicly available software: a. Identification of peptides of cathepsins K, V, B, L, S, and F: The recorded MS/MS spectra were searched with MASCOT using the MASCOT Daemon interface (version 2.4.0, www.matrixscience.com) in the Swiss-Prot database (release of November 11, 2011, January 25, 2012 and April 18, 2012). Potential false positive peptide identifications were selected and automatically removed by the Peptizer software application. Neo-N-terminal peptides were then loaded into the TOPPR database, and re-mapped onto the latest version of the Swiss-Prot database (release of January 22, 2014) to generate final lists of total cleavage sites (Supplementary Data 1). All together 29,674 peptides were identified. b. By using search tools in UniProt database (https://www.uniprot.org/) for 29,674 peptides (originated from 3167 proteins) PDB codes were gathered and later downloaded from PDB protein data bank by using their program tools (https://www.rcsb.org/). We downloaded structures determined by x-ray (1592) (in January 2019). c. MEROPS data sets of cathepsins K, V, B, L, and S substrates (10,443) were downloaded in August 2022 (https://www.ebi.ac.uk/merops/). d. MEROPS data sets of substrates for the following enzymes peptidyl-Lys metallopeptidase, caspase-3, granzyme B, caspase-7, glutamyl endopeptidase I, cathepsin E, cathepsin D, cathepsin G substrates (12,439) were downloaded in August 2022. e. 21 structures of complexes cathepsin V - peptides were determined by using XDS Program Package (Version February 2021 https://xds.mr.mpg.de/), PHASER-2.8.3 (CCP4: Supported program https://www.ccp4.ac.uk/html/phaser.html), MAIN program (release in 2021 https://www-bmb.ijs.si/) and RASTER 3D 3.0 (September 2020 http://www.bmsc.washington.edu/raster3d/). f. Identification of cleaved selected peptides (28) by cathepsins K, L, V: the MS spectra were acquired, processed and calibrating by using FlexControl 3.0 program and FlexAnalysis software (Bruker). All together 150 peptides were identified.
