# Supplementary material for: Proteomic data and structure analysis combined reveal interplay of structural rigidity and flexibility on selectivity of cysteine cathepsins
Source: Commun Biol. 2023 Apr 24;6:450. doi: 10.1038/s42003-023-04772-8 (PMC10124925; doi:10.1038/s42003-023-04772-8)
Supplement: Supplementary file 1 — Supplementary Information (pdf file) [file 42003_2023_4772_MOESM1_ESM.pdf]

# Proteomic data and structure analysis combined reveal interplay of structural rigidity and flexibility on selectivity of cysteine cathepsins

Livija Tušar[1,2*], Jure Loboda[1,3*], Francis Impens[4*], Piotr Sosnowski[2], Emmy Van Quickelberghe[4], Robert Vidmar[1], Hans Demol[4], Koen Sedeyn[5], Xavier Saelens[5], Matej Vizovišek[1], Marko Mihelič[1], Marko Fonović[1], Jaka Horvat[6], Gregor Kosec[6], Boris Turk[1,7], Kris Gevaert[4**], Dušan Turk[1,2**]
*These authors contributed equally.
**Corresponding authors, e-mails to kris.gevaert@vib-ugent.be and dusan.turk@ijs.si

[1] Jožef Stefan Institute, Department of Biochemistry and Molecular and Structural Biology, Jamova cesta 39, 1000 Ljubljana, Slovenia
[2] Centre of Excellence for Integrated Approaches in Chemistry and Biology of Proteins (CIPKeBiP), Jamova cesta 39, 1000 Ljubljana, Slovenia
[3] The Jožef Stefan International Postgraduate School, Jamova cesta 39, 1000 Ljubljana, Slovenia
[4] VIB-UGent Center for Medical Biotechnology and UGent Department of Biomolecular Medicine, Technologiepark-Zwijnaarde 75, 9052 Ghent, Belgium
[5] VIB-UGent Center for Medical Biotechnology and, Department for Biochemistry and Microbiology, Ghent University, 9052 Ghent, Belgium
[6] Acies Bio d.o.o., Tehnološki park 21, 1000 Ljubljana, Slovenia
[7] Faculty of Chemistry, University of Ljubljana, Večna pot 113, SI-1000 Ljubljana, Slovenia

## Supplementary Information

| | |
|---|---|
| Supplementary Fig. 1. | Cleavage site separations and cleavage locations. |
| Supplementary Fig. 2. | Protein with unique and shared cleavage sites. |
| Supplementary Fig. 3. | Single cleavage cases mapped on the 3D structures. |
| Supplementary Fig. 4. | iceLogo plots of datasets of substrates of cathepsins K, V, B, L, S, and V and belonging 30 clusters. |
| Supplementary Fig. 5. | The receiver operating characteristic (ROC) plot. |
| Supplementary Fig. 6. | SDS page analysis of SARS-CoV-2 S protein degradation/processing; Cleavages of SARS-CoV-2 wild-type (WT) and mutated furin cleavage site (MUT) S proteins. |
| Supplementary Fig. 7. | H-bonding pattern between cathepsin V and substrates. |
| Supplementary Fig. 8. | Electron density maps of peptides in the group of pattern I. |
| Supplementary Fig. 9. | Electron density maps of peptides in the group of pattern II. |
| Supplementary Fig. 10. | Electron density maps of peptides in the group of pattern III. |
| Supplementary Fig. 11. | Electron density maps of peptides in the group of pattern IV. |
| Supplementary Fig. 12. | Flexible and rigid residues of cathepsins K, S and F from PDB database. |
| Supplementary Fig. 13. | Heterogeneous and homogeneous positions of substrates for selected enzymes downloaded from MEROPS database. |
| Supplementary Fig. 14. | Combinations of pair positions with pair residues (cooperativity) for clusters of substrates of cathepsins K, S, V, B, F, and L. |
| Supplementary Fig. 15. | Heterogeneous and homogeneous positions for different shares of peptides selected from clusters for cathepsins K, V, B, L, S, and F. |
| Supplementary Table 1. | Summary of the data sets of peptides for cathepsins K, V, B, L, S, and F. |
| Supplementary Table 2. | Parameters of support vector machine SVM models and predictions of cathepsins' cleavage sites. (a) SVM models. |
| Supplementary Table 3. | Selected peptide sequences for complexes with mutatedcathepsin V. |
| Supplementary Table 4. | Summary of peptide binding to crystals of cathepsin V C25S/A. |
| Supplementary Table 5. | Data collection and refinement statistics: PDB entries 7Q8H and 7Q8D. |

Supplementary Table 6. Data collection and refinement statistics**:** PDB entries 7Q8F and 7Q8L.
Supplementary Table 7. Data collection and refinement statistics**:** PDB entries 7Q8M and 7Q8N.
Supplementary Table 8. Data collection and refinement statistics**:** PDB entries 7Q8I and 7Q9C.
Supplementary Table 9. Data collection and refinement statistics**:** PDB entries 7Q9H and 7QHJ.
Supplementary Table 10. Data collection and refinement statistics**:** PDB entries 7Q8K and 7Q8P.
Supplementary Table 11. Data collection and refinement statistics**:** PDB entries 7QFF and 7QFH.
Supplementary Table 12. Data collection and refinement statistics**:** PDB entries 7Q8G and 7Q8O.
Supplementary Table 13. Data collection and refinement statistics**:** PDB entries 7Q8J and 7QHK.
Supplementary Table 14. Data collection and refinement statistics**:** PDB entries 7Q8Q and 7QNS.
Supplementary Table 15. Data collection and refinement statistics**:** PDB entry 7QO2.
Supplementary Table 16. Peptide and protein cleavages.
Supplementary Table 17. Peptide and protein cleavages and predictions.

Supplementary Data 1. Input data – individual data sets of peptides for cathepsins K, V, B, L, S, and F.
Supplementary Data 2. Specific 941 cleavages – only one cleavage in the whole protein.
Supplementary Data 3. SVM models for predictions which can be used as input for PCSS server (https://salilab.org).
Supplementary Data 4. Peptide fragment identification.

Supplementary Note 1. Comparison of peptide and protein cleavages by cathepsins V, L and K

Supplementary References

## Supplementary figures and tables

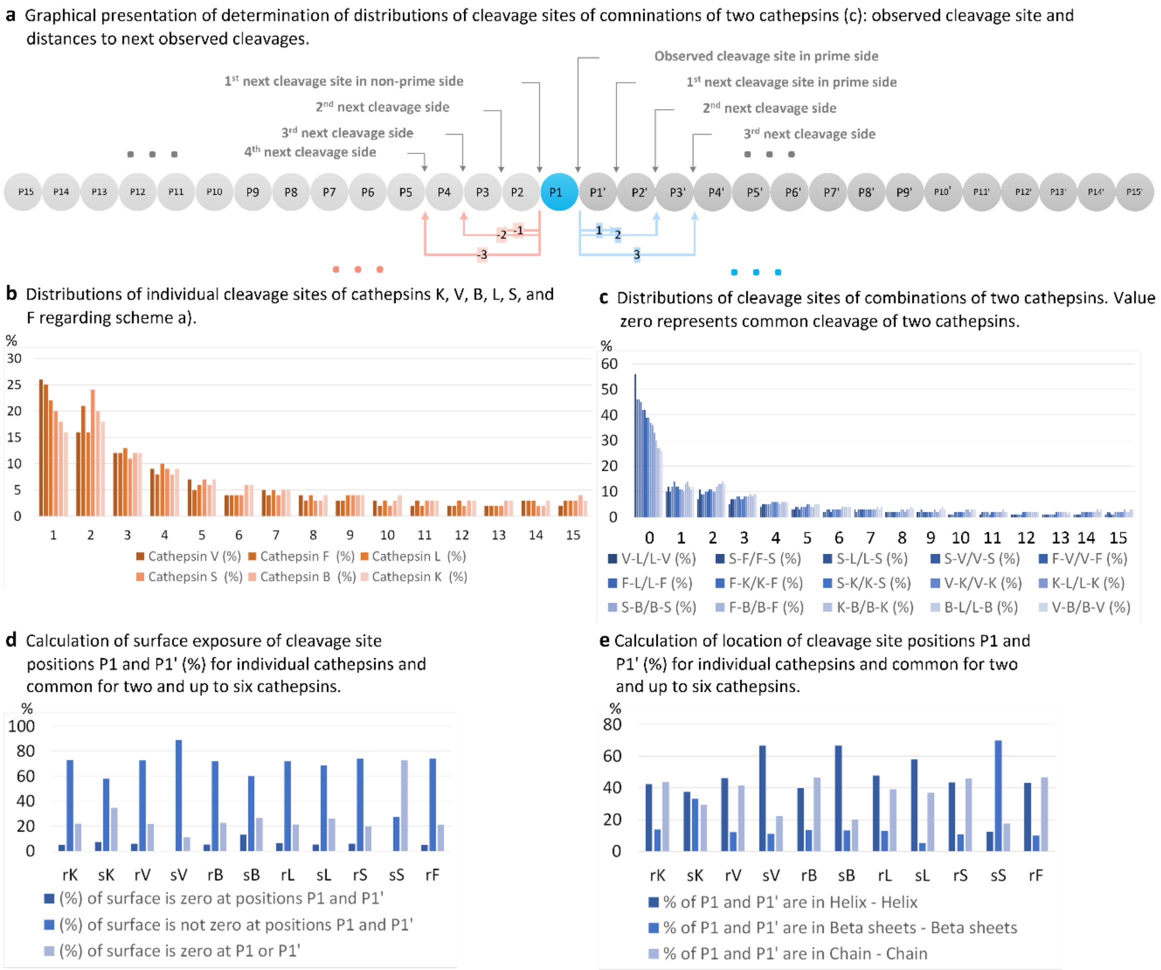


Common (shared) cleavage sites of cathepsins K, V, B, L, S, and F are represented as rK, rV, rB, rL, rS, and rF, respectively. Specific (unique and single) cleavage sites of cathepsins K, V, B, L, S, and F are represented as sK, sV, sB, sL, sS, and sF, respectively.

**Supplementary Fig. 1. Cleavage site separations and cleavage locations.**

**a.** Graphical presentation of determination of neighboring cleavages of one selected cleavage.

**b.** Cleavage site separations of individual cathepsins. The column diagram presents the share of neighborhood cleavage sites within the region of P15 to P15'.

**c.** Cleavage site separations of combinations of two cathepsins. The column diagram presents the shares of cleavages of the second cathepsin in the pair in the region of P15 to P15'.

**d.** The percentage of exposed surface area.

**e.** The percentage of cleavages with respect to the secondary structure.

These cleavage sites were analyzed for their solvent accessibility and secondary structure position (d and e) using MAIN[23]. The figures were generated with Excel.

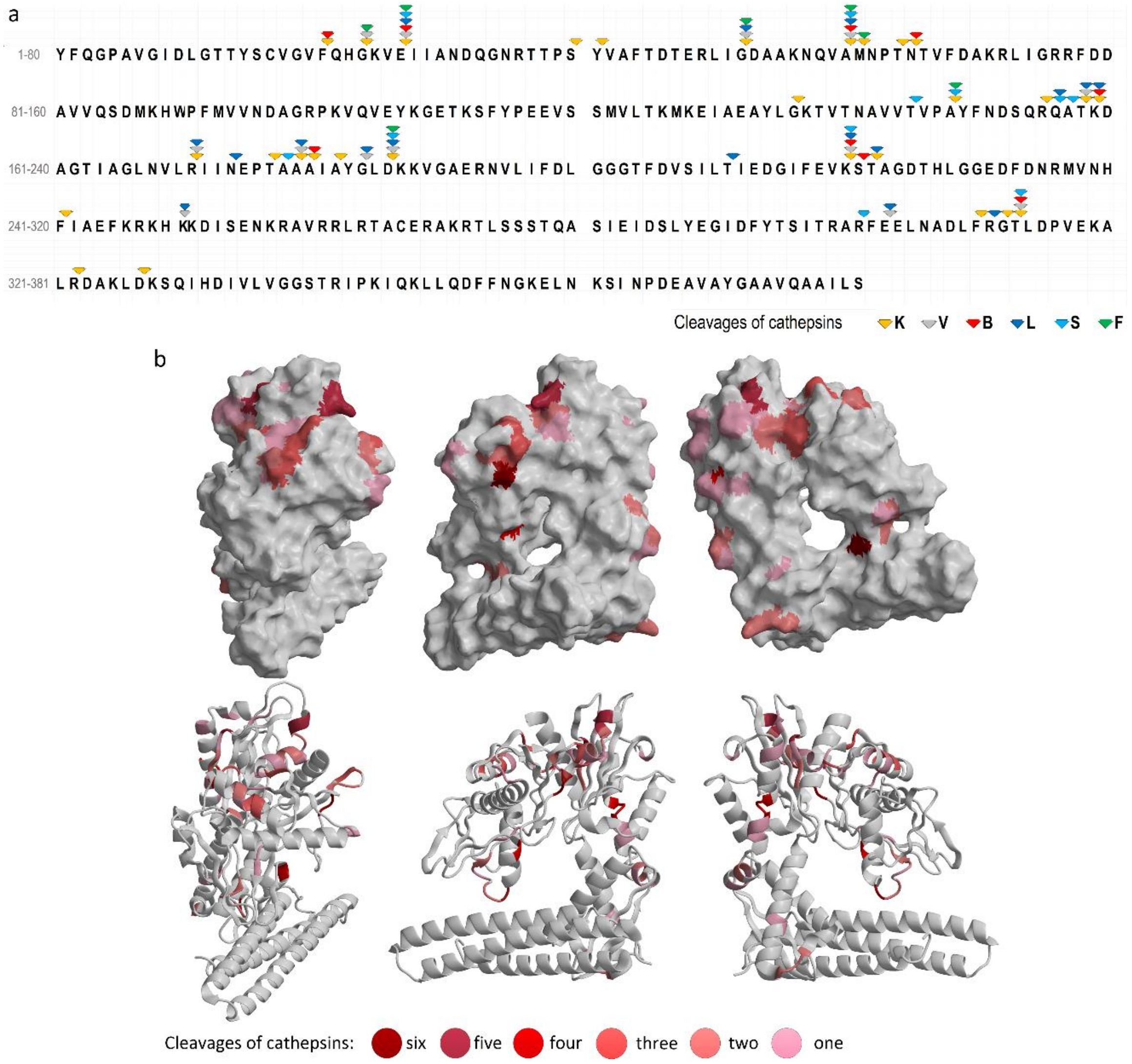


**Supplementary Fig. 2. Protein with unique and shared cleavage sites.**

**a.** Unique and shared cleavages mapped on the sequence. There were 41 cleavage sites in chain A and none in chain B in the 71 kDa heat shock "cognate" protein (PDB code 3LDQ and UniProt code P11142)[26].

**b.** Unique and shared cleavage cases mapped on the 3D structure. Three views of the structure of the nucleotide binding domain of the 72 kDa heat shock "cognate" are shown. Protein structures are shown in surface and ribbon presentations. Color coding shows the cleavage site residues P1 and P1', which are colored in 6 shades of red. The color shade corresponds to the number of cathepsins that performed the cleavage. Pale red corresponds to cleavage by one cathepsin, whereas the dark red areas were cleaved by all six cathepsins. The remainder of the structure is colored white.

The figures were generated with MAIN[23] and rendered with RASTER 3D[35].

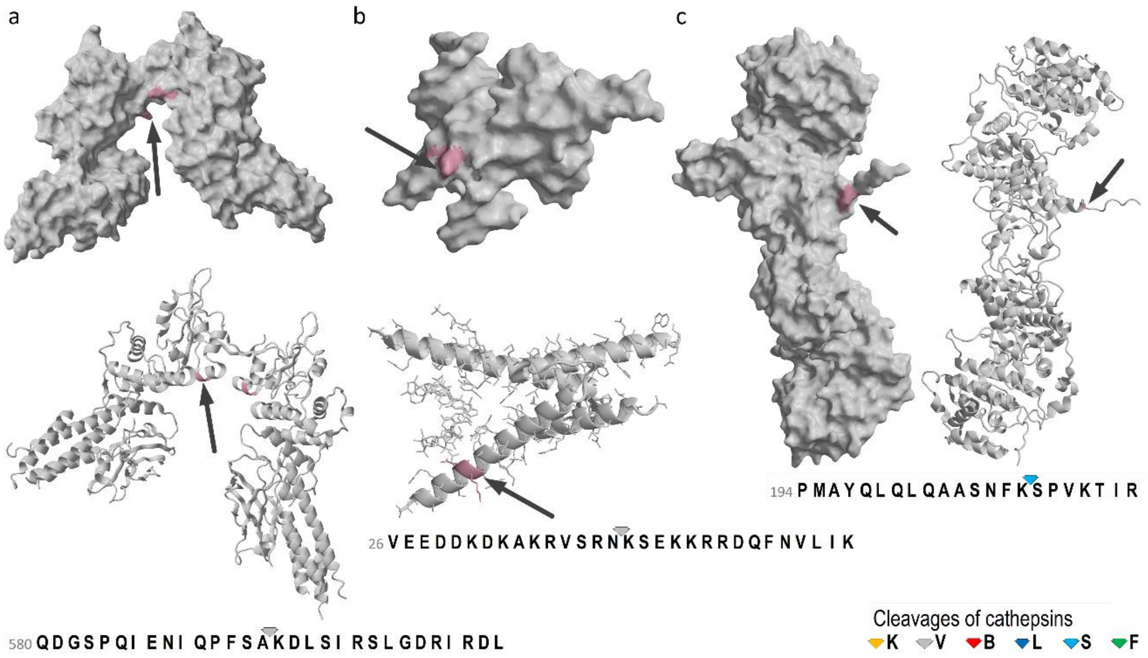


**Supplementary Fig. 3. Single cleavage cases mapped on the 3D structures.** The structure presentation and color coding are the same as in Fig. 1. The figures were generated with MAIN[23] and rendered with RASTER 3D[35].

**a.** Homodimer of the signal transducer and activator of the transcription 6 core fragment, namely, STAT6$^{CF}$ (PDB code 4Y5U and UniProt code P42226)[68], which was cleaved by cathepsin V between A594 and K595 within the sequence string PFSA↑KDLS of STAT6. The phosphorylated dimer of the STAT6 core fragment binds DNA within the cleaved region. This cleavage likely disrupted the dimeric structure of STAT6 and may have prevented the binding of DNA and, consequently, its transcription. The site is marked with a black arrow.

**b.** Human *c*ircadian locomotor output cycle kaput and brain and muscle ARNT-like 1 CLOCK-BMAL1 domain structures with E-box DNA (PDB code 4H10 and UniProt code O15516)[69], which was cleaved by cathepsin V between N40 and K41 within the sequence string VSRN↑KSEK in the CLOCK domain. This cleavage likely disrupted the structure of the DNA binding site and hints at either a regulative or disruptive role of cathepsin V in transcription. The site is marked with a black arrow.

**c.** β subunit of protein kinase CK2 (PDB code 4DGL and UniProt code P67870)[70], which was cleaved by cathepsin S between K208 and S209 in the middle of the SNFK↑SPVKT region, which contains three phosphorylation sites, among which S209 was shown to be related to enhancement of CK2 kinase activity in prostate cancer cells. The site is marked with a black arrow.

**a**

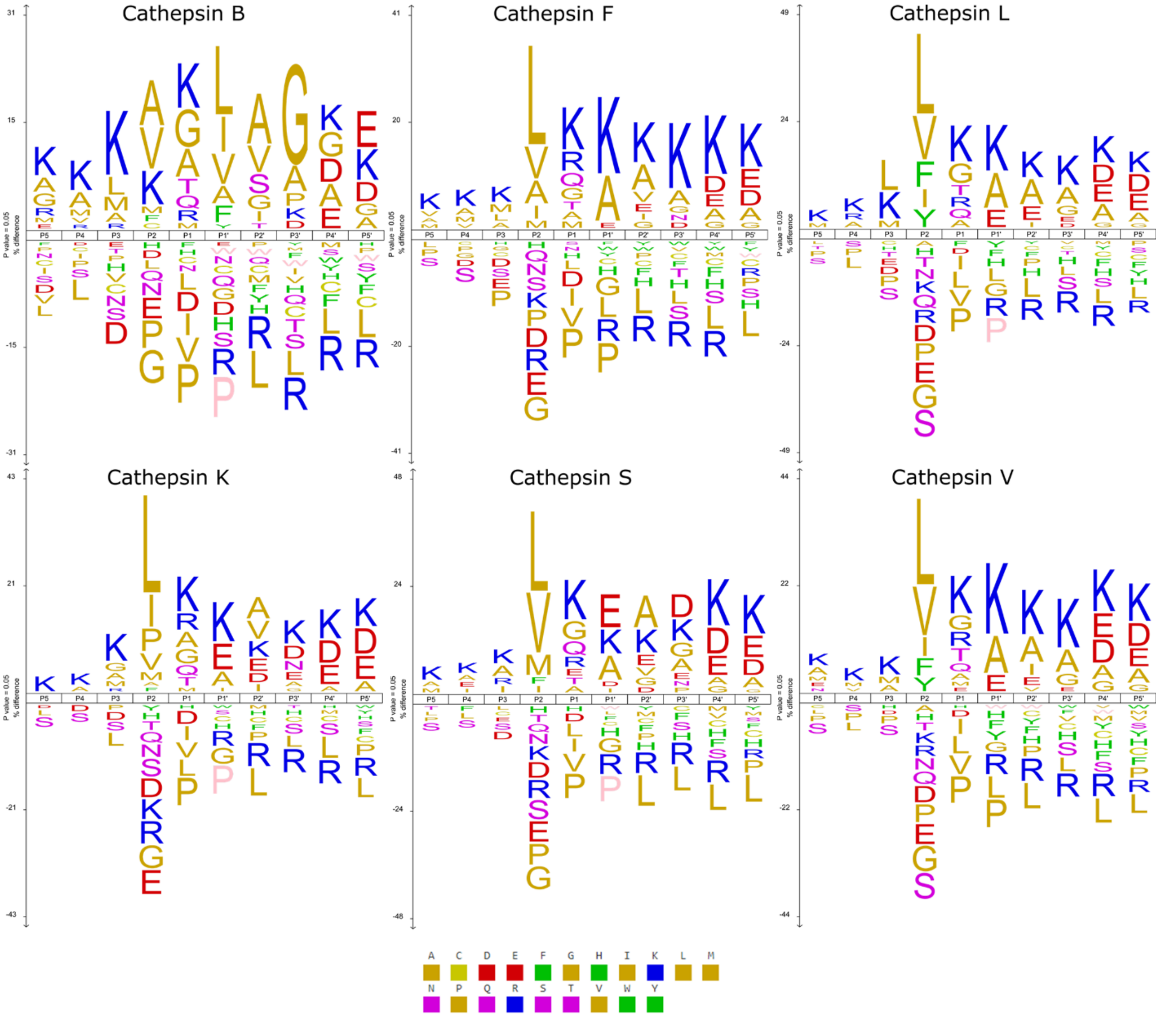
Cathepsin B
Cathepsin F
Cathepsin L
Cathepsin K
Cathepsin S
Cathepsin V
P5 P4 P3 P2 P1 P1' P2' P3' P4' P5'
P value = 0.05
% difference
A C D E F G H I K L M
N P Q R S T V W Y

**b**

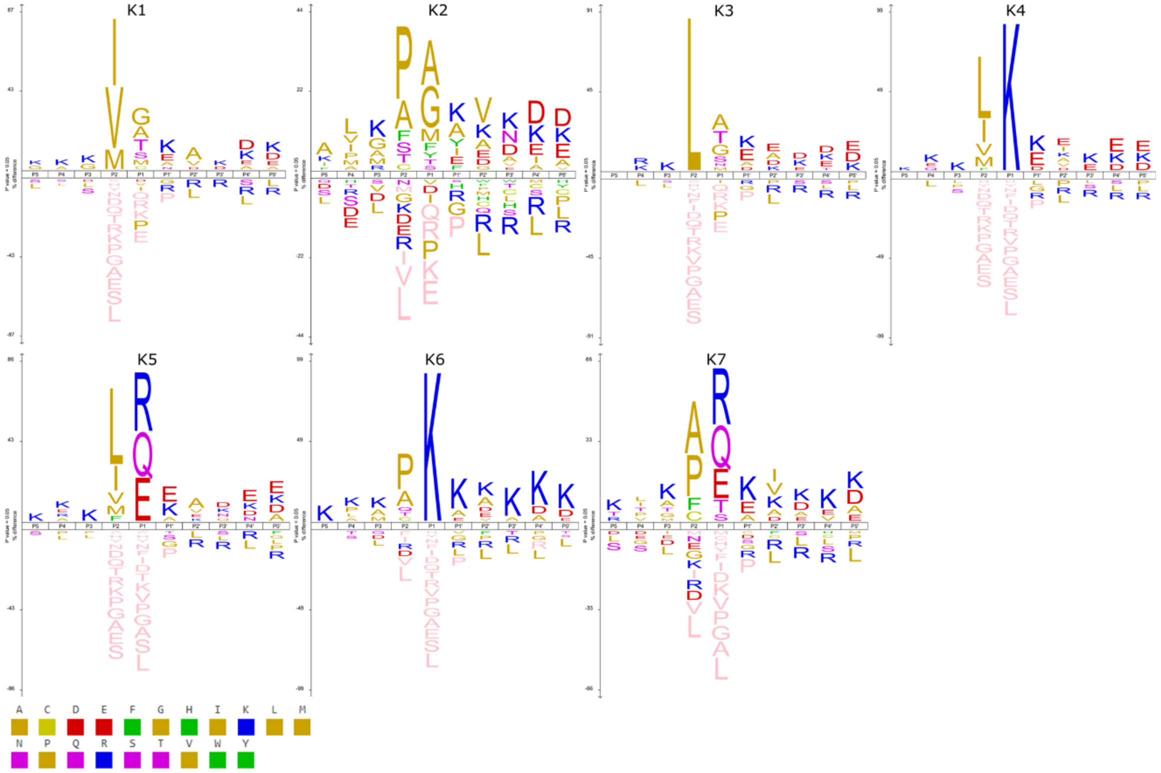
K1
K2
K3
K4
K5
K6
K7
A C D E F G H I K L M
N P Q R S T V W Y

**c**

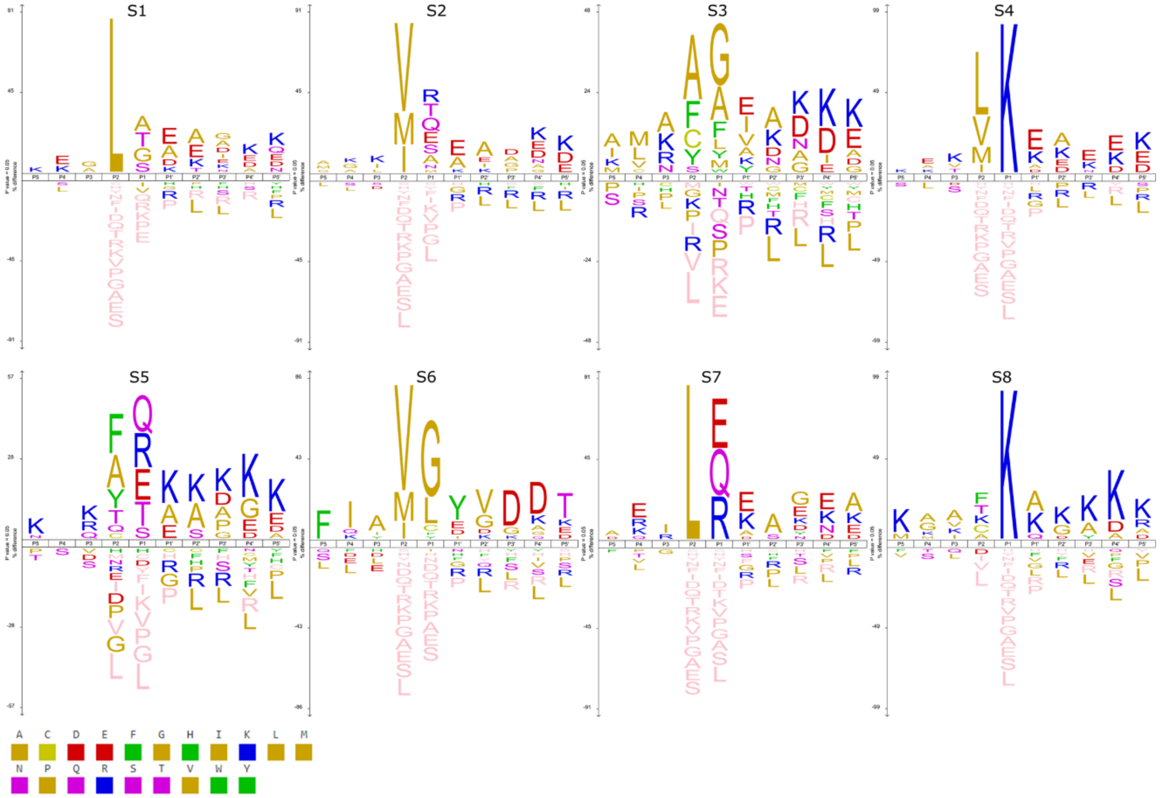
S1
S2
S3
S4
S5
S6
S7
S8
A C D E F G H I K L M
N P Q R S T V W Y

**d**

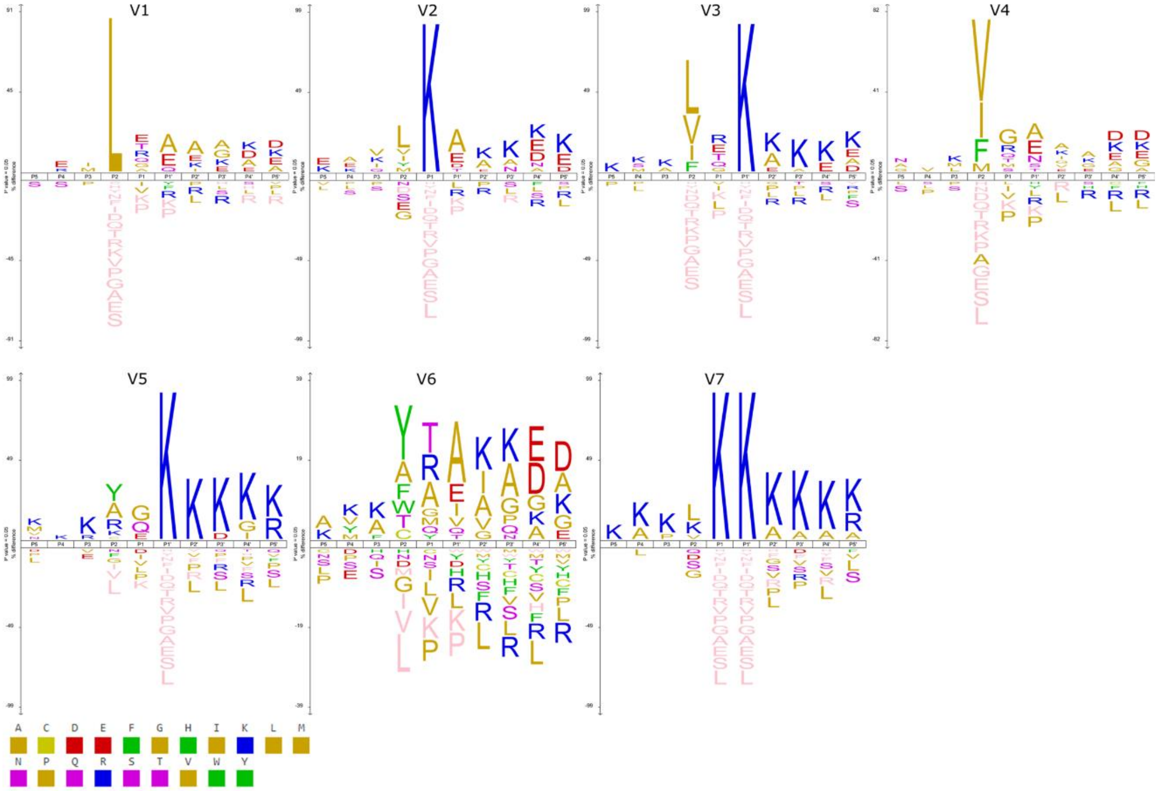
V1
V2
V3
V4
V5
V6
V7
A C D E F G H I K L M
N P Q R S T V W Y

**e**

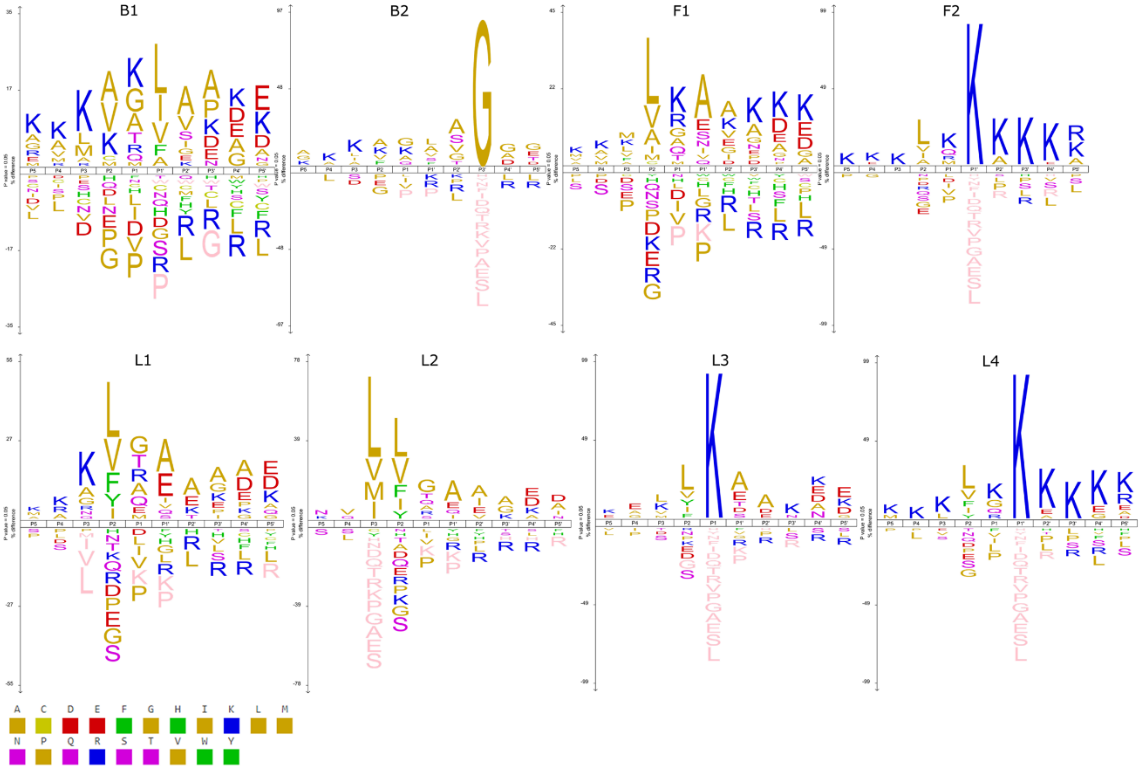
B1
B2
F1
F2
L1
L2
L3
L4
A C D E F G H I K L M
N P Q R S T V W Y

**Supplementary Fig. 4. iceLogo plots of datasets of substrates of cathepsins K, V, B, L, S, and V (a) and its belonging 30 clusters (b, c, d, e).** The clusters of substrates of cathepsins K are marked as K1, K2, K3, K4, K5, K6, and K7 (**b**); of cathepsin S as S1, S2, S3, S4, S5, S6, S7 and S8 (**c**); of cathepsin V as V1, V2, V3, V4, V5, V6, and V7 (**d**); of cathepsin B as B1 and B2, of cathepsin F as F1 and F2, and of cathepsin L as L1, L2, L3, and L4 (**e**)[14].

The plots visualize significantly different residue frequencies (above the abscissa) and significantly under represented residues (below the abscissa). The character size corresponds to the percentage of difference from the average amino acid occurrence. P value was set to 0.05.

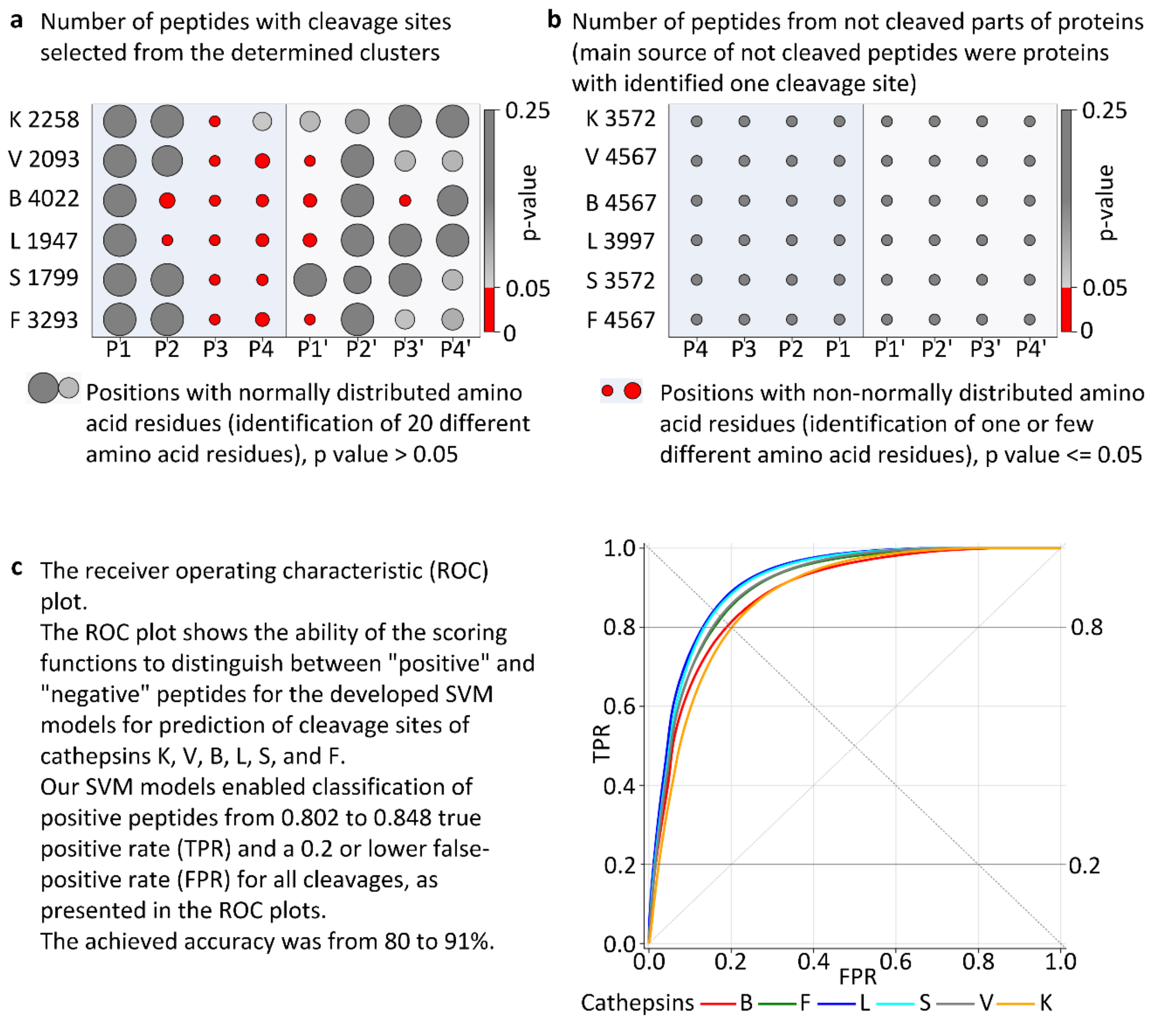


**Supplementary Fig. 5. The selection of training sets for developed SVM models** and achieved ability of prediction of cleavage sites for substrates of cathepsins K, V, B, L, S, and F on the basis of presented receiver operating characteristic (ROC) plot (the plot was calculated by using PCSS server[16]).

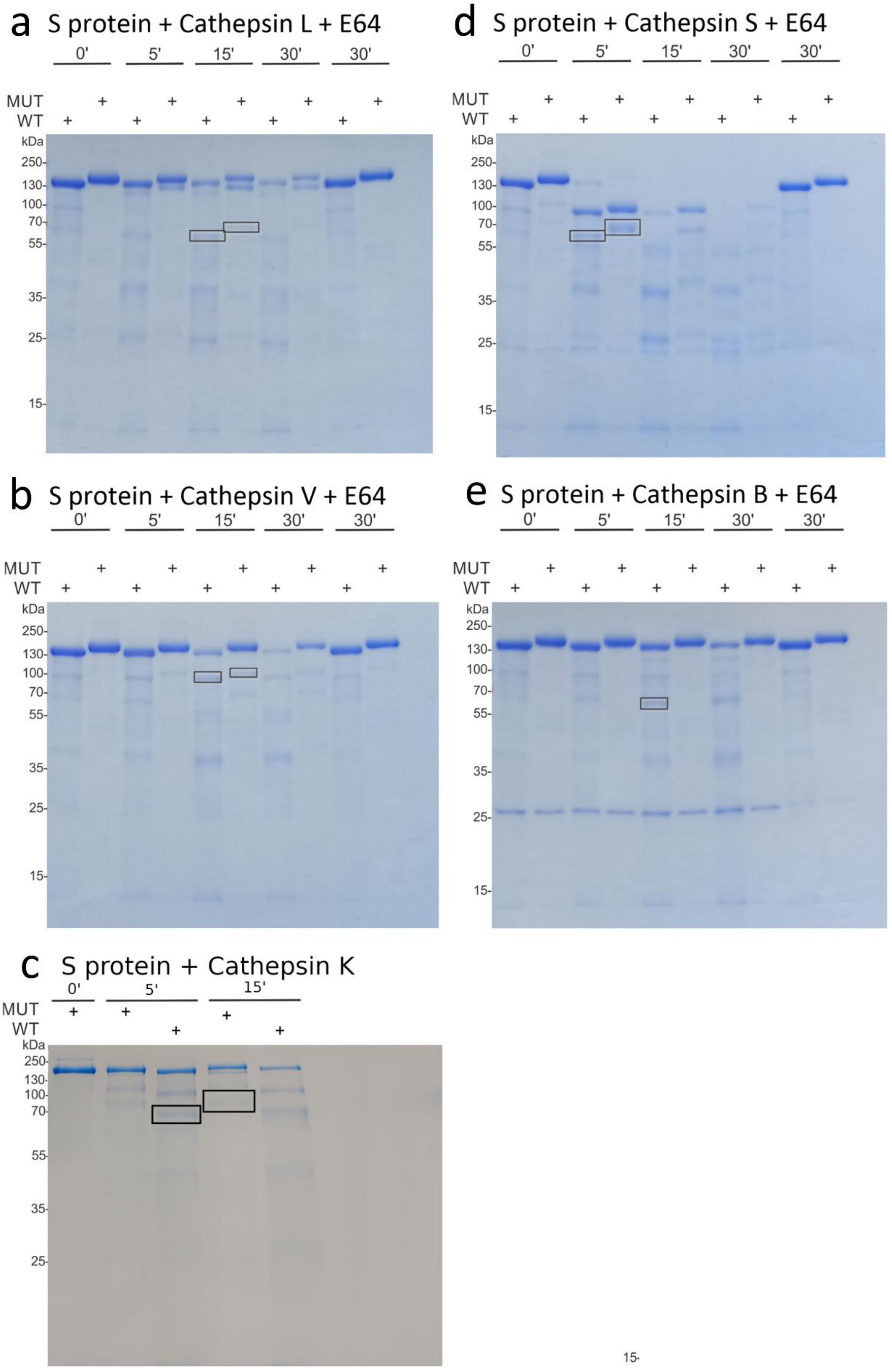


Selected samples for N terminal sequence analysis

**Supplementary Fig. 6. SDS page analysis of SARS-CoV-2 S protein degradation/processing; Cleavages of SARS-CoV-2 wild-type (WT) and mutated furin cleavage site (MUT) S proteins.**

**a.** SDS–PAGE analysis of the processing of WT and MUT S proteins by cathepsins L,
**b.** Cathepsin V,
**c.** Cathepsin K,
**d.** Cathepsin S, and
**e.** Cathepsin B are presented.

WT and MUT were loaded in air on SDS–PAGE columns at 0, 5, 15 or 30 min after incubation with cathepsins. The control samples of S protein (WT and MUT) contain in addition cathepsin inhibitor E-64 (second number 30’ in panels a, b, d, and e). Assuming that the first degrading product corresponds to the S protein N-terminal fraction, the second fragments were analyzed by N-terminal sequencing (analyzed samples are marked with black boxes).

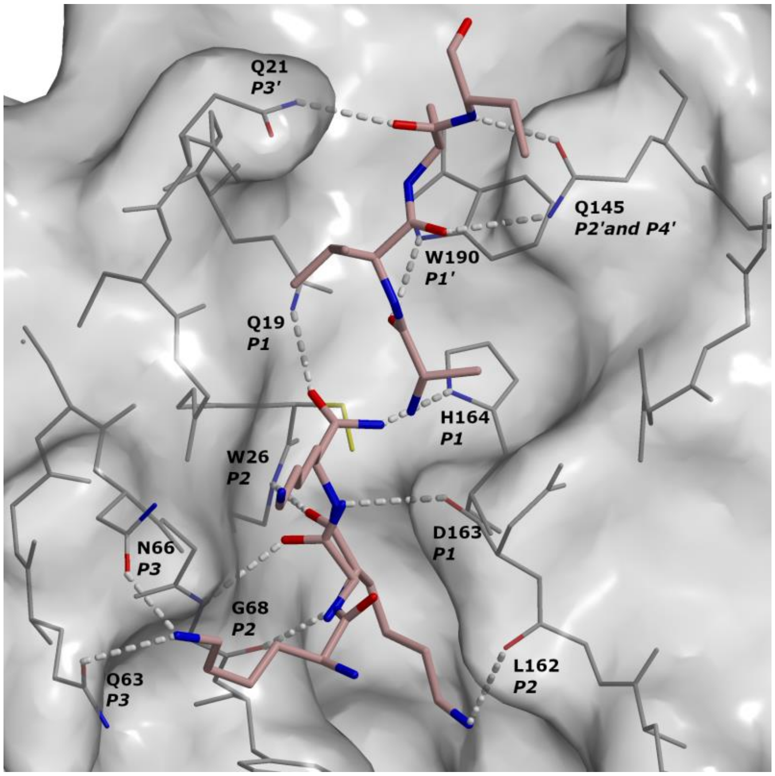


**Supplementary Fig. 7. H-bonding pattern between cathepsin V and substrates.** Peptide fragments KKK (P3–P1) and AVAE (P1'–P4') are shown on the surface of a semi-transparent cathepsin V structure. Interacting oxygen, nitrogen, and carbon atoms of peptides and cathepsin are shown in red, blue, rose and gray, respectively. H-bonds are presented as white dashed lines. At position P2, two main chain conformations are presented. In one conformation, the H-bond is formed between the O of P2 and the N of W26, and in the other, the O of P2 forms H-bond with the N of G68. Cathepsin residues that participated in peptide H-bonding are marked with sequence IDs and the peptide position. The mutant of catalytic residue S25 is highlighted in yellow. Figures in the panel were prepared using MAIN[23] and rendered using Raster 3D[35].

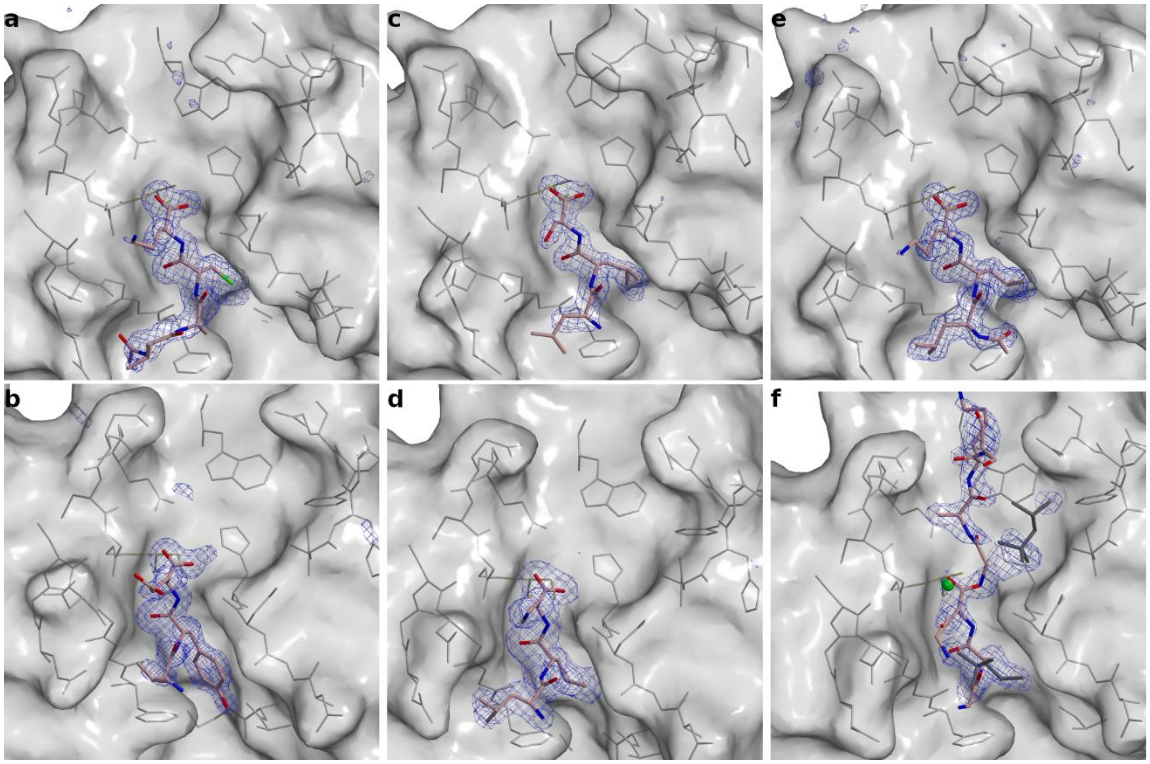


**Supplementary Fig. 8. Electron density maps of peptides in the group of pattern I.** Peptide nitrogen atoms are shown in blue, oxygen atoms in red, and carbon atoms in pale pink.

**a.** Fragment VACK of peptide VACKSSQP (structure 7QFF).
**b.** Fragment VYE of peptide VYEKKP (structure 7QNS).
**c.** Fragment LLS of peptide LLSGKE (structure 7Q8O).
**d.** Fragment LLK of peptide LLKVAL (structure 7Q8K).
**e.** Fragment LLK of peptide LLKAVAEKQ (structure 7Q9H).
**f.** Peptide GAK of peptide GAKSAA (structure 7QO2) is shown in the non-primed site. Primed site is occupied by peptide GAKSAA, belonging to a group of pattern IV.

Electron densities were constructed using free kick omit Fo–Fc map[39]. Figures in the panel were prepared using MAIN[23] and rendered using Raster 3D[35].

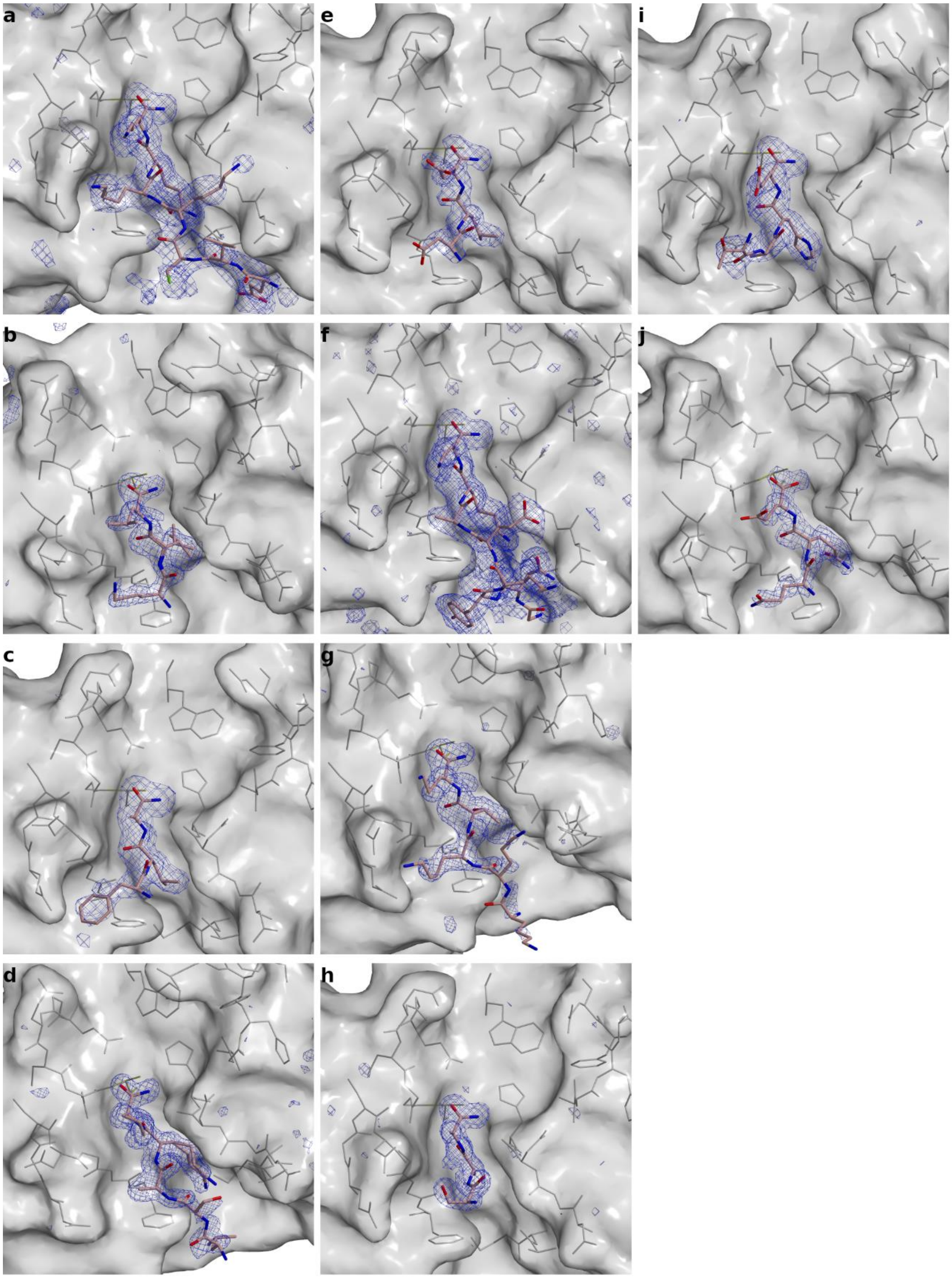
a
b
c
d
e
f
g
h
i
j

**Supplementary Fig. 9. Electron density maps of peptides in the group of pattern II.** Peptide nitrogen atoms are shown in blue, oxygen atoms in red, and carbon atoms in pale pink.

**a.** Peptide EVCKKKK (structure 7Q8H).
**b.** Fragment KVL of peptide AYFKKVL (structure 7QFH).
**c.** Fragment FLA of peptide KKYDAFLA (structure 7Q8N).
**d.** Fragment LSAKP of peptide RLSAKP (protected) (structure 7Q9C).
**e.** Fragment DLE of peptide TRESEDLE (structure 7Q8D).
**f.** Peptide GNYKEAKK (structure 7Q8F).
**g.** Fragment KKKTK peptide KPKKKTK (structure 7Q8M).
**h.** Fragment SAA of peptide GAKSAA (structure 7QHJ).
**i.** Fragment TAHE of peptide VPCGTAHE (structure 7Q8L).
**j.** Fragment QQE of peptide QLRQQE (structure 7QHK).

Electron densities were constructed using free kick omit Fo–Fc map[39]. Figures in the panel were prepared using MAIN[23] and rendered using Raster 3D[35].

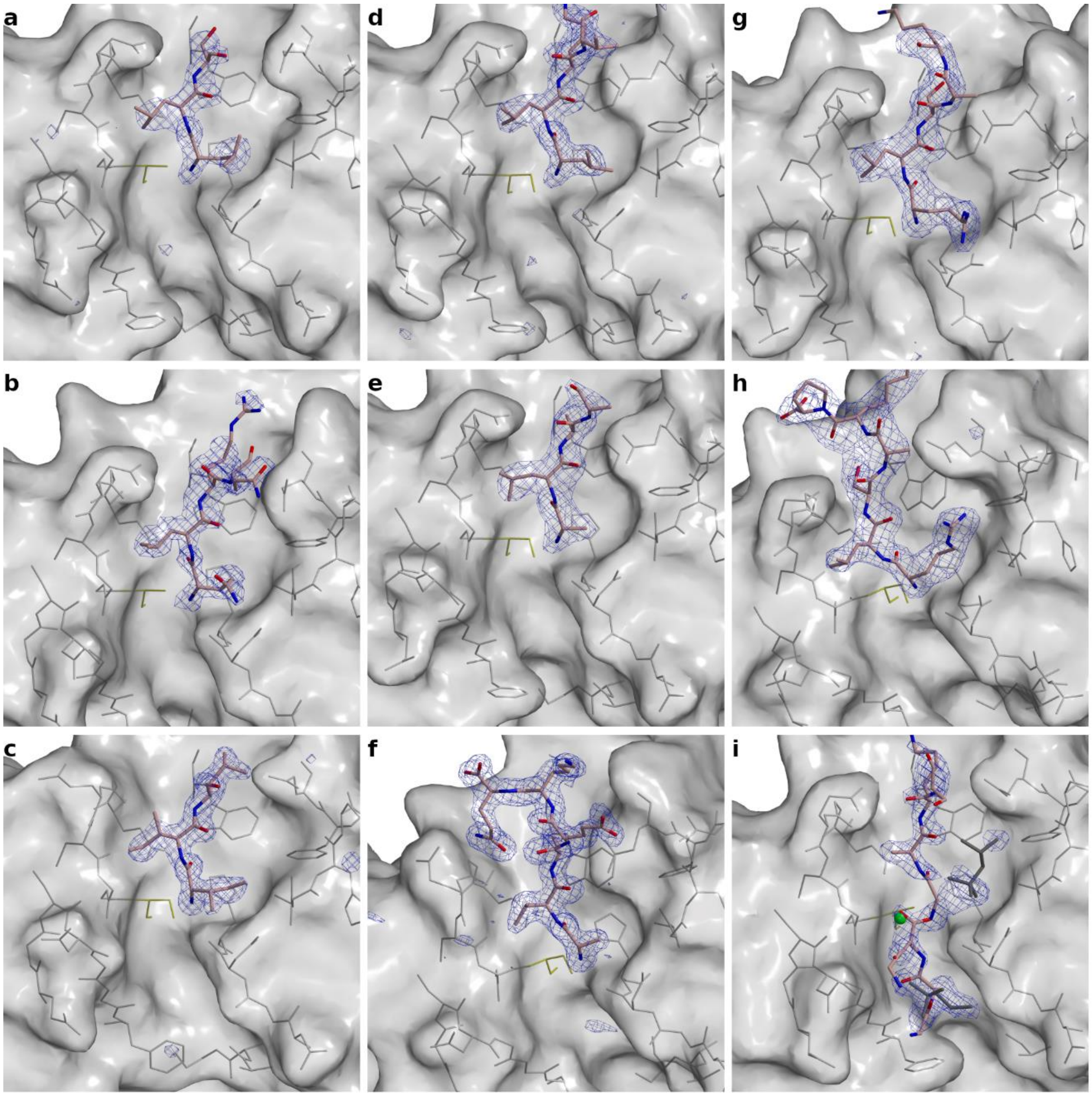


**Supplementary Fig. 10. Electron density maps of peptides in the group of pattern III.** Peptide nitrogen atoms are shown in blue, oxygen atoms in red, and carbon atoms in pale pink.

**a.** Fragment LLS of peptide LLSGKE (structure 7Q8O).
**b.** Fragment QLRQ of peptide QLRQQE (structure 7QHK).
**c.** Fragment IIL of peptide IILKEK (structure 7Q8J).
**d.** Fragment LLKV of peptide LLKVAL (structure 7Q8P).
**e.** Fragment ALAA of peptide ALAASS (structure 7Q8G).
**f.** Peptide AVAEKQ (structure 7Q8I).
**g.** Fragment RLSAK of peptide RLSAKP (non-protected; molecule A) (structure 7Q8Q).
**h.** Peptide RLSAKP (non-protected; molecule B) (structure 7Q8Q).
**i.** Fragment GAKS of peptide GAKSAA is shown in the primed site (structure 7QO2).

Non-primed site is occupied by peptide GAKSAA, belonging to group of pattern I. Electron densities were constructed using free kick omit Fo–Fc map[39]. Figures in the panel were prepared using MAIN[23] and rendered using Raster 3D[35].

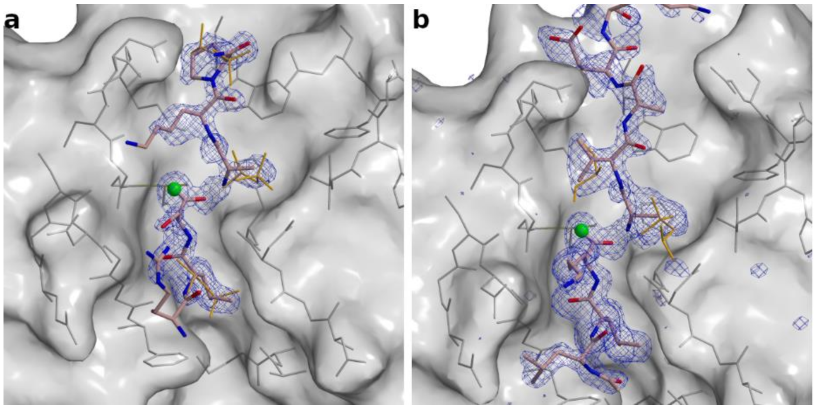


**Supplementary Fig. 11. Electron density maps of peptides in the group of pattern IV.** Peptide nitrogen atoms are shown in blue, oxygen atoms in red, and carbon atoms in pale pink.

**a.** Fragments RLS and AKP of peptide RLSAKP (structure 7Q9C).

**b.** Fragments LLK and AVAEKQ of peptide LLKAVAEKQ (structure 7Q9H).

Electron densities were constructed using free kick omit Fo–Fc map[39]. Figures in the panel were prepared using MAIN[23] and rendered using Raster 3D[35].

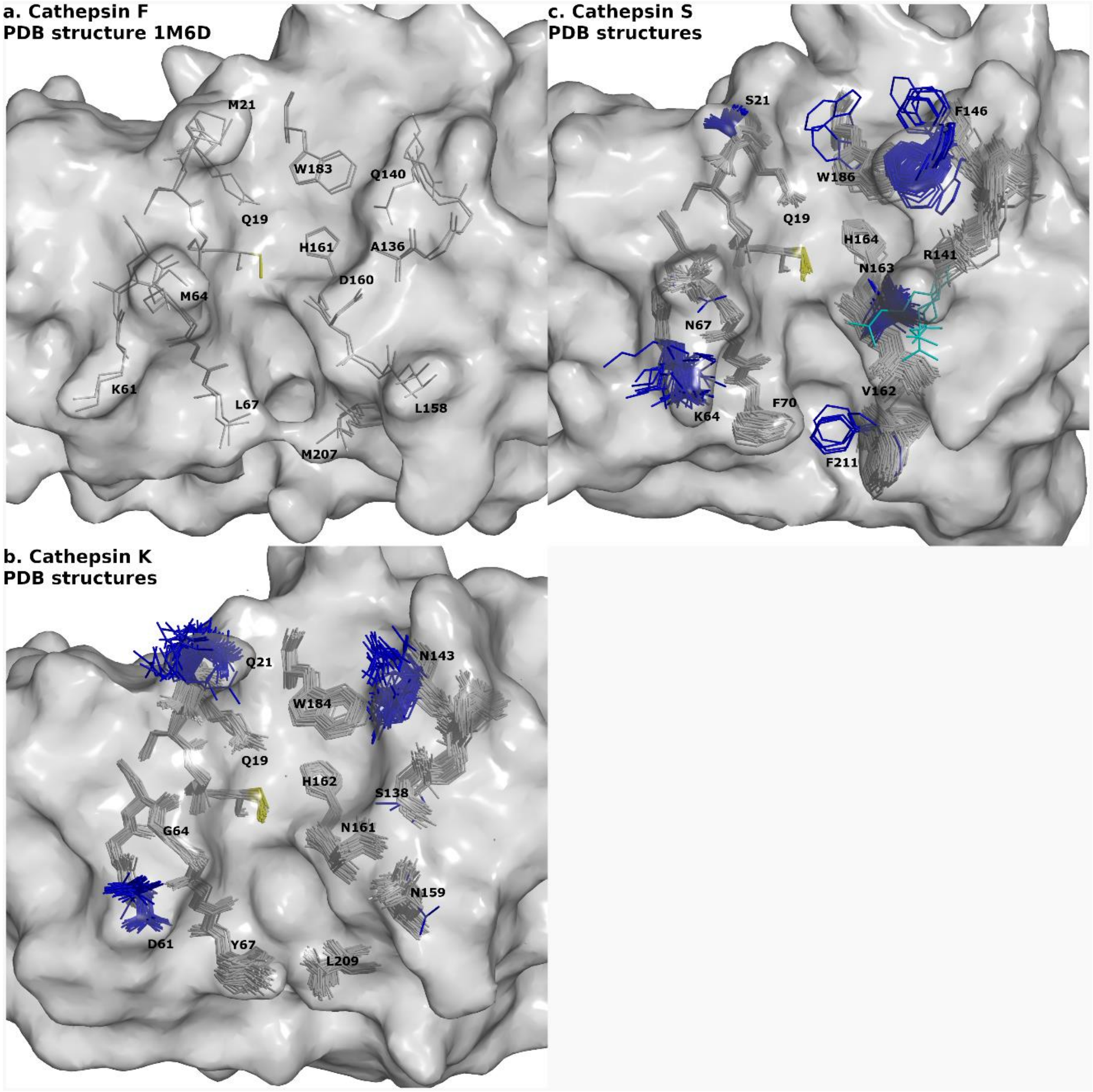


**Supplementary Fig. 12. Flexible and rigid residues of cathepsins K, S and F from PDB database.** Surfaces of cathepsins are colored gray. Cathepsin residues are presented with bond models. Flexible residues around the active site are highlighted in blue. Catalytic residues at site 25 are shown in yellow.

**a.** Cathepsin F (PDB entry *1M6D*).

**b.** Cathepsin K (*PDB entries 1BGO, 1NLJ, 1ATK, 1AU0, 1AU2, 1AU3, 1AU4, 1AYU, 1AYV, 1AYW, 1BY8, 1MEM, 1NL6, 1Q6K, 1SNK, 1TU6, 1U9V, 1U9W, 1U9X, 1YK7, 1YK8, 1YT7, 2ATO, 2AUX, 2AUZ, 2BDL, 2F7D, 2FTD, 2R6N, 3C9E, 3H7D, 3KW9, 3KWB, 3KWZ, 3KX1, 3O0U, 3O1G, 3OVZ, 4DMX, 4DMY, 4N79, 5N8W, 4X6H, 4X6I, 4X6J, 4YV8, 4YVA, 5J94, 5JA7, 5JH3, 5TDI, 5TUN, 5Z5O, 6ASH, 6HGY, 6PXF, 6QBS, 6QL8, 6QLM, 6QLW, 6QLX, 6QM0, 7NXL, 7NXM, 7PCK.*

**c.** Cathepsin S (*PDB entries 2HXZ, 2F1G, 2FT2, 3OVX, 2R9N, 2R9M, 1MS6, 2HHN, 4P6G, 2OP3, 4P6E, 6YYN, 2HH5, 2G7Y, 2FQ9, 6YYR, 6YYP, 2H7J, 2FRQ, 3N3G,*

*2R9O*, *2FRA*, *6YYO*, *3N4C*, *2FUD*, *1NPZ*, *1NQC*, *5QC0*, *5QCH*, *5QC4*, *5QC2*, *2C0Y*, *5QCG*, *5QCE*, *5QCI*, *5QCC*, *5QCA*, *5QC7*, *5QBV*, *5QC5*, *5QBZ*, *5QBX*, *5QC9*, *5QC3*, *5QC1*, *3IEJ*, *5QCF*, *5QCJ*, *5QCD*, *5QCB*, *5QBW*, *5QC8*, *5QC6*, *5QBU*, *2G6D*, *5QBY*, *1GLO*, *2FYE*. Only three residues of flexible R141 are presented in cyan (entries *2FUD* and *5QCA*) for clarity of the figure.

Figures in the panel were prepared using MAIN[23] and rendered using Raster 3D[35].

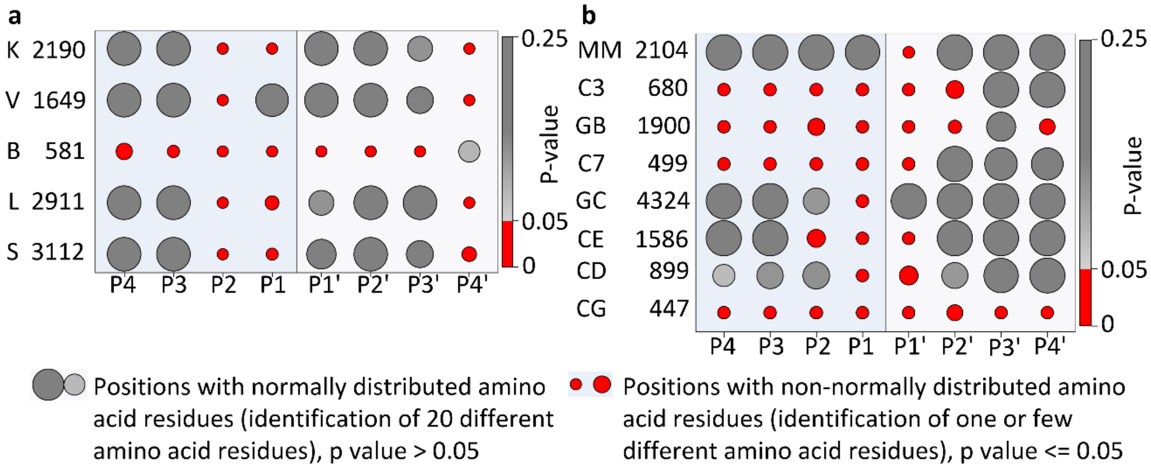


**Supplementary Fig. 13. Calculated heterogeneous and homogeneous positions of substrates for selected enzymes downloaded from MEROPS database**[40] **(https://www.ebi.ac.uk/merops/).** The substrate datasets for the following enzymes were used (the substrates without UniProt code were dropped out):

**a.** Cathepsins K (K), V (V), B (B), L (L), and S (S);

**b.** Peptidyl-Lys metallopeptidase (MM) which has on P1' only lysine, consequently p value was not calculated. To get a red spot we put p value equal to 0.0000000001; caspase-3 (C3); granzyme B (Homo sapiens-type) (GB); caspase-7 (C7); glutamyl endopeptidase I (GC); cathepsin E (CE); cathepsin D (CD); cathepsin G (CG).

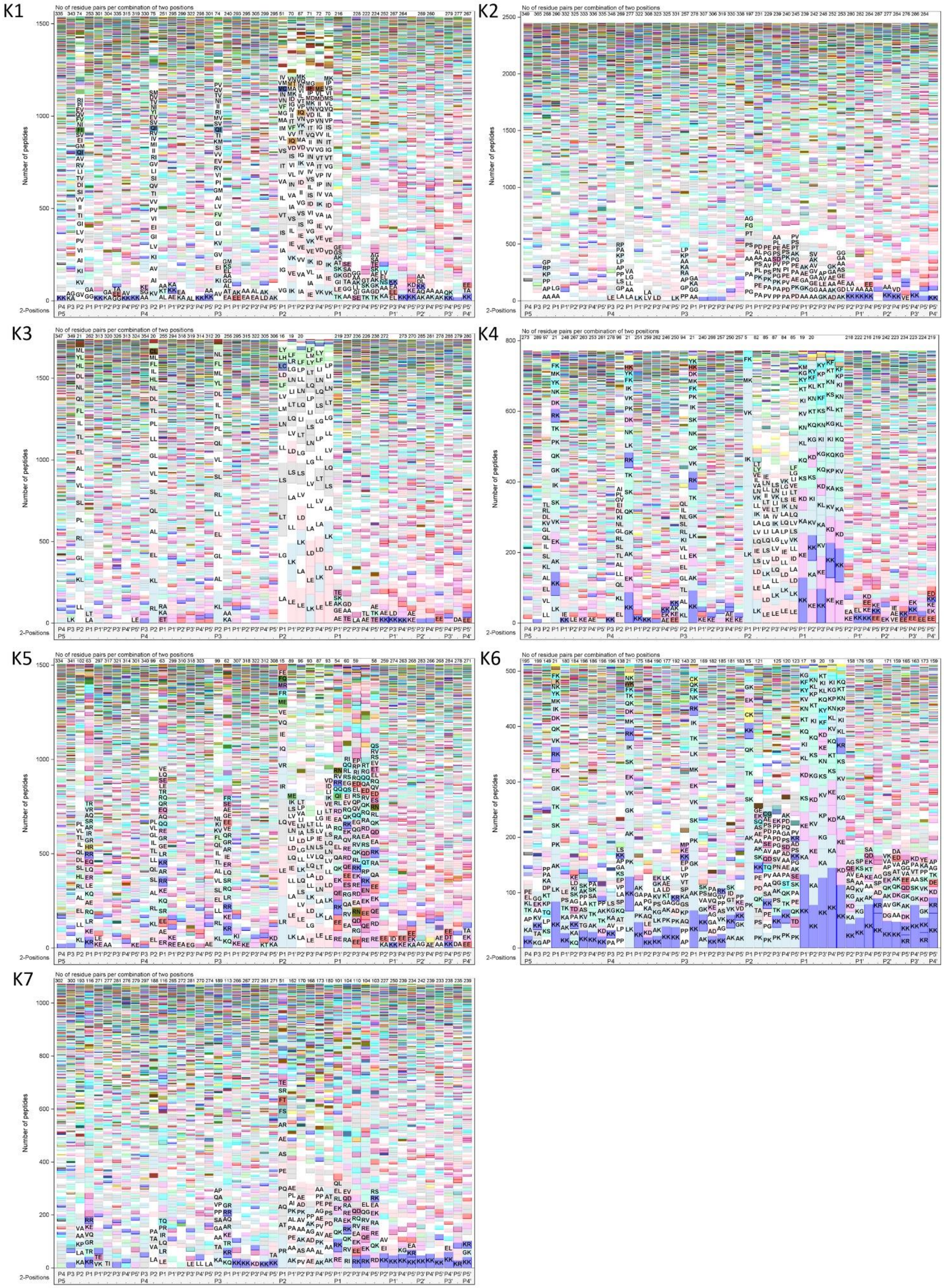
K1
K2
K3
K4
K5
K6
K7
No of residue pairs per combination of two positions
Number of peptides
2-Positions
P4 P3 P2 P1 P1' P2' P3' P4' P5'
P5 P4 P3 P2 P1 P1' P2' P3' P4'

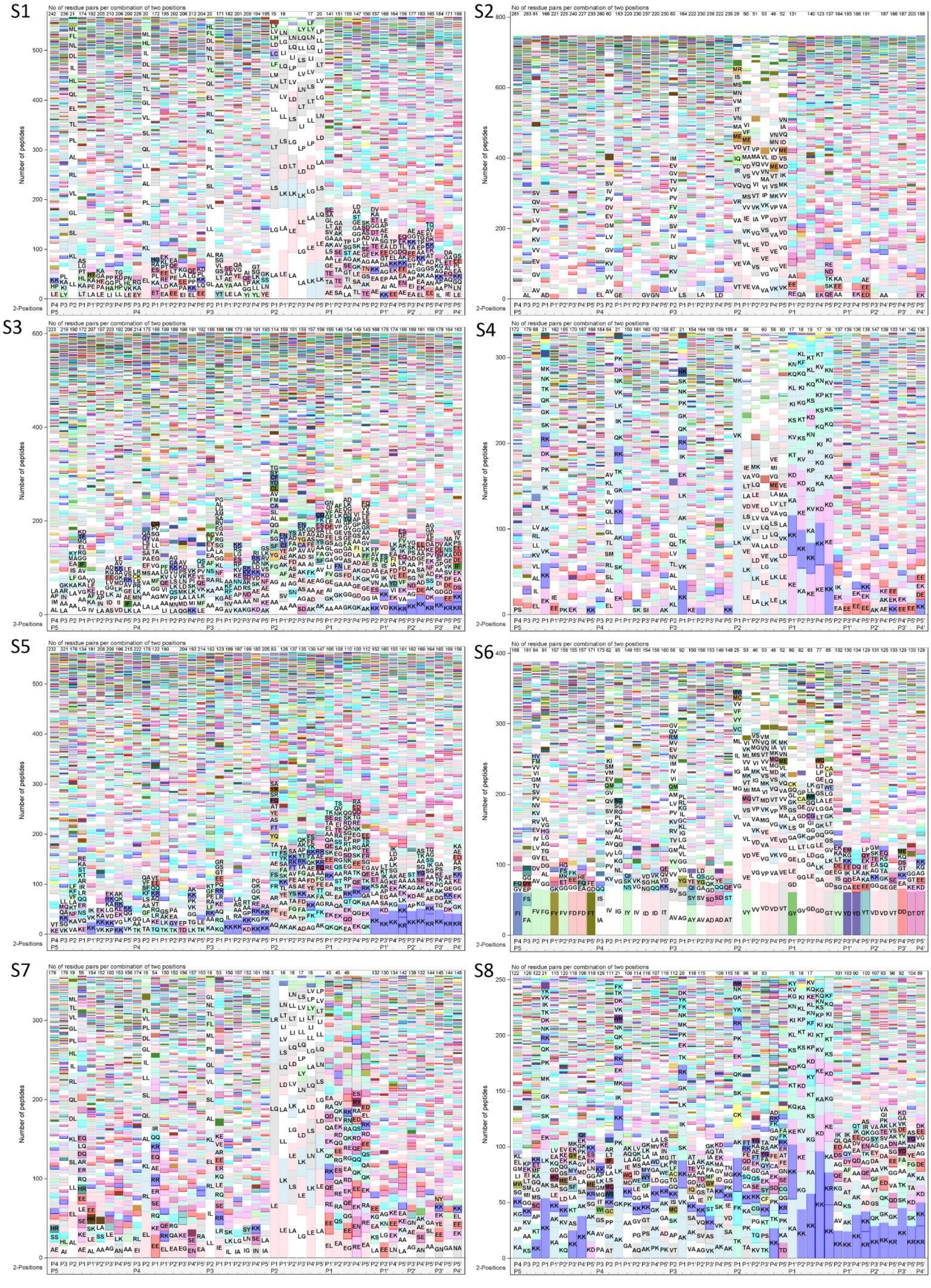
S1
S2
S3
S4
S5
S6
S7
S8
No of residue pairs per combination of two positions
Number of peptides
2-Positions
P4 P3 P2 P1 P1' P2' P3' P4' P5'
P5 P4 P3 P2 P1 P1' P2' P3' P4'

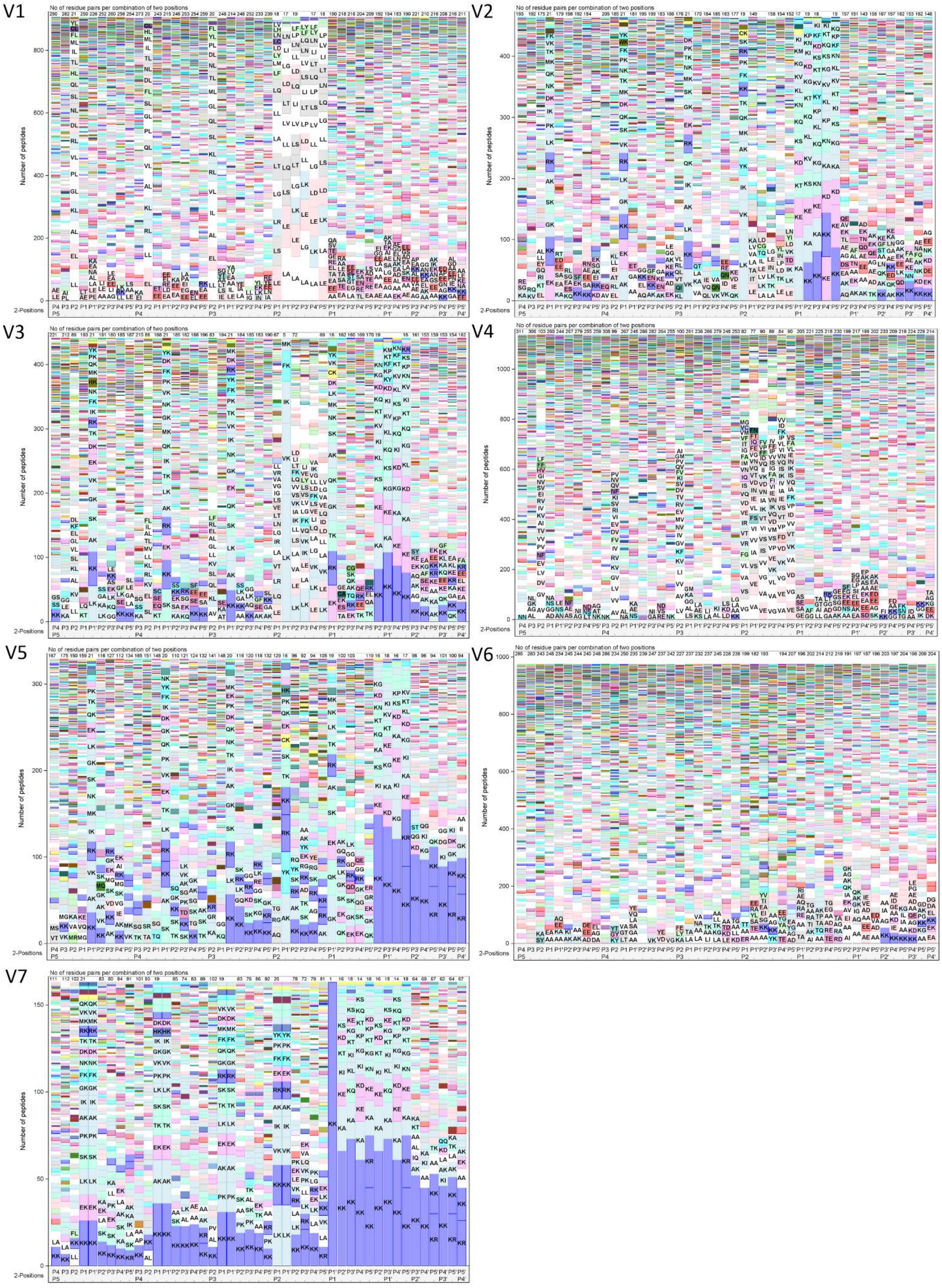
V1
V2
V3
V4
V5
V6
V7
No of residue pairs per combination of two positions
Number of peptides
2-Positions

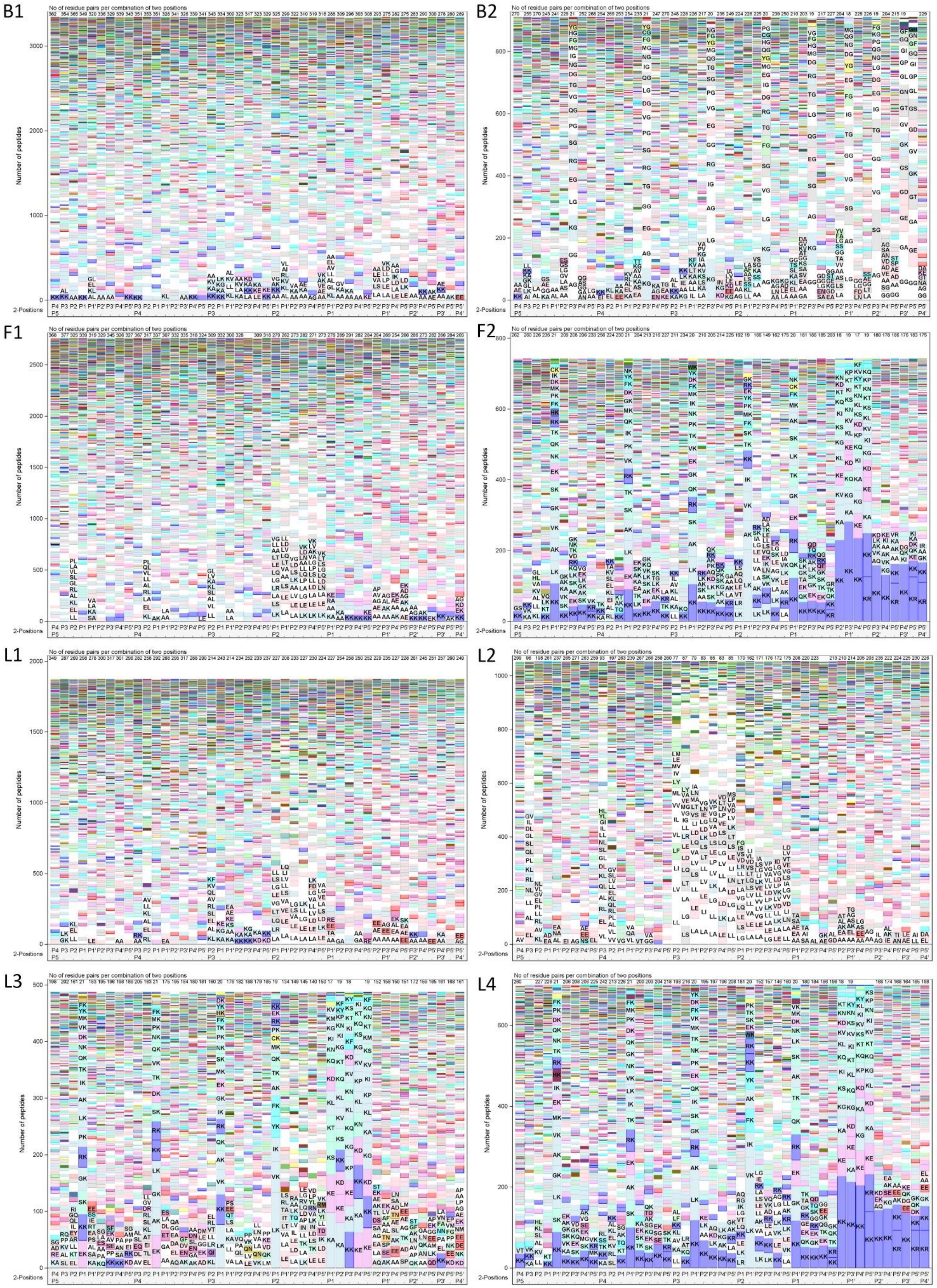
B1
B2
F1
F2
L1
L2
L3
L4
No of residue pairs per combination of two positions
Number of peptides
2-Positions

**Supplementary Fig. 14. Combinations of pair positions with pair residues for clusters of substrates of cathepsins K, S, V, B, F, and L.** Each cluster presents bars of residue pairs at combinations of two sites in the region from P5 to P5'. The number of pairs is specified at the top of each bar, whereas combinations of positions are presented at the bottom of each bar. Each bar is composed of blocks, where each block represents a specified combination of amino acid residues. The size of the block corresponds to the occurrence of the pair. For pairs that occurred in blocks that are large enough to include characters of font size 12, the amino acid pair codes are shown. The colors of the pairs represent the chemical characteristics of the amino acid residues, namely, hydrophobic NH (white), hydrophilic NE (cyan), negatively charged NC (red), positively charged PC (blue), aromatic hydrophobic AH (green) and cysteine C (yellow), as presented in the small 6x6 color scheme.

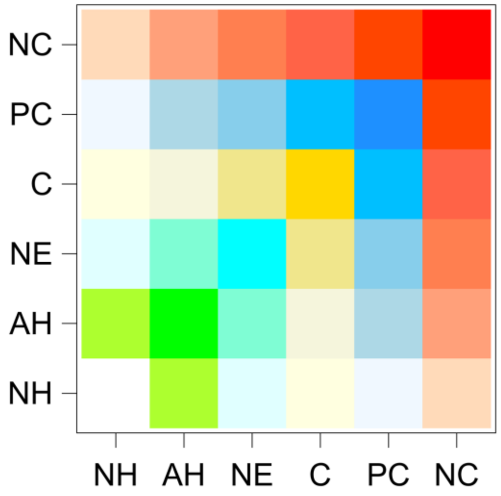


The clusters of substrates of cathepsins K are marked as K1, K2, K3, K4, K5, K6, and K7; of cathepsin S as S1, S2, S3, S4, S5, S6, S7 and S8; of cathepsin V as V1, V2, V3, V4, V5, V6, and V7; of cathepsin B as B1 and B2, of cathepsin F as F1 and F2, and of cathepsin L as L1, L2, L3, and L4.

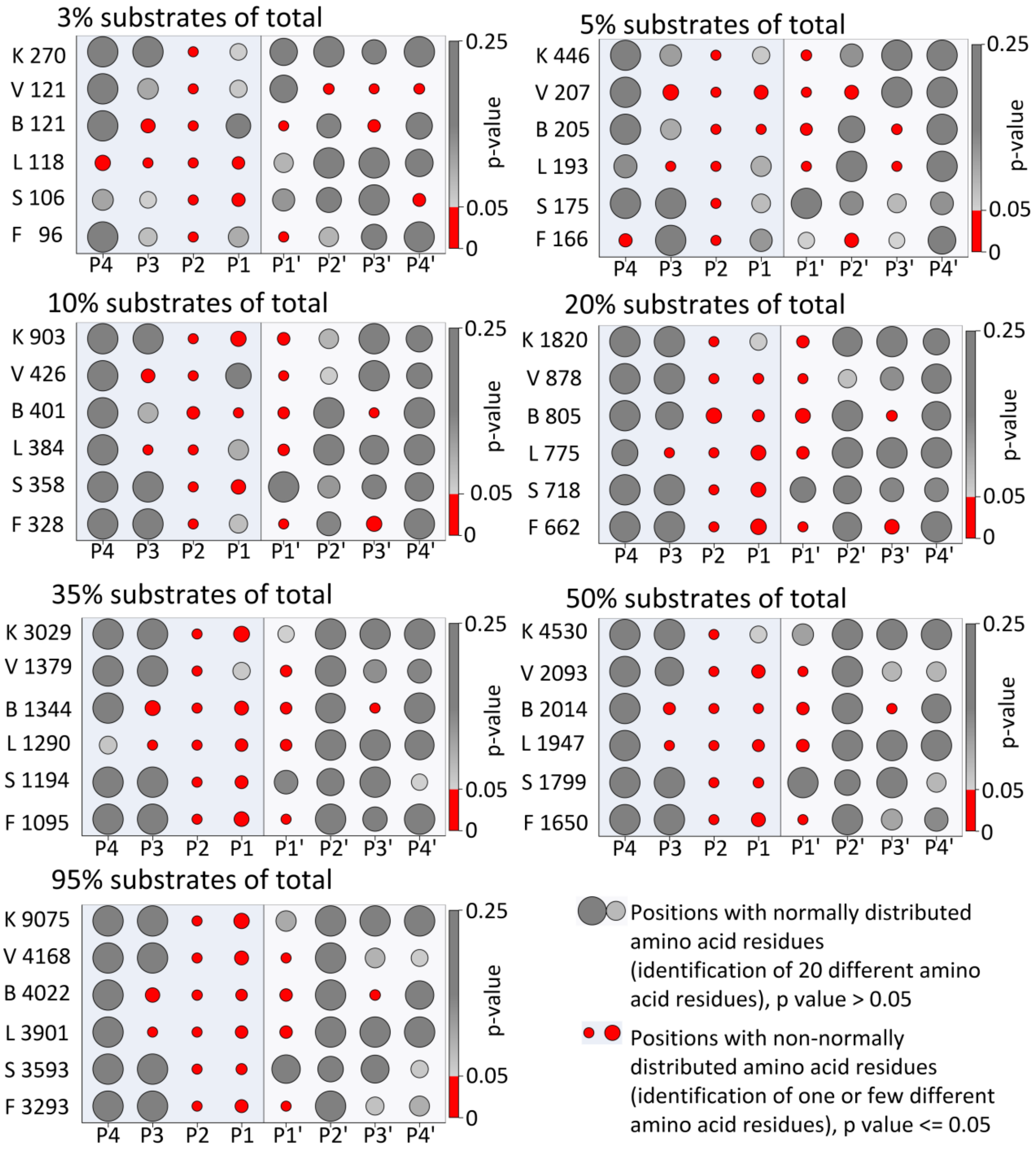


**Supplementary Fig. 15. Identification of heterogeneous and homogeneous positions depend on the number and relevancy of selected peptides.** To show the importance of the selection of the adequate number of peptides for the investigation of the cathepsins' specificity the different shares of peptides from datasets divided into clusters to ensure the diversity of peptides were selected: 3%, 5%, 10%, 20%, 35%, 50% and 95%. The selection of 95% of peptides gave the same distributions of residues as it is presented in Fig. 2a.

**Supplementary Table 1. Summary of the data sets of peptides for cathepsins K, V, B, L, S, and F.**

**a. Unique and shared cleavage sites in substrates that were cleaved by cathepsins (Cat) K, V, B, L, S, and F are presented.** The columns present the number of peptides (A), number of originated proteins of peptides (B), number of unique cleavage sites (C) and its percentage share (C%), number of shared cleavages of 2 cathepsins (D) and its percentage share (D%), number of shared cleavages of 3 cathepsins (E) and its percentage share (E%), number of shared cleavages of 4 cathepsins (F) and its percentage share (F%), number of shared cleavages of 5 cathepsins (G) and its percentage share (G%), and number of shared cleavages of 6 cathepsins (H) and its percentage share (H%). The total number of identified cleavage sites was 29,674. Each cathepsin contributed between 3,500 and 9,583 cleavage sites in 3,167 proteins. A total of 1,592 of these proteins had at least a partial 3D structure in the protein structure database PDB (in January 2019, with 158,934 entries and 11,505 structures released annually[24]). In the case of multiple entries of the same protein, the entry with the highest sequence coverage and resolution was used in our analysis. The highest number of detected cleavages in one protein was 178 (cellular myosin with 1,960 residues and UniProt code P35579). Interestingly, among the shared cleavages, 243 were performed by all six cathepsins (Columns H and H (%)).

| Cath | A | B | C | C% | D | D% | E | E% | F | F% | G | G% | H | H% |
|---|---|---|---|---|---|---|---|---|---|---|---|---|---|---|
| K | 9583 | 2330 | 5182 | 54 | 1748 | 18 | 1174 | 12 | 695 | 7 | 541 | 6 | 243 | 3 |
| V | 4415 | 1501 | 756 | 17 | 1456 | 33 | 827 | 19 | 597 | 14 | 536 | 12 | 243 | 5 |
| B | 4254 | 1335 | 2192 | 52 | 829 | 19 | 480 | 11 | 313 | 7 | 197 | 5 | 243 | 6 |
| L | 4117 | 1454 | 780 | 19 | 1322 | 32 | 746 | 18 | 531 | 13 | 495 | 12 | 243 | 6 |
| S | 3805 | 1488 | 904 | 24 | 793 | 21 | 747 | 20 | 591 | 15 | 527 | 14 | 243 | 6 |
| F | 3500 | 1264 | 772 | 22 | 792 | 23 | 676 | 19 | 513 | 15 | 504 | 14 | 243 | 7 |
| Total | 29674 | | 10586 | 36 | 6940 | 23 | 4650 | 16 | 3240 | 11 | 2800 | 9 | 1458 | 5 |

**The frequencies (No) and shares (%) of combinations of shared cleavages** of **b.** two, **c.** three, **d.** four, and **e.** five cathepsins are also presented. Interestingly, all possible combinations of shared cleavages of cathepsins appeared, but some were very rare.

**b**. Combinations of shared cleavages of two cathepsins.

| Cat | Cat | No | % |
|---|---|---|---|
| V | L | 961 | 28 |
| K | B | 459 | 13 |
| K | F | 395 | 11 |
| K | S | 363 | 10 |
| K | V | 318 | 9 |
| K | L | 213 | 6 |
| S | F | 156 | 5 |
| B | F | 145 | 4 |
| B | S | 135 | 4 |
| V | F | 74 | 2 |
| V | S | 73 | 2 |
| L | S | 66 | 2 |
| B | L | 60 | 2 |
| V | B | 30 | 1 |
| L | F | 22 | 1 |

**c.** Combinations of shared cleavages of three cathepsins.

| Cat | Cat | Cat | No | % |
|---|---|---|---|---|
| K | V | L | 359 | 23 |
| K | S | F | 244 | 16 |
| K | B | F | 153 | 10 |
| V | L | S | 122 | 8 |
| K | B | S | 120 | 8 |
| K | V | S | 75 | 5 |
| K | V | F | 73 | 5 |
| V | L | F | 57 | 4 |
| V | B | L | 54 | 3 |
| K | L | S | 53 | 3 |
| B | S | F | 52 | 3 |
| K | B | L | 34 | 2 |
| V | S | F | 32 | 2 |
| K | L | F | 32 | 2 |
| K | V | B | 31 | 2 |
| L | S | F | 23 | 1 |
| V | B | S | 16 | 1 |
| B | L | S | 10 | 1 |
| V | B | F | 8 | 1 |
| B | L | F | 2 | 0 |

**d.** Combinations of shared cleavages of four cathepsins.

| Cat | Cat | Cat | Cat | No | % |
|---|---|---|---|---|---|
| K | V | L | S | 166 | 21 |
| K | B | S | F | 126 | 16 |
| K | V | L | F | 119 | 15 |
| K | V | S | F | 97 | 12 |
| K | V | B | L | 62 | 8 |
| V | L | S | F | 59 | 7 |
| K | L | S | F | 56 | 7 |
| V | B | L | S | 30 | 4 |
| K | V | B | S | 27 | 3 |
| K | V | B | F | 20 | 2 |
| K | B | L | S | 12 | 1 |
| K | B | L | F | 10 | 1 |
| V | B | S | F | 9 | 1 |
| B | L | S | F | 9 | 1 |
| V | B | L | F | 8 | 1 |

**e.** Combinations of shared cleavages of five cathepsins.

| Cat | Cat | Cat | Cat | Cat | No | % |
|---|---|---|---|---|---|---|
| K | V | L | S | F | 363 | 65 |
| K | V | B | S | F | 65 | 12 |
| K | V | B | L | S | 56 | 10 |
| K | V | B | L | F | 33 | 6 |
| K | B | L | S | F | 24 | 4 |
| V | B | L | S | F | 19 | 3 |

**Supplementary Table 2. Parameters of support vector machine SVM models and predictions of cathepsins' cleavage sites.**

**a. SVM models**. The columns list the cathepsins (Cat), number of positive peptides (NoP), number of negative peptides (NoN), true positive rate critical point (TPR), false positive rate critical point (FPR), accuracy (Accuracy), and total number of testing peptides (NoT).

| Cat | NoP | NoN | TPR | FPR | Accuracy (%) | NoT |
|---|---|---|---|---|---|---|
| K | 2,253 | 3,526 | 0.802 | 0.198 | 80 | 10,466 |
| V | 2,081 | 4,508 | 0.833 | 0.167 | 85 | 2,002 |
| B | 4,006 | 4,508 | 0.809 | 0.192 | 89 | 1,326 |
| L | 1,938 | 3,948 | 0.848 | 0.152 | 88 | 1,978 |
| S | 1,792 | 3,526 | 0.84 | 0.160 | 87 | 1,773 |
| F | 3,277 | 4,508 | 0.828 | 0.173 | 91 | 993 |

**b**. **Predictions of cathepsins' cleavage sites.** The first column lists the virus and its protein with the UniProt code. The second column lists the predicted cleavage sites. Columns 3 to 8 contain the calculated false positive rates (FPRs), which are shown in bold when cleavages are predicted. In this case, the FPR values were less than or equal to 0.198 (the highest cathepsin critical value of FPR in Supplementary Table 2a). Identified cleavage sites of furin or cathepsin L or other proteases that were experimentally confirmed in the literature are marked with asterisks (*). The cleavage site that was confirmed in vitro in the JSI lab is marked with two asterisks (**). References are listed for the spike (S) protein of viruses SARS-CoV-2[9], SARS-CoV[8] and MERS-CoV[71].

| | | FPR of cathepsin cleavage sites | | | | | |
|---|---|---|---|---|---|---|---|
| Virus, its protein with UniProt code/cleaved by protease | Cleavage site | L | V | K | S | B | F |
| SARS-CoV-2, S protein, P0DTC2 | | | | | | | |
| | Q675-T676 | **0.118** | **0.092** | 0.608 | 0.219 | 0.437 | 0.267 |
| | Q677-T678 | 0.426 | 0.296 | 0.392 | **0.160** | 0.274 | **0.176** |
| | N679-S680 | 0.966** | 0.984 | 0.949 | 0.940 | 0.945 | 0.951 |
| Furin[9] | R685-S686* | **0.194** | 0.265 | **0.107** | **0.091** | **0.167** | **0.109** |
| | T696-M697* | **0.162** | **0.132** | 0.334 | **0.172** | 0.353 | 0.319 |
| | M697-S698 | 0.406 | 0.425 | 0.677 | 0.576 | **0.108**** | 0.240 |
| | S698-L699 | 0.327 | 0.311 | **0.164** | **0.068** | **0.050** | **0.063** |
| | **G700-A701**** | **0.018**** | **0.018** | **0.032** | **0.021**** | 0.348 | **0.001** |
| | E702-N703 | 0.516 | 0.265 | **0.121** | 0.220 | 0.352 | 0.208 |
| | S816-F817 | 0.408 | 0.395 | 0.524 | 0.574 | **0.096** | 0.633 |
| | F817-I818 | 0.684 | 0.868 | 0.441 | 0.345 | **0.177** | 0.445 |
| SARS-CoV, S protein, P59594 | | | | | | | |
| Trypsin[8] | R667-S668* | **0.080** | **0.095** | **0.139** | **0.079** | 0.250 | **0.060** |
| Cathepsin L[8] | T678-M679* | **0.111** | **0.149** | 0.325 | **0.177** | 0.235 | 0.426 |
| MERS-CoV, S protein, W6A028 | | | | | | | |
| Furin[71] | R751-S752* | **0.062** | **0.043** | **0.119** | **0.013** | **0.040** | **0.019** |
| Furin[71] | R887-S888* | 0.337 | 0.397 | **0.163** | **0.069** | 0.213 | 0.710 |

**Supplementary Table 3. Selected peptide sequences for complexes with mutated cathepsin V.**

The length of peptides is 6–11 residues, which are inserted in the columns at positions P5–P6'. The cleavage site is between P1–P1'. The cleavage site cluster for cathepsin V is presented under the cluster column (for example, V1 for cathepsin V cluster 1), as well as for cathepsins K, L, B, F, and S, when they had the same cleavage site. Residues that are shaded represent dominant residues in the corresponding cathepsin V cluster. If the sequence was found to have multiple cleavage sites, it is referred to as cleavage area, and if the sequence had only one cleavage site, it is referred to as positional cleavage, in the cleavage type column. Indices 1 and 2 refer to one or two separated cleavage areas in the originated protein, respectively, where cathepsin(s) acted. Indices 3 and 4 mark where there were only one or two cleavage sites in the originated protein, respectively. Termini of most peptides were protected with N-acetylation and C-amidation (marked “Y” in protection column). Some peptides were also synthesized without protection (marked “N”) or with and without protection (marked “Y/N”). Peptides have UniProt codes (www.uniprot.org) of their corresponding proteins.

| No | P5 | P4 | P3 | P2 | P1 | P1’ | P2’ | P3’ | P4’ | P5’ | P6’ | Cluster | Cleavage type | Prote-ction | UniProt |
|---|---|---|---|---|---|---|---|---|---|---|---|---|---|---|---|
| p1 | | T | C | L | C | Q | V | P | Q | | | **V1**, K3, F1, S1 | Positional | Y | P49588 |
| p2 | | I | L | L | T | E | A | P | L | | | **V1**, K3, L2, B1, F1, S1 | Area | Y | Q6S8J3, P0CG38, P0CG39, Q9BYX7 |
| p3 | | K | D | L | L | H | P | S | P | | | **V1** | Area[1] | Y | P42677 |
| p4 | E | I | D | L | R | N | P | K | G | N | | **V1**, L1 | Area | Y | P27695 |
| p5 | | Q | L | L | V | A | C | K | V | K | | **V1**, L2 | Positional | Y | Q9Y490 |
| p6 | | K | V | L | A | T | V | T | K | | | **V1**, F1, K3, S1 | Area | Y | Q02878 |
| p7 | | | R | L | S | A | K | P | | | | **V1**, K3, B1, L1, S1, F1, K3, L1 | Area, Positional | Y/N | Q15651, O00479* |
| p8 | | | L | L | S | G | K | E | | | | **V1**, K3, L2 | Area | Y/N | A6NHL2 |
| p9 | | | Q | L | R | Q | Q | E | | | | **V1**, K5, L1, S7 | Positional[4] | Y/N | O43818 |
| p10 | | G | N | Y | K | E | A | K | K | | | **V2** | Positional | Y | P42704 |
| p11 | | V | L | L | K | V | A | A | S | | | **V2**, L3 | Positional[3] | Y | Q53FA7 |
| p12 | | A | C | M | K | S | V | T | E | | | **V2**, F1, K4, S4 | Area | Y | P63104 |
| p13 | | V | A | C | K | S | S | Q | P | | | **V2**, B1, F1, K6, L3, S8 | Positional | Y | P46013 |
| p14 | | | G | A | K | S | A | A | | | | **V2**, K6, B1, L3, F1 | Area | Y/N | Q8NC51 |
| p15 | | | G | V | T | K | A | A | | | | **V3**, B1, F2, K1, L4, S2 | Area | Y | P27797** |
| p16 | | | G | M | C | K | A | G | | | | **V3**, F2, K1, S6 | Area | Y | Q6S8J3, P60709, P63261, A5A3E0, P0CG38, P0CG39 |
| p17 | | | K | I | A | K | T | H | | | | **V3** | Positional | Y | O75533 |

| | | | | | | | | | | | | | | | |
|---|---|---|---|---|---|---|---|---|---|---|---|---|---|---|---|
| p18 | | | E | V | C | K | K | K | K | | | **V3**, F2, K1, L4, S6 | Positional[3] | Y | Q92772 |
| p19 | | | I | I | L | K | E | K | | | | **V3**, K1, L4 | Positional[3] | Y/N | P07199 |
| p20 | | R | G | I | R | E | A | A | K | | | **V4**, K5, B1, L1, S2, F1 | Positional | Y | P25398 |
| p21 | | K | R | F | Q | N | V | A | K | | | **V4**, F1, K5, S5 | Area | Y | P14625 |
| p22 | | | A | Y | F | K | K | V | L | | | **V5** | Area | Y | P25205 |
| p23 | | | V | Y | E | K | K | P | | | | **V5**, L4, S5, F2 | Area | Y/N | P46777 |
| p24 | | S | I | Y | E | V | D | K | Q | | | **V6** | Positional | Y | Q92747 |
| p25 | | T | R | E | S | E | D | L | E | | | **V6**, B1, F1, K2, L1, S3 | Positional[3] | Y | Q8N5V2 |
| p26 | | V | P | C | G | T | A | H | E | | | **V6** | Positional | Y | O43823 |
| p27 | | K | K | Y | D | A | F | L | A | | | **V6**, L1 | Positional | Y | P62906 |
| p28 | | | A | W | K | K | E | A | | | | **V7**, L4 | Positional[4] | Y | Q9C0B0 |
| p29 | | | P | V | K | K | K | A | K | | | **V7**, F2, K4, S4 | Area | Y | P16402 |
| p30 | | | K | P | K | K | K | T | K | | | **V7**, L4 | Area | Y | Q6NWY9 |
| p31 | | | L | L | K | V | A | L | | | | **V2**, L3, B1, F1, K4, S4 | | N | 11,759*** |
| p32 | | | A | V | A | E | K | Q | | | | **V4**, L1, B1, F1, K1, S2 | | N | 6,261*** |
| p33 | | | A | L | A | A | S | S | | | | **V1**, L1, B1, F1, K3, S1 | | N | 40,482*** |
| p34 | | | A | V | R | A | R | L | | | | **V4**, L1, B1, F1, K3, S2 | | N | 33,891*** |
| p35 | | | L | L | K | A | V | A | E | K | Q | **V2**, L3, B1, F1, K4, S4 | | Y | 1*** |

* Proteins O00479 and QI5652 were cleaved in the same location but with different cathepsins.

** Amount of peptide was sufficient to carry out structural analysis only.

*** Peptide was not part of our data sets (Supplementary Data 1). The number presents sequences found in the UniProt database.

**Supplementary Table 4. Summary of peptide binding to crystals of cathepsin V C25S/A.**

Peptide number and their corresponding sequences and clusters are shown in columns No, Sequence and Cluster, respectively. Peptide residues modeled to free–kick omit map (Fo-Fc) are marked in the columns that denote peptide positions P8–P6'. MPD is 2-methyl-2,4-pentanediol. Chlorine anion is marked as $CL^-$. Molecule column with “A” or “B” denotes 2 cathepsin V molecules in the asymmetric unit. Mutant column shows mutation at catalytic Cys residue: C25A or C25S. “Y” and “N” in the Protection column stand for peptide termini protection “yes” and “no”. “s”, “c”, or “b” in the Method column stand for soaking, co-crystallization, or both techniques, respectively. PDB column contains the PDB codes of structures. Four patterns of peptide binding to cathepsin V were observed; table is divided into four parts: I, binding of cleaved peptide fragments to the non-primed site; II, binding shifted to the non-primed site; III, binding shifted to the primed site; and IV, binding of part cleaved peptides across the active site. Peptides exhibiting multiple binding patterns with respect to position in the asymmetric unit or crystallization method used are highlighted with colors in the sequence column. Peptides with the same sequence but different termini are not considered as equivalent peptides.

| No. | Sequence | Cluster | P8 | P7 | P6 | P5 | P4 | P3 | P2 | P1 | P1' | P2' | P3' | P4' | P5' | P6' | Molecule/ Mutant | Protection | Method | PDB code |
|---|---|---|---|---|---|---|---|---|---|---|---|---|---|---|---|---|---|---|---|---|
| **Pattern I. Binding of cleaved peptide fragments at the non-primed site** | | | | | | | | | | | | | | | | | | | | |
| p13 | VACKSSQP | 2 | | | | | **V** | **A** | **C** | **K** | MPD | | | | | | A, B / C25A | Y | s | 7QFF |
| p23 | VYEKKP | 5 | | | | | | **V** | **Y** | **E** | MPD | | | | | | A, B / C25S | N | s | 7QNS |
| p14 | GAKSAA | 2 | | | | | | **G** | **A** | **K** | MPD | | | | | | A / C25A | N | s | 7QO2 |
| p8 | LLSGKE | 1 | | | | | | **L** | **L** | **S** | MPD | | | | | | B / C25A | N | s | 7Q8O |
| p31 | LLKVAL | 2 | | | | | | **L** | **L** | **K** | MPD | | | | | | A, B / C25S | N | c | 7Q8K |
| p35 | LLKAVAEKQ | 2 | | | | | | **L** | **L** | **K** | MPD | | | | | | B / C25A | Y | b | 7Q9H |
| **Pattern II. Binding shifted to the non-primed site** | | | | | | | | | | | | | | | | | | | | |
| p18 | EVCKKKK | 3 | | **E** | **V** | **C** | **K** | **K** | **K** | **K** | MPD | | | | | | A / C25A | Y | s | 7Q8H |
| p22 | AYFKKVL | 5 | | A | Y | F | K | **K** | **V** | **L** | MPD | | | | | | B / C25A | Y | s | 7QFH |
| p7 | RLSAKP | 1 | | | R | **L** | **S** | **A** | **K** | **P** | MPD | | | | | | B / C25A | Y | b | 7Q9C |
| p25 | TRESEDLE | 6 | T | R | E | S | E | **D** | **L** | **E** | MPD | | | | | | A, B / C25A | Y | s | 7Q8D |
| p10 | GNYKEAKK | 2 | **G** | **N** | **Y** | **K** | **E** | **A** | **K** | **K** | MPD | | | | | | A / C25A | Y | s | 7Q8F |
| p30 | KPKKKTK | 7 | | K | P | K | **K** | **K** | **T** | **K** | MPD | | | | | | B / C25A | Y | s | 7Q8M |
| p14 | GAKSAA | 2 | | | G | A | K | **S** | **A** | **A** | MPD | | | | | | A, B / | Y | b | 7QHJ |

| | | | | | | | | | | | | | | | | | | | | |
|---|---|---|---|---|---|---|---|---|---|---|---|---|---|---|---|---|---|---|---|---|
| | | | | | | | | | | | | | | | | | C25A | | | |
| p27 | KKYDAFLA | 6 | K | K | Y | D | A | **F** | **L** | **A** | MPD | | | | | | A, B / C25A | Y | s | 7Q8N |
| p26 | VPCGTAHE | 6 | V | P | C | G | **T** | **A** | **H** | **E** | MPD | | | | | | A, B / C25A | Y | s | 7Q8L |
| p9 | QLRQQE | 1 | | | Q | L | R | **Q** | **Q** | **E** | MPD | | | | | | B / C25A | N | s | 7QHK |
| **Pattern III. Binding shifted to the primed site** | | | | | | | | | | | | | | | | | | | | |
| p8 | LLSGKE | 1 | | | | | | MPD | | $CL^-$ | **L** | **L** | **S** | G | K | E | A / C25A | N | s | 7Q8O |
| p9 | QLRQQE | 1 | | | | | | MPD | | $CL^-$ | **Q** | **L** | **R** | **Q** | Q | E | A / C25A | N | s | 7QHK |
| p7 | RLSAKP | 1 | | | | | | MPD | | $CL^-$ | **R** | **L** | **S** | **A** | **K** | **P** | B / C25S | N | s | 7Q8Q |
| p14 | GAKSAA | 2 | | | | | | MPD | | $CL^-$ | **G** | **A** | **K** | **S** | A | A | A / C25A | N | s | 7QO2 |
| p19 | IILKEK | 3 | | | | | | MPD | | $CL^-$ | **I** | **I** | **L** | K | E | K | A / C25S | N | s | 7Q8J |
| p31 | LLKVAL | 2 | | | | | | MPD | | $CL^-$ | **L** | **L** | **K** | **V** | A | L | A / C25S | N | s | 7Q8P |
| p32 | AVAEKQ | 4 | | | | | | MPD | | $CL^-$ | **A** | **V** | **A** | **E** | **K** | **Q** | B / C25S | N | s | 7Q8I |
| p33 | ALAASS | 1 | | | | | | MPD | | $CL^-$ | **A** | **L** | **A** | **A** | S | S | A / C25S | N | s | 7Q8G |
| **Pattern IV. Binding of cleaved peptide fragments across the active site** | | | | | | | | | | | | | | | | | | | | |
| p7 | RLSAKP | 1 | | | | | | **R** | **L** | **S** | **A** | **K** | **P** | | | | A / C25A | Y | b | 7Q9C |
| p35 | LLKAVAEKQ | 2 | | | | | | **L** | **L** | **K** | **A** | **V** | **A** | **E** | **K** | Q | A / C25A | Y | b | 7Q9H |

## Supplementary Table 5. Data collection and refinement statistics: PDB entries 7Q8H and 7Q8D.

| | **7Q8H** | **7Q8D** |
|---|---|---|
| **Data collection** | | |
| Space group | P 43 21 2 | P 43 21 2 |
| Cell dimensions□□ | | |
| *a*, *b*, *c* (Å) | 93.75, 93.75, 124.29 | 93.82, 93.82, 124.92 |
| □□□□□□□□□□□□□ (□) | 90.00 90.00 90.00 | 90.00 90.00 90.00 |
| Resolution (Å) | 46.87 – 1.75 (1.86 – 1.75) | 41.96 – 1.80 (1.91 – 1.80) |
| $R_{sym}$ or $R_{merge}$ | 0.12 (1.19) | 0.11 (1.51) |
| *I* / □*I* | 10.17 (1.40) | 13.42 (1.28) |
| Completeness (%) | 99.8 (99.2) | 99.6 (97.6) |
| Redundancy | 10.1 (10.2) | 13.7 (13.6) |
| | | |
| **Refinement** | | |
| Resolution (Å) | 46.87 – 1.75 | 41.96 – 1.80 |
| No. reflections | 56397 | 52223 |
| $R_{work}$ / $R_{free}$ | 0.16 / 0.18 | 0.17 / 0.20 |
| No. atoms | | |
| Protein | 3373 | 3373 |
| Ligand | 102 | 52 |
| Water | 420 | 392 |
| *B*-factors | | |
| Protein | 23.35 | 30.50 |
| Ligand | 64.02 | 55.96 |
| Water | 48.13 | 52.05 |
| R.m.s. deviations | | |
| Bond lengths (Å) | 0.018 | 0.015 |
| Bond angles (□) | 1.8 | 1.65 |

*Data was collected from one crystal. *Hydrogen atoms were excluded from calculations.

**Supplementary Table 6. Data collection and refinement statistics:** PDB entries **7Q8F and 7Q8L.**

| | **7Q8F** | **7Q8L** |
|---|---|---|
| **Data collection** | | |
| Space group | P 43 21 2 | P 43 21 2 |
| Cell dimensions□□ | | |
| *a*, *b*, *c* (Å) | 94.32, 94.32, 125.58 | 94.35, 94.35, 127.26 |
| □□□□□□□□□□□□ (□) | 90.00 90.00 90.00 | 90.00 90.00 90.00 |
| Resolution (Å) | 47.16 – 1.49 (1.58 – 1.49) | 47.18 – 1.80 (1.91 – 1.80) |
| $R_{sym}$ or $R_{merge}$ | 0.13 (2.19) | 0.12 (1.45) |
| *I* / □*I* | 16.64 (0.94) | 11.36 (1.12) |
| Completeness (%) | 99.9 (99.6) | 99.9 (99.6) |
| Redundancy | 24 (21.3) | 12.8 (12.1) |
| | | |
| **Refinement** | | |
| Resolution (Å) | 47.16 – 1.49 | 47.18 – 1.80 |
| No. reflections | 92736 | 53524 |
| $R_{work}$ / $R_{free}$ | 0.18 / 0.20 | 0.17 / 0.19 |
| No. atoms | | |
| Protein | 3370 | 3372 |
| Ligand | 116 | 64 |
| Water | 428 | 492 |
| *B*-factors | | |
| Protein | 24.14 | 41.75 |
| Ligand | 60.65 | 97.88 |
| Water | 45.53 | 66.01 |
| R.m.s. deviations | | |
| Bond lengths (Å) | 0.018 | 0.014 |
| Bond angles (□) | 1.85 | 1.7 |

*Data was collected from one crystal. *Hydrogen atoms were excluded from calculations.

**Supplementary Table 7. Data collection and refinement statistics:** PDB entries **7Q8M and 7Q8N.**

| **Data collection** | **7Q8M** | **7Q8N** |
|---|---|---|
| Space group | P 43 21 2 | P 43 21 2 |
| Cell dimensions□□ | | |
| *a*, *b*, *c* (Å) | 94.23, 94.23, 125.61 | 94.15, 94.15, 126.10 |
| □□□□□□□□□□□□ (□) | 90.00 90.00 90.00 | 90.00 90.00 90.00 |
| Resolution (Å) | 47.11 – 1.57 (1.67 – 1.57) | 45.78 – 2.00 (2.12 – 2.00) |
| $R_{sym}$ or $R_{merge}$ | 0.06 / 1.15 | 0.12 (1.06) |
| *I* / □*I* | 21.87 (1.99) | 11.48 (1.86) |
| Completeness (%) | 99.9 (99.5) | 99.9 (99.9) |
| Redundancy | 24.8 (21.9) | 14.3 (13.5) |
| | | |
| **Refinement** | | |
| Resolution (Å) | 47.11 – 1.57 | 45.78 – 2.00 |
| No. reflections | 79145 | 38867 |
| $R_{work}$ / $R_{free}$ | 0.19 / 0.21 | 0.17 / 0.20 |
| No. atoms | | |
| Protein | 3367 | 3383 |
| Ligand | 60 | 50 |
| Water | 395 | 322 |
| *B*-factors | | |
| Protein | 29.49 | 32.95 |
| Ligand | 57.78 | 86.79 |
| Water | 51.30 | 48.22 |
| R.m.s. deviations | | |
| Bond lengths (Å) | 0.015 | 0.017 |
| Bond angles (□) | 1.7 | 1.7 |

*Data was collected from one crystal. *Hydrogen atoms were excluded from calculations.

**Supplementary Table 8. Data collection and refinement statistics:** PDB entries **7Q8I and 7Q9C.**

| | | |
|---|---|---|
| **Data collection** | **7Q8I** | **7Q9C** |
| Space group | P 43 21 2 | P 43 21 2 |
| Cell dimensions□□ | | |
| *a*, *b*, *c* (Å) | 93.48, 93.48, 125.87 | 94.10, 94.10, 124.75 |
| □□□□□□□□□□□□ (□) | 90.00 90.00 90.00 | 90.00 90.00 90.00 |
| Resolution (Å) | 46.74 – 1.59 (1.69 – 1.59) | 47.05 – 1.32 (1.40 – 1.32) |
| $R_{sym}$ or $R_{merge}$ | 0.11 (0.89) | 0.07 (1.89) |
| *I* / □*I* | 16.99 (2.72) | 16.05 (0.65) |
| Completeness (%) | 99.9 (99.2) | 99.6 (97.6) |
| Redundancy | 13.5 (13.6) | 11.9 (5.3) |
| | | |
| **Refinement** | | |
| Resolution (Å) | 46.74 – 1.59 | 47.05 – 1.4 |
| No. reflections | 75384 | 110243 |
| $R_{work}$ / $R_{free}$ | 0.17 / 0.19 | 0.18 / 0.21 |
| No. atoms | | |
| Protein | 3379 | 3372 |
| Ligand | 80 | 84 |
| Water | 402 | 489 |
| *B*-factors | | |
| Protein | 20.87 | 20.32 |
| Ligand | 31.67 | 69.90 |
| Water | 37.12 | 55.31 |
| R.m.s. deviations | | |
| Bond lengths (Å) | 0.017 | 0.014 |
| Bond angles (□) | 1.8 | 1.7 |

*Data was collected from one crystal. *Hydrogen atoms were excluded from calculations.

**Supplementary Table 9. Data collection and refinement statistics:** PDB entries **7Q9H and 7QHJ.**

| **Data collection** | **7Q9H** | **7QHJ** |
|---|---|---|
| Space group | P 43 21 2 | P 43 21 2 |
| Cell dimensions□□ | | |
| *a*, *b*, *c* (Å) | 94.51, 94.51, 124.78 | 94.31, 94.31, 125.37 |
| □□□□□□□□□□□□ (□) | 90.00 90.00 90.00 | 90.00 90.00 90.00 |
| Resolution (Å) | 47.26 – 1.29 (1.37 – 1.29) | 47.16 – 1.35 (1.43 – 1.35) |
| $R_{sym}$ or $R_{merge}$ | 0.09 (2.9) | 0.08 (4.1) |
| *I* / □*I* | 16.44 (0.76) | 20.60 (0.57) |
| Completeness (%) | 99.4 (97.4) | 99.6 (97.6) |
| Redundancy | 13.5 (13.4) | 13.5 (12.7) |
| | | |
| **Refinement** | | |
| Resolution (Å) | 47.26 – 1.4 | 45.67 – 1.40 |
| No. reflections | 141576 | 111254 |
| $R_{work}$ / $R_{free}$ | 0.18 / 0.20 | 0.19 / 0.21 |
| No. atoms | | |
| Protein | 3374 | 3373 |
| Ligand | 93 | 34 |
| Water | 507 | 502 |
| *B*-factors | | |
| Protein | 17.40 | 21.97 |
| Ligand | 41.30 | 36.01 |
| Water | 42.22 | 48.17 |
| R.m.s. deviations | | |
| Bond lengths (Å) | 0.022 | 0.016 |
| Bond angles (□) | 2.1 | 1.7 |

*Data was collected from one crystal. *Hydrogen atoms were excluded from calculations.

**Supplementary Table 10. Data collection and refinement statistics:** PDB entries **7Q8K and 7Q8P.**

| **Data collection** | **7Q8K** | **7Q8P** |
|---|---|---|
| Space group | P 43 21 2 | P 43 21 2 |
| Cell dimensions□□ | | |
| *a*, *b*, *c* (Å) | 96.11, 96.11, 125.57 | 94.20, 94.20, 126.18 |
| □□□□□□□□□□□□ (□) | 90.00 90.00 90.00 | 90.00 90.00 90.00 |
| Resolution (Å) | 48.06 – 1.74 (1.85 – 1.74) | 47.10 – 1.71 (1.81 – 1.71) |
| $R_{sym}$ or $R_{merge}$ | 0.12 (0.93) | 0.11 (1.01) |
| *I* / □*I* | 14.89 (2.47) | 15.19 (2.43) |
| Completeness (%) | 99.5 (97.0) | 99.9 (99.5) |
| Redundancy | 13.3 (12.2) | 13.4 (13.2) |
| | | |
| **Refinement** | | |
| Resolution (Å) | 48.06 – 1.74 | 47.10 – 1.71 |
| No. reflections | 60130 | 62023 |
| $R_{work}$ / $R_{free}$ | 0.17 / 0.20 | 0.18 / 0.20 |
| No. atoms | | |
| Protein | 3369 | 3381 |
| Ligand | 51 | 60 |
| Water | 379 | 353 |
| *B*-factors | | |
| Protein | 25.81 | 28.11 |
| Ligand | 31.78 | 57.97 |
| Water | 39.80 | 43.02 |
| R.m.s. deviations | | |
| Bond lengths (Å) | 0.018 | 0.012 |
| Bond angles (□) | 2.1 | 1.6 |

*Data was collected from one crystal. *Hydrogen atoms were excluded from calculations.

**Supplementary Table 11. Data collection and refinement statistics:** PDB entries **7QFF and 7QFH.**

| **Data collection** | **7QFF** | **7QFH** |
|---|---|---|
| Space group | P 43 21 2 | P 43 21 2 |
| Cell dimensions□□ | | |
| *a*, *b*, *c* (Å) | 94.40, 94.40, 126.83 | 94.09, 94.09, 126.42 |
| □□□□□□□□□□□□ (□) | 90.00 90.00 90.00 | 90.00 90.00 90.00 |
| Resolution (Å) | 47.20 – 1.50 (1.59 – 1.50) | 45.83 – 1.52 (1.61 – 1.52) |
| $R_{sym}$ or $R_{merge}$ | 0.05 (1.11) | 0.09 (2.17) |
| *I* / □*I* | 23.73 (1.69) | 13.56 (0.76) |
| Completeness (%) | 99.9 (99.6) | 99.9 (99.8) |
| Redundancy | 13.8 (13.3) | 7.3 (7.4) |
| | | |
| **Refinement** | | |
| Resolution (Å) | 47.20 – 1.50 | 45.83 – 1.52 |
| No. reflections | 91915 | 87563 |
| $R_{work}$ / $R_{free}$ | 0.17 / 0.19 | 0.18 / 0.21 |
| No. atoms | | |
| Protein | 3378 | 3361 |
| Ligand | 62 | 46 |
| Water | 530 | 437 |
| *B*-factors | | |
| Protein | 24.99 | 21.88 |
| Ligand | 60.86 | 56.12 |
| Water | 49.41 | 47.34 |
| R.m.s. deviations | | |
| Bond lengths (Å) | 0.016 | 0.020 |
| Bond angles (□) | 1.7 | 1.8 |

*Data was collected from one crystal. *Hydrogen atoms were excluded from calculations.

**Supplementary Table 12. Data collection and refinement statistics:** PDB entries **7Q8G and 7Q8O.**

| | | |
|---|---|---|
| **Data collection** | **7Q8G** | **7Q8O** |
| Space group | P 43 21 2 | P 43 21 2 |
| Cell dimensions□□ | | |
| *a*, *b*, *c* (Å) | 94.03, 94.03, 125.68 | 93.54, 93.54, 124.09 |
| □□□□□□□□□□□□ (□) | 90.00 90.00 90.00 | 90.00 90.00 90.00 |
| Resolution (Å) | 45.67 – 2.06 (2.18 – 2.06) | 46.77 – 1.90 (2.02 – 1.90) |
| $R_{sym}$ or $R_{merge}$ | 0.10 (0.71) | 0.20 (1.15) |
| *I* / □*I* | 12.23 (2.11) | 9.83 (1.82) |
| Completeness (%) | 99.6 (99.7) | 100 (99.9) |
| Redundancy | 13.3 (12.8) | 7.8 (7.8) |
| | | |
| **Refinement** | | |
| Resolution (Å) | 45.67 – 2.06 | 46.77 – 1.90 |
| No. reflections | 35511 | 43694 |
| $R_{work}$ / $R_{free}$ | 0.18 / 0.21 | 0.18 / 0.21 |
| No. atoms | | |
| Protein | 3382 | 3371 |
| Ligand | 49 | 45 |
| Water | 335 | 330 |
| *B*-factors | | |
| Protein | 35.95 | 21.46 |
| Ligand | 72.32 | 44.10 |
| Water | 52.05 | 35.07 |
| R.m.s. deviations | | |
| Bond lengths (Å) | 0.017 | 0.017 |
| Bond angles (□) | 1.7 | 2.0 |

*Data was collected from one crystal. *Hydrogen atoms were excluded from calculations.

**Supplementary Table 13. Data collection and refinement statistics:** PDB entries **7Q8J and 7QHK.**

| **Data collection** | **7Q8J** | **7QHK** |
|---|---|---|
| Space group | P 43 21 2 | P 43 21 2 |
| Cell dimensions□□ | | |
| *a*, *b*, *c* (Å) | 93.82, 93.82, 125.63 | 93.69, 93.69, 124.75 |
| □□□□□□□□□□□□ (□) | 90.00 90.00 90.00 | 90.00 90.00 90.00 |
| Resolution (Å) | 46.91 – 1.64 (1.74 – 1.64) | 45.42 – 1.83 (1.94 – 1.83) |
| $R_{sym}$ or $R_{merge}$ | 0.08 (1.18) | 0.08 (0.69) |
| *I* / □*I* | 21.01 (1.94) | 18.13 (1.96) |
| Completeness (%) | 99.8 (99.1) | 96.7 (83.1) |
| Redundancy | 12.9 (11.3) | 7 (3.9) |
| | | |
| **Refinement** | | |
| Resolution (Å) | 46.91 – 1.64 | 45.42 – 1.83 |
| No. reflections | 68906 | 48185 |
| $R_{work}$ / $R_{free}$ | 0.19 / 0.22 | 0.17 / 0.20 |
| No. atoms | | |
| Protein | 3375 | 3377 |
| Ligand | 52 | 65 |
| Water | 311 | 393 |
| *B*-factors | | |
| Protein | 27.90 | 25.47 |
| Ligand | 42.60 | 84.54 |
| Water | 43.99 | 49.22 |
| R.m.s. deviations | | |
| Bond lengths (Å) | 0.017 | 0.018 |
| Bond angles (□) | 1.8 | 1.8 |

*Data was collected from one crystal. *Hydrogen atoms were excluded from calculations.

**Supplementary Table 14. Data collection and refinement statistics:** PDB entries **7Q8Q and 7QNS.**

| | | |
|---|---|---|
| **Data collection** | **7Q8Q** | **7QNS** |
| Space group | P 43 21 2 | P 43 21 2 |
| Cell dimensions | | |
| *a*, *b*, *c* (Å) | 92.75, 92.75, 128.09 | 93.97, 93.97, 124.06 |
| [illegible] ([illegible]) | 90.00 90.00 90.00 | 90.00 90.00 90.00 |
| Resolution (Å) | 43.61 – 2.13 (2.26 – 2.13) | 43.94 – 1.40 (1.48 – 1.40) |
| $R_{sym}$ or $R_{merge}$ | 0.13 (1.09) | 0.06 (1.05) |
| *I* / [illegible]*I* | 14.20 (1.84) | 25.16 (1.97) |
| Completeness (%) | 99.3 (96.4) | 99.6 (97.5) |
| Redundancy | 8.6 (8.4) | 12.5 (8.4) |
| | | |
| **Refinement** | | |
| Resolution (Å) | 43.61 – 2.13 | 43.94 – 1.40 |
| No. reflections | 31531 | 108997 |
| $R_{work}$ / $R_{free}$ | 0.21 / 0.24 | 0.18 / 0.20 |
| No. atoms | | |
| Protein | 3373 | 3388 |
| Ligand | 86 | 58 |
| Water | 284 | 341 |
| *B*-factors | | |
| Protein | 40.99 | 20.53 |
| Ligand | 61.70 | 37.01 |
| Water | 53.11 | 43.17 |
| R.m.s. deviations | | |
| Bond lengths (Å) | 0.015 | 0.012 |
| Bond angles ([illegible]) | 1.9 | 1.6 |

*Data was collected from one crystal. *Hydrogen atoms were excluded from calculations.

**Supplementary Table 15. Data collection and refinement statistics:** PDB entry **7QO2.**

| **Data collection** | **7QO2** |
|---|---|
| Space group | P 43 21 2 |
| Cell dimensions□□ | |
| *a*, *b*, *c* (Å) | 94.28, 94.28, 126.76 |
| □□□□□□□□□□□□ (□) | 90.00 90.00 90.00 |
| Resolution (Å) | 47.14 – 1.77 (1.88 – 1.77) |
| $R_{sym}$ or $R_{merge}$ | 0.11 (1.03) |
| *I* / □*I* | 15.87 (1.94) |
| Completeness (%) | 99.8 (99.0) |
| Redundancy | 7.7 (7.5) |
| | |
| **Refinement** | |
| Resolution (Å) | 47.14 – 1.77 |
| No. reflections | 56230 |
| $R_{work}$ / $R_{free}$ | 0.18 / 0.20 |
| No. atoms | |
| Protein | 3385 |
| Ligand | 52 |
| Water | 401 |
| *B*-factors | |
| Protein | 25.13 |
| Ligand | 65.10 |
| Water | 45.28 |
| R.m.s. deviations | |
| Bond lengths (Å) | 0.018 |
| Bond angles (□) | 1.8 |

*Data was collected from one crystal. *Hydrogen atoms were excluded from calculations.

**Supplementary Table 16. Peptide and protein cleavages.**

a. **Unique and shared cleavages of peptides.** Unique cleavages were performed by only one cathepsin, whereas shared cleavages were performed by two or three cathepsins.
b. **Peptide versus protein cleavages.** Cleavage sites identical among peptides and proteins were separated from cleavages observed by one substrate type only.

| **a. Cleavages in numbers** | **Cathepsin K** | **Cathepsin L** | **Cathepsin V** | **Cathepsins K, L, V (together)** |
|---|---|---|---|---|
| Total peptide cleavages | 52 | 51 | 47 | 150 |
| • Unique | 11 (21%) | 4 (8%) | 3 (6%) | 18 (12%) |
| • Shared | 41 (79%) | 47 (92%) | 44 (94%) | 132 (88%) |
| **b. Comparison of peptide and protein cleavages** | **Cathepsin K** | **Cathepsin L** | **Cathepsin V** | **Cathepsins K, L, V (together)** |
| Total cleavages | 51, 29 | 49, 23 | 46, 38 | 146, 90 |
| • Peptides only | 34 (67%) | 30 (61%) | 20 (43%) | 84 (58%) |
| • Proteins only | 12 (41%) | 4 (17%) | 12 (32%) | 28 (31%) |
| • Identical cleavages (peptides, proteins) | 17 (33%, 59%) | 19 (39%, 83%) | 26 (57%, 68%) | 62 (42%, 69%) |

**Supplementary Table 17. Peptide and protein cleavages and predictions.**

The first column of the table (UniProt/Type) contains UniProt code of the protein origin (www.uniprot.org) and cleavage type. There are two types: positional (one cleavage in the sequence) and area (several cleavages in the sequence). The second column specifies whether the cleavages in the sequence in the next three columns (Cathepsin K, Cathepsin L and Cathepsin V) were obtained from protein or peptide analysis or from SVM based prediction. Cleavage sites are marked with arrows (↓). Asterisks (*) at the end of peptide sequences mark peptides with unique peptide cleavage. Protein sequences marked with ([§]) had no observed protein cleavage sites. The sequences marked with ([&]) were not selected from cleaved protein sequences. Indices 1 and 2 refer to one or two separated cleavage areas in the originated protein, respectively, where cathepsin(s) acted. Indices 3 and 4 mark where there were only one or two cleavage sites in the originated protein, respectively.

| UniProt /Type | Substrate form | Cathepsin K | Cathepsin L | Cathepsin V |
|---|---|---|---|---|
| P42677 Area[1] | Peptide | K D L L↓H P S P | K D L↓L↓H P S P* | K D L L↓H P S P |
| | Protein | K D L L H P S P§ | K D L L H P S P§ | K D L L↓H P S P |
| | Prediction | K D L L H P S P | K D L L H P S P | K D L L H P S P |
| Q6NWY9 Area[2] | Peptide | K P K↓K K T K | K P K↓K↓K T K | K P K↓K↓K T K |
| | Protein | K P↓K K K T K | K P K↓K K T K | K P K↓K K T K |
| | Prediction | K P K↓K↓K T K | K P↓K↓K↓K T K | K P K↓K↓K T K |
| P46777 Area | Peptide | V Y↓E K K P | V Y↓E↓K K P | V Y↓E↓K K P |
| | Protein | V Y↓E K K P | V Y E↓K K P | V Y E↓K K P |
| | Prediction | V Y↓E K K P | V Y↓E↓K K P | V Y↓E↓K K P |
| Q8NC51 Area | Peptide | G A K↓S A A | G A K↓S A A | G A K↓S A A |
| | Protein | G↓A K↓S A↓A | G A K↓S A A | G A K↓S A A |
| | Prediction | G↓A K↓S A↓A | G↓A K S A↓A | G↓A K S A↓A |
| P27695 Area | Peptide | E I D L R↓N P K↓G N | E I D L↓R↓N P K↓G N* | E I D↓L R↓N P K G N* |
| | Protein | E I D L R N P K↓G N | E I D L R↓N P K↓G N | E I D L R↓N P K G↓N |
| | Prediction | E I D L R↓N P K↓G N | E I D L R↓N P K G N | E I D L R↓N P K↓G↓N |
| P16402 Area | Peptide | P V K↓K K A K | P V K↓K K A K | P V K↓K K A K |
| | Protein | P V K↓K K A K | P V K K K A K§ | P V K↓K↓K↓A K |
| | Prediction | P V K↓K↓K↓A K | P V K↓K↓K↓A K | P V K↓K↓K↓A K |
| Q15651 Area | Peptide | R L S↓A K P | R L S↓A K P | R L↓S↓A K P* |
| | Protein | R L S↓A K P | R L S↓A K P | R L S↓A↓K P |
| | Prediction | R↓L S↓A K P | R↓L S↓A↓K P | R↓L↓S↓A K P |
| A6NHL2 Area | Peptide | L L↓S↓G K E | L L↓S↓G K E | L L↓S↓G K E |
| | Protein | L L S↓G↓K↓E | L L S↓G↓K E | L L S↓G↓K E |
| | Prediction | L L↓S↓G↓K↓E | L L↓S↓G↓K↓E | L L↓S↓G↓K E |
| Q6S8J3, P60709, P63261, A5A3E0, P0CG38, P0CG39 Area | Peptide | G M C↓K A G | G M C↓K↓A G* | G M C↓K A G |
| | Protein | G M C↓K↓A G | G M C K↓A G | G M C↓K↓A G |
| | Prediction | G M C↓K↓A G | G M C K↓A G | G M C↓K↓A G |
| P14625 | Peptide | K↓R↓F Q↓N↓V A↓K* | K R↓F↓Q↓N V A↓K | K↓R↓F↓Q↓N V A↓K |

| | | | | |
|---|---|---|---|---|
| Area | Protein | K R F Q↓N V A↓K | K R F Q N V A↓K | K R F Q↓N V A↓K |
| | Prediction | K R F Q↓N↓V A↓K | K R F Q↓N V A↓K | K R F↓Q↓N↓V A↓K |
| P63104 Area | Peptide | A C M↓K S V T↓E | A C M↓K S V T↓E | A C M↓K S V T↓E |
| | Protein | A C M K↓S V↓T↓E | A C M↓K S V T↓E | A C M↓K↓S V T↓E |
| | Prediction | A C M↓K↓S V T↓E | A C M↓K↓S V T↓E | A C M↓K↓S V T↓E |
| P25205 Area | Peptide | A Y F K↓K V L | A Y F↓K↓K V L | A Y F↓K K V L |
| | Protein | A↓Y F K↓K V L | A Y F K↓K V L | A Y F↓K↓K V L |
| | Prediction | A↓Y F K↓K V L | A↓Y F↓K↓K V L | A Y F↓K↓K V L |
| Q02878 Area | Peptide | K V L↓A↓T V T↓K | K V L↓A T V T↓K | K V L↓A T V T↓K |
| | Protein | K V L A↓T↓V T↓K | K V L A T V T K§ | K V L A↓T V T↓K |
| | Prediction | K V L↓A↓T V T↓K | K V L↓A↓T↓V T↓K | K V L↓A↓T↓V T↓K |
| Q92772 Positional[3] | Peptide | E V C↓K↓K K K | E V C↓K↓K K K | E V C↓K K K K |
| | Protein | E V C↓K K K K | E V C↓K K K K | E V C↓K K K K |
| | Prediction | E V C↓K K K K | E V C↓K↓K K K | E V C↓K↓K K K |
| P07199 Positional[3] | Peptide | I I L↓K↓E K | I I L↓K↓E K | I I L↓K↓E K |
| | Protein | I I L↓K E K | I I L↓K E K | I I L↓K E K |
| | Prediction | I I L↓K↓E K | I I L↓K↓E K | I I L↓K↓E K |
| Q8N5V2 Positional[3] | Peptide | T R↓E S E D L E | T R↓E↓S↓E D L E | T R↓E↓S↓E D L E |
| | Protein | T R E S↓E D L E | T R E S↓E D L E | T R E S↓E D L E |
| | Prediction | T R↓E S E D L E | T R E S E D L E | T R E S E D L E |
| Q53FA7 Positional[3] | Peptide | V L↓L K↓V A A S | V L↓L K V A A S | V L↓L K V A A S |
| | Protein | V L L K V A A S§ | V L L K↓V A A S | V L L K↓V A A S |
| | Prediction | V L↓L K↓V A↓A S | V L↓L K↓V A↓A S | V L L K↓V A↓A S |
| Q9C0B0 Positional[4] | Peptide | A W↓K↓K↓E A* | A W K↓K E A | A W K↓K E A |
| | Protein | A W K K E A§ | A W K↓K E A | A W K↓K E A |
| | Prediction | A W K↓K↓E A | A W K↓K E A | A W↓K↓K E A |
| O43818 Positional[4] | Peptide | Q L↓R↓Q Q E | Q L↓R↓Q Q E | Q L↓R↓Q Q E |
| | Protein | Q L R↓Q Q E | Q L R↓Q Q E | Q L R↓Q Q E |
| | Prediction | Q L R↓Q Q E | Q L R↓Q Q E | Q L R↓Q Q E |
| O43823 positional | Peptide | V P C↓G T A H E* | V P C G↓T A H E | V P C G↓T A H E |
| | Protein | V P C G T A H E§ | V P C G T A H E§ | V P C G↓T A H E |
| | Prediction | V P C G↓T A H E | V P C G T A H E | V P C G T A H E |
| O75533 Positional | Peptide | K I A↓K T H | K I A↓K T H | K I A↓K T H |
| | Protein | K I A K T H§ | K I A K T H§ | K I A↓K T H |
| | Prediction | K I A↓K T H | K I A↓K T H | K I A↓K T H |
| P46013 Positional | Peptide | V A↓C K S S Q P | V A↓C K S S Q P | V A↓C K S S Q P |
| | Protein | V A C K↓S S Q P | V A C K↓S S Q P | V A C K↓S S Q P |
| | Prediction | V A↓C K↓S S Q P | V A↓C K↓S S Q P | V A↓C K↓S S Q P |
| P42704 Positional | Peptide | G N Y K↓E↓A↓K K* | G N Y K↓E A K K | G N Y K↓E A K K |
| | Protein | G N Y K E A K K§ | G N Y K E A K K§ | G N Y K↓E A K K |
| | Prediction | G N Y K↓E A K↓K | G N Y K↓E A K K | G N Y K↓E↓A K↓K |
| Q9Y490 | Peptide | Q L L↓V↓A↓C↓K V K* | Q L L↓V A C↓K V K | Q L L V A C↓K V K |

| | | | | |
|---|---|---|---|---|
| Positional | Protein | Q L L V A C K V K[§] | Q L L V↓A C K V K | Q L L V↓A C K V K |
| | Prediction | Q L L↓V A C↓K↓V K | Q L L↓V↓A↓C↓K↓V K | Q L L↓V A↓C↓K↓V K |
| P25398 Positional | Peptide | R G↓I R↓E A A K | R G↓I R↓E A A K | R G↓I R↓E A A K |
| | Protein | R G I R↓E A A K | R G I R↓E A A K | R G I R↓E A A K |
| | Prediction | R G I R↓E A A K | R G I R↓E↓A A K | R G I R↓E↓A A K |
| Q92747 Positional | Peptide | S I Y↓E V D↓K Q* | S I Y↓E↓V D K Q | S I Y↓E↓V D K Q |
| | Protein | S I Y E V D K Q[§] | S I Y E V D K Q[§] | S I Y E↓V D K Q |
| | Prediction | S I Y↓E V D K Q | S I Y↓E↓V D K Q | S I Y↓E↓V D↓K Q |
| P62906 Positional | Peptide | K K Y D↓A F↓L↓A* | K K Y D↓A F L↓A | K K Y↓D↓A F L↓A* |
| | Protein | K K Y D A F L A[§] | K K Y D↓A F L A | K K Y D↓A F L A |
| | Prediction | K K Y D A F↓L↓A | K K Y D↓A F L↓A | K K Y D↓A F L↓A |
| | Peptide | L L↓K A V A E K Q | L L↓K↓A V A E K Q | L L↓K A V A E K Q |
| | Protein[&] | No data | No data | No data |
| | Prediction | L L↓K↓A V A E K Q[§] | L L↓K↓A V A E K Q[§] | L L↓K↓A V A E K Q[§] |

**Supplementary Data 1. Input data – individual data sets of peptides for cathepsins K, V, B, L, S, and F.**

Tables are provided in a separate excel file (SuppData1_Cat_KVBLSF.xlsx).

**Supplementary Data 2. Specific 941 cleavages – only one cleavage in the whole protein.**

Table is provided as separate excel file (SuppData2_941proteins.xlsx).

**Supplementary Data 3.** **SVM models for predictions which can be used as input for PCSS server (https://salilab.org).**

SVM models are provided in a separate ascii files for cathepsins K, V, B, L, S, and F:

SuppData3_CatK_SVMmodel.txt;
SuppData3_CatV_SVMmodel.txt;
SuppData3_CatB_SVMmodel.txt;
SuppData3_CatL_SVMmodel.txt;
SuppData3_CatS_SVMmodel.txt;
SuppData3_CatF_SVMmodel.txt;

in one “zip” file called “SuppData3_Cat_BFKLSV_SVMmodels.zip”.

Short guidelines for using developed SVM models:

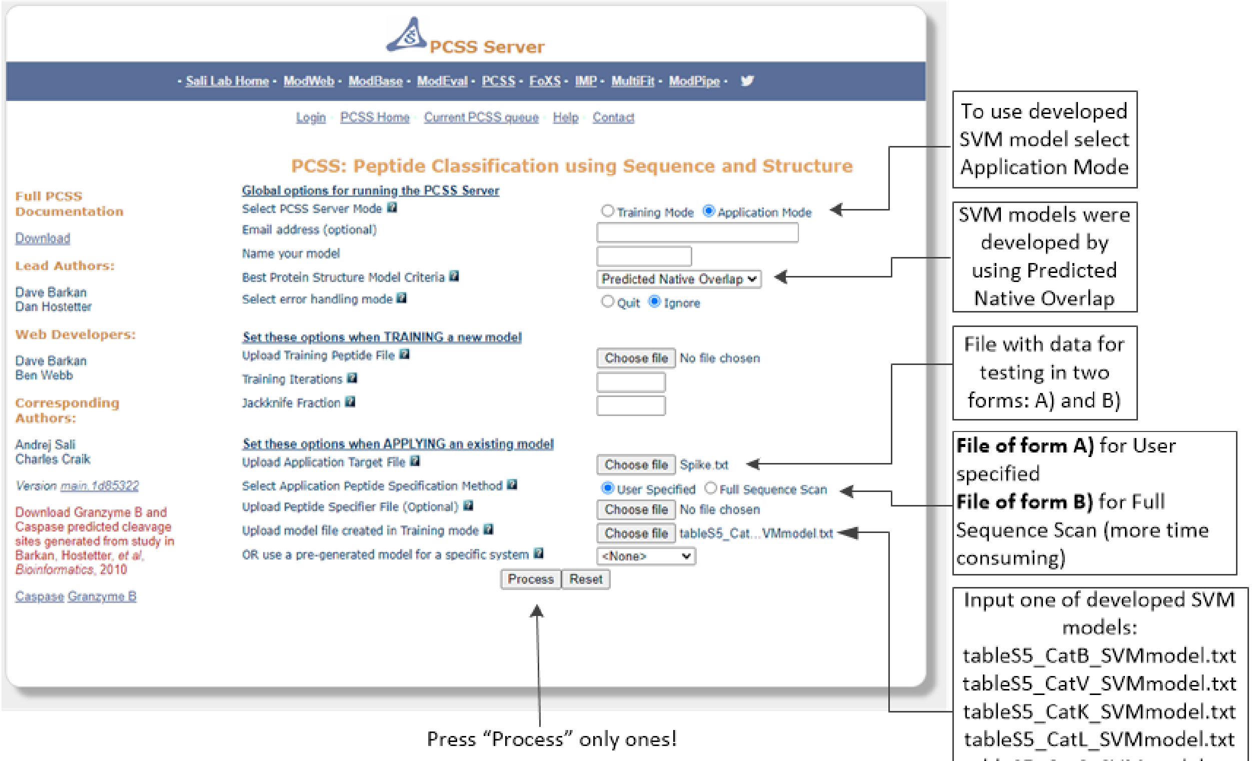


**File of form A)** Spike.txt (Legend: UniProtNo,P4, P4-P4',Positive or Negative Peptide; Only red characters are input and they must be written in file)
P0DTC2 682 RRARSVAS Positive
P0DTC2 692 IIAYTMSL Positive
P0DTC2 812 PSKRSFIE Positive
P0DTC2 676 TQTNSPRR Positive
P0DTC2 680 SPRRARSV Positive

**File of form B)** xxx.txt (Legend: UniProtNo; Only red characters are input and they must be written in file)
P0DTC2

**Supplementary Data 4. Peptide fragment identification.**

a. **Peptides treated with cathepsins were analyzed using reverse phase HPLC**. Peaks representing the peptide fragments were captured. Separation was monitored at 214 nm. The y-axis shows normalized signal of absorbance at A214 and the x-axis shows separation time in minutes. Peptide sequences as identified using MALDI-TOF are written on top or next to their corresponding HPLC signals. Characteristic signals at approximately 8 and 12 min correspond to buffer component dithiothreitol (DTT) and cathepsin, respectively.
b. **Processing of peptides AYFKKVL and KVLATVTK from 5 s–60 min.** Aliquots were taken after 5 s, 30 s, 2 min, 6 min, 20 min, and 60 min of incubation with cathepsins V and L (both peptides) or K (peptide KVLATVTK). Response at Y-axis is not normalized to quantitatively compare fragment signals at different time points.

HPLC spectra are provided as separate file (SuppData4.pdf).

**Supplementary Note 1. Comparison of peptide and protein cleavages by cathepsins V, L and K.**

After treatment with native cathepsins V, L, and K, we determined the cleavage sites of 28 peptides with N- and C-terminal protections (peptide p15 was used in the structural assay). In total, 150 cleavages were observed. Supplementary Table 16 shows that 42% of all peptide cleavages and 69% of all protein cleavages were identical, whereas the remaining cleavages were observed only with one type of substrate (58% of total peptide and 31% of total protein cleavages). Most cleavages were shared (performed by more than one cathepsin), whereas a few were unique to only one cathepsin (Supplementary Table 16a). Statistical comparison of the patterns of cleaved peptides and their protein counterparts showed that there was no significant difference between their cleavage patterns, demonstrating that the selected sequences indeed represented a variety of protein cleavage samples of all seven cathepsin V clusters, despite those peptides were cleaved in more places than their protein counterparts (146 peptide and 90 protein cleavages among the selected sequences; Supplementary Table 16b).

Of all 28 sequences treated with three different cathepsins, only 11 were cleaved into peptides and proteins at the same position by at least one cathepsin. Other sequences contained additional cleavages that was observed with only one substrate type. In contrast, sequences EVC↓KKKK, IIL↓KEK, and TRES↓EDLE had only one observed protein cleavage site, indicating very restrictive processing, whereas in peptides they were cleaved at two sites by all three cathepsins (IIL↓K↓EK), at two sites by cathepsins K and L (EVC↓K↓KKK), and at three sites by cathepsins V and L (TR↓E↓S↓EDLE). In addition, several sequences were not cleaved in proteins by cathepsin K, L, or both, whereas they cleaved each sequence at least at one site in the peptidyl form. Four of these sequences (AWKKEA, SIYEVDKQ, KKYDAFLA, and GNYKEAKK) appeared as weak substrates of cathepsin K in the peptidyl form and were only partially processed by cathepsin K during the incubation period. We also observed multiple fragments that had in their sequences embedded protein cleavage sites, which were evidently not cleaved when present in peptides. These were ESEDLE, ATVT, and KPK fragments with intact protein cleavage sites TRES↓EDLE, KVLAT↓VTK, KP↓KKKTK (cathepsin K), NPKGN, and AKP with cleavage sites EIDLRNPKG↓N and RLSA↓KP (cathepsin V), and KSVT with cleavage sites ACMK↓SVTE (cathepsins V and K) and ACMKSV↓TE (cathepsin K) (Supplementary Table 17, Supplementary Data 4a). These data indicated that the recognition of several sequences in proteins and peptides is not the same.

Interestingly, we discovered that cathepsins cleaved peptides along the entire length of peptide sequences, including the terminal residues and their protective groups, four of which had their C-terminal residues removed by all cathepsins, whereas cathepsins K and V cleaved the amino-terminal residue of one peptide each. This discovery suggests the exopeptidase activity of cathepsins toward peptides. To gain further insight, we followed the cleavage of peptides AYFKKVL and KVLATVTK from 5 s to 60 min. The analysis confirmed the carboxypeptidase activity of cathepsins V and L, but not that of K, which is evident from the processing of fragments AYFK and KVLA to AYF and KVL, respectively (Supplementary Data 4b).

**Supplementary References**


68. Li, J. *et al.* Structural basis for DNA recognition by STAT6. *Proc. Natl. Acad. Sci. U. S. A.* (2016) doi:10.1073/pnas.1611228113.
69. Wang, Z., Wu, Y., Li, L. & Su, X. D. Intermolecular recognition revealed by the complex structure of human CLOCK-BMAL1 basic helix-loop-helix domains with E-box DNA. *Cell Res.* (2013) doi:10.1038/cr.2012.170.
70. Lolli, G., Pinna, L. A. & Battistutta, R. Structural determinants of protein kinase CK2 regulation by autoinhibitory polymerization. *ACS Chem. Biol.* (2012) doi:10.1021/cb300054n.
71. Millet, J. K. & Whittaker, G. R. Host cell proteases: Critical determinants of coronavirus tropism and pathogenesis. *Virus Res.* (2015) doi:10.1016/j.virusres.2014.11.021.
