# Supplementary material for: Proteomic data and structure analysis combined reveal interplay of structural rigidity and flexibility on selectivity of cysteine cathepsins
Source: Commun Biol. 2023 Apr 24;6:450. doi: 10.1038/s42003-023-04772-8 (PMC10124925; doi:10.1038/s42003-023-04772-8)
Supplement: Supplementary file 5 — Supplementary Data 4 [file 42003_2023_4772_MOESM5_ESM.pdf]

Supplementary Data 4. Peptide cleavage analysis.

a. Peptide fragment identification. Peptides treated with cathepsins were applied on RP-HPLC. Peaks representing the peptide fragments were captured. Separation was monitored at 214 nm. Y-axis show normalized signal of absorbance at A_{214} and x-axis represent separation time in minutes. Peptide sequences as identified with MALDI-TOF are written on top or next to their corresponding HPLC signal. Characteristics signals at around 8 min and 12 min correspond to buffer component dithiotheritrol (DTT) and cathepsin, respectively.

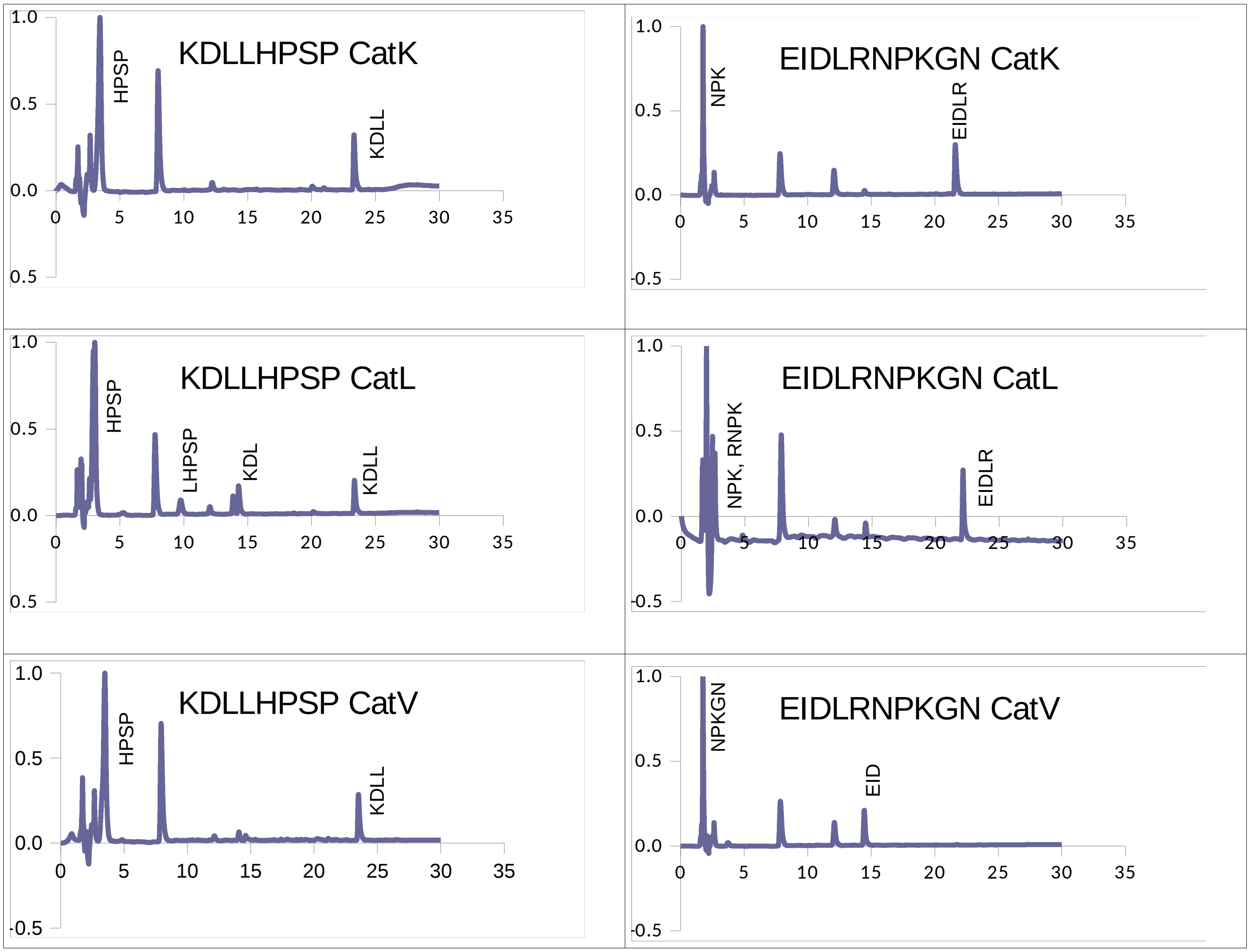

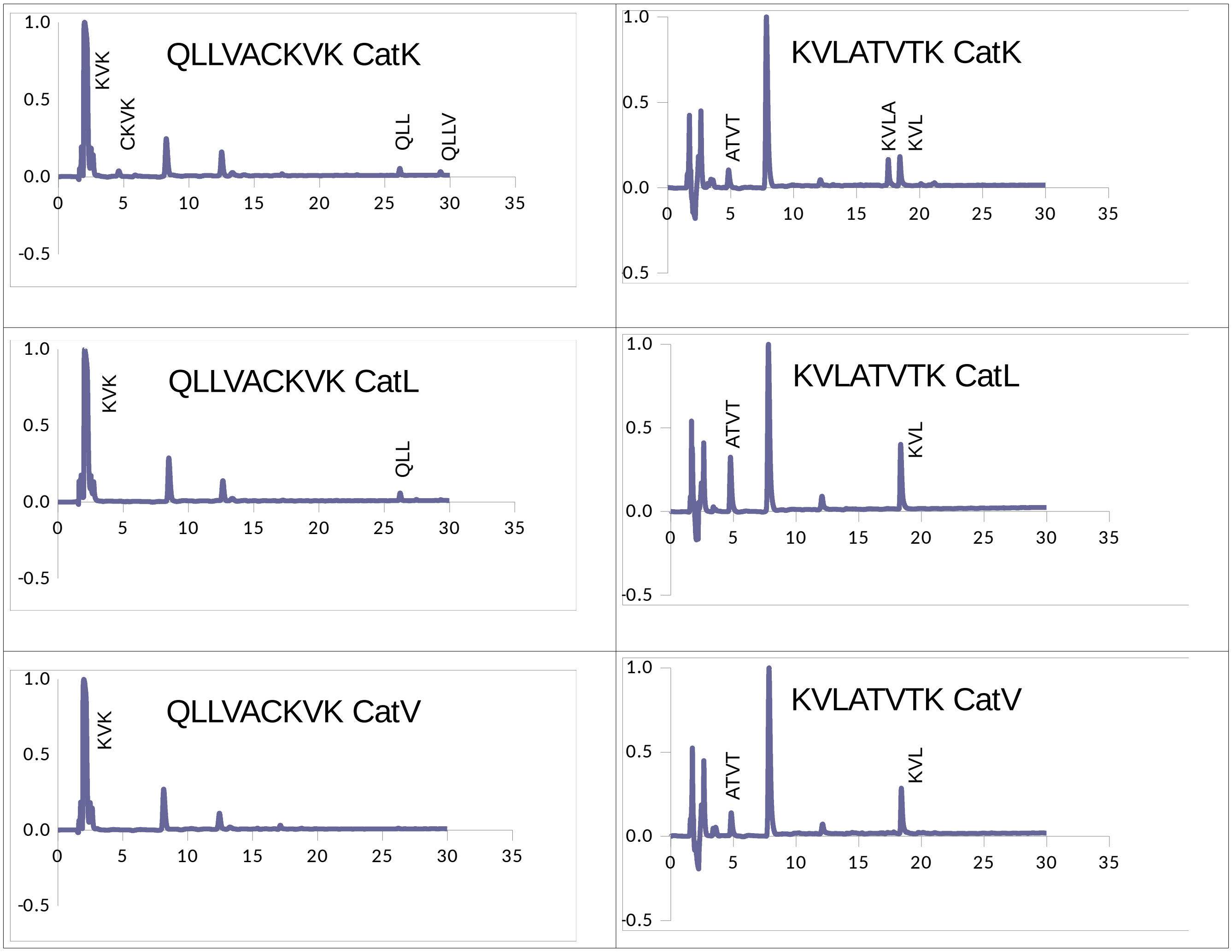
QLLVACKVK CatK
KVK
CKVK
QLL
QLLV
KVLATVTK CatK
ATVT
KVLA
KVL
QLLVACKVK CatL
KVK
QLL
KVLATVTK CatL
ATVT
KVL
QLLVACKVK CatV
KVK
KVLATVTK CatV
ATVT
KVL

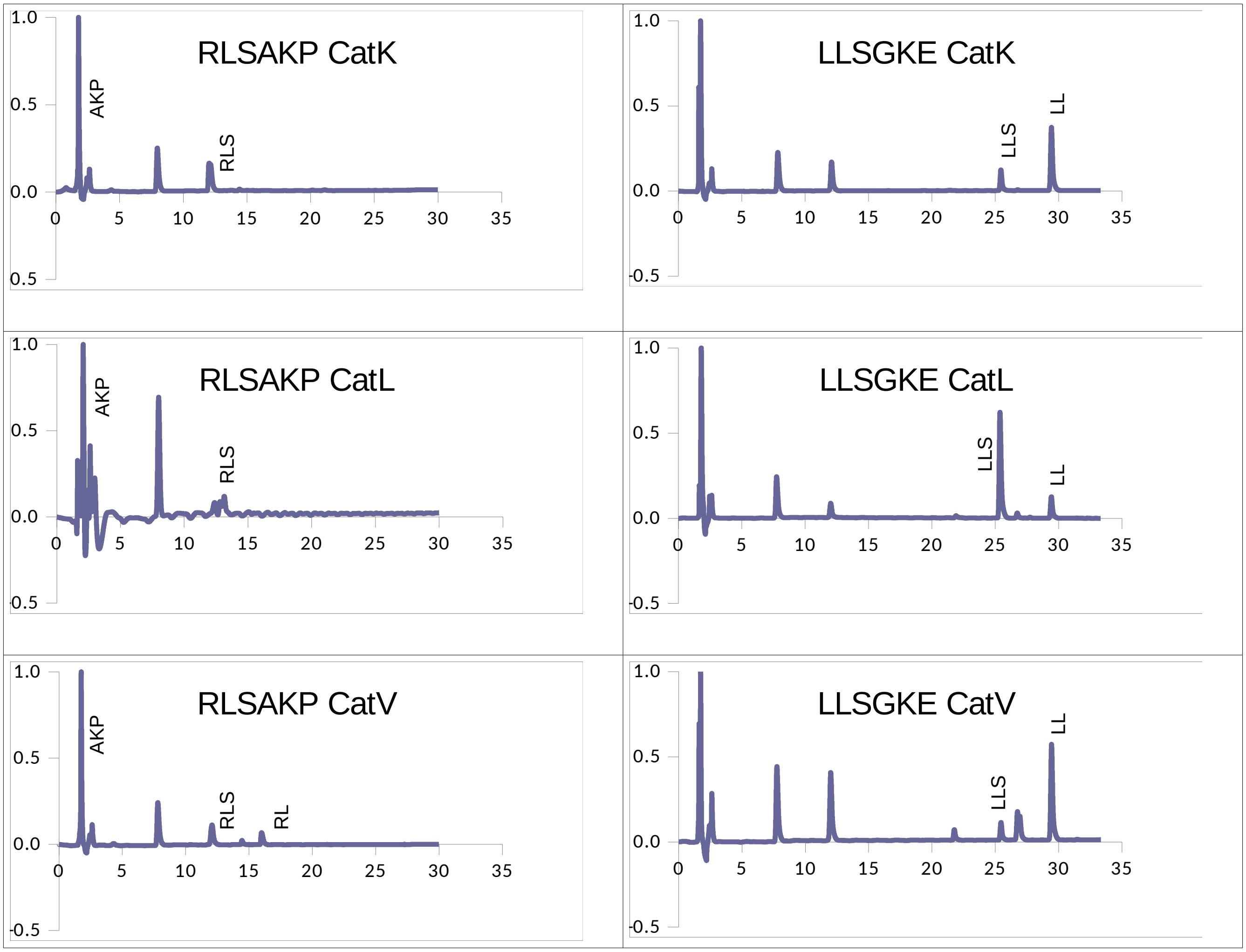
RLSAKP CatK
AKP
RLS
LLSGKE CatK
LLS
LL
RLSAKP CatL
AKP
RLS
LLSGKE CatL
LLS
LL
RLSAKP CatV
AKP
RLS
RL
LLSGKE CatV
LLS
LL

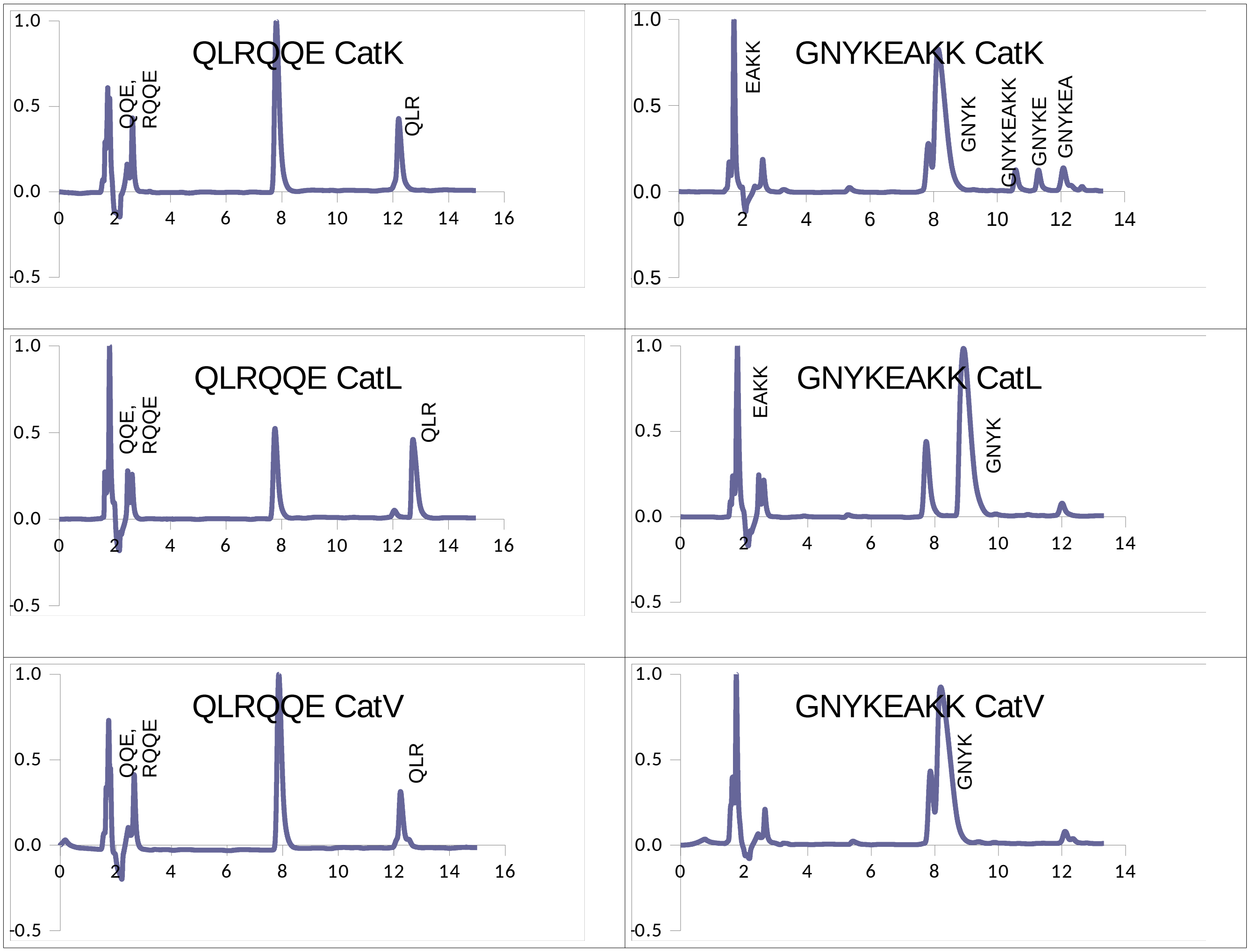
QLRQQE CatK
QQE, RQQE
QLR
GNYKEAKK CatK
EAKK
GNYK
GNYKEAKK
GNYKE
GNYKEA
QLRQQE CatL
QQE, RQQE
QLR
GNYKEAKK CatL
EAKK
GNYK
QLRQQE CatV
QQE, RQQE
QLR
GNYKEAKK CatV
GNYK

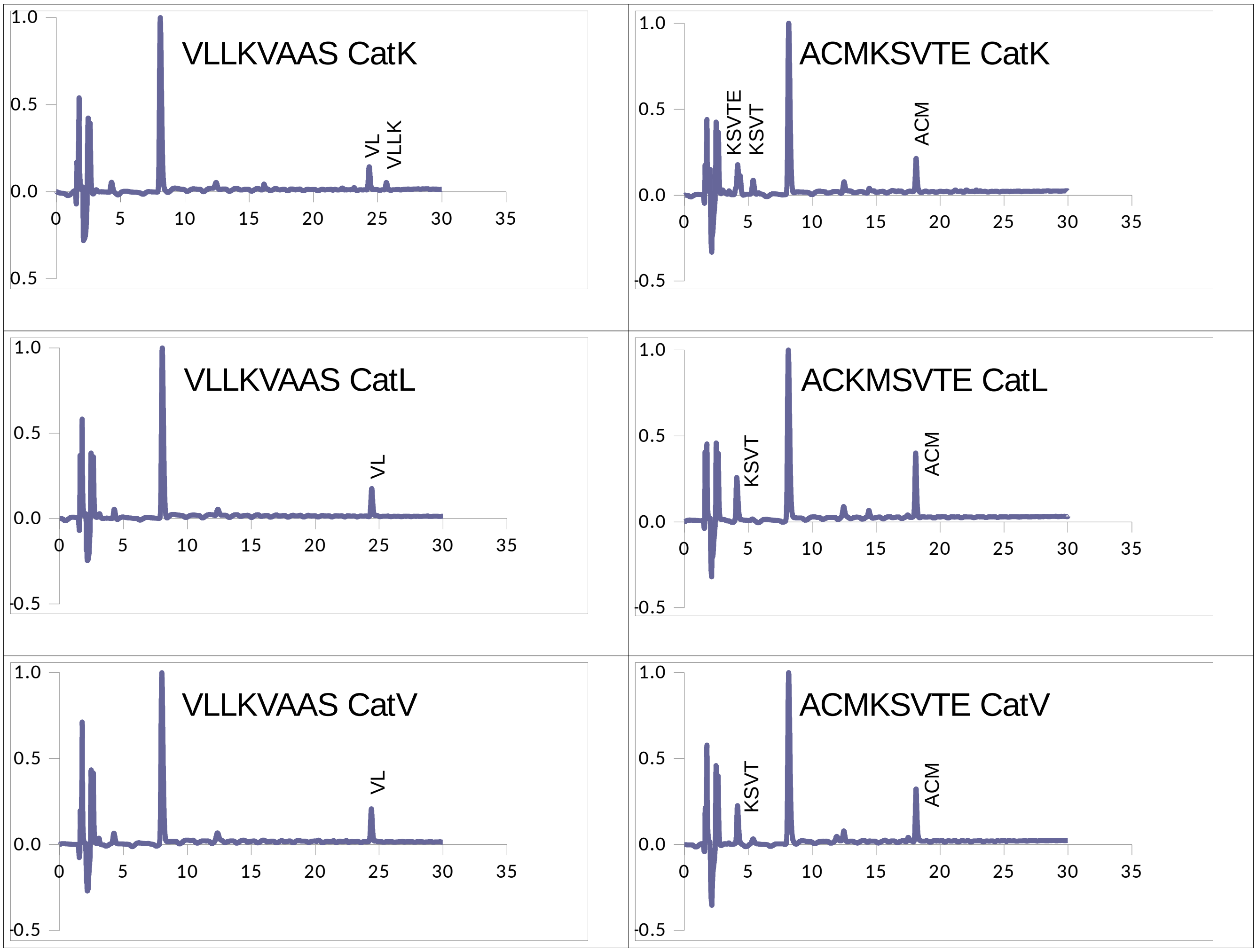

VLLKVAAS CatK
VL
VLLK
ACMKSVTE CatK
KSVTE
KSVT
ACM
VLLKVAAS CatL
VL
ACKMSVTE CatL
KSVT
ACM
VLLKVAAS CatV
VL
ACMKSVTE CatV
KSVT
ACM

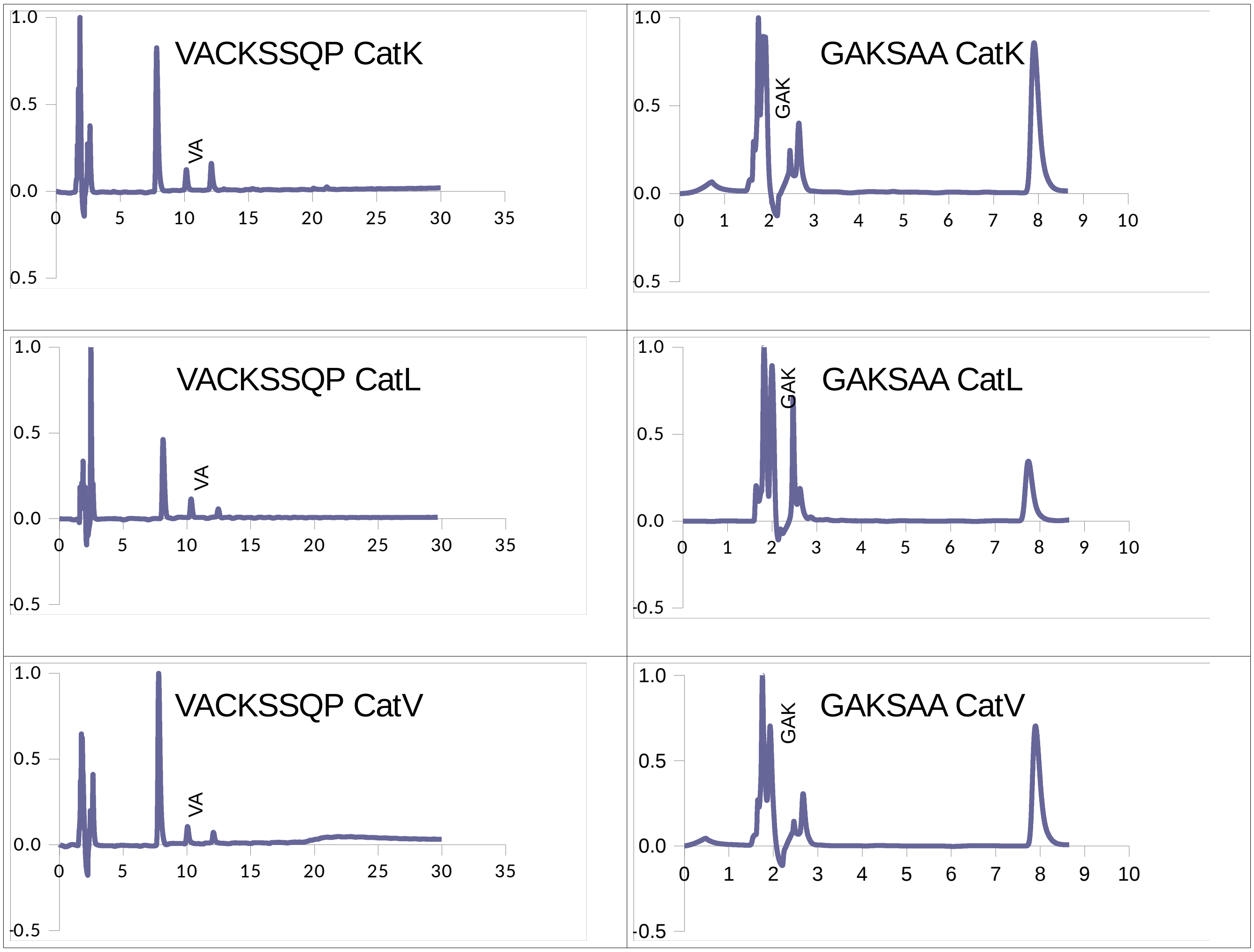
VACKSSQP CatK
VA
GAKSAA CatK
GAK
VACKSSQP CatL
VA
GAKSAA CatL
GAK
VACKSSQP CatV
VA
GAKSAA CatV
GAK
1.0
0.5
0.0
-0.5
0
5
10
15
20
25
30
35
0
1
2
3
4
5
6
7
8
9
10

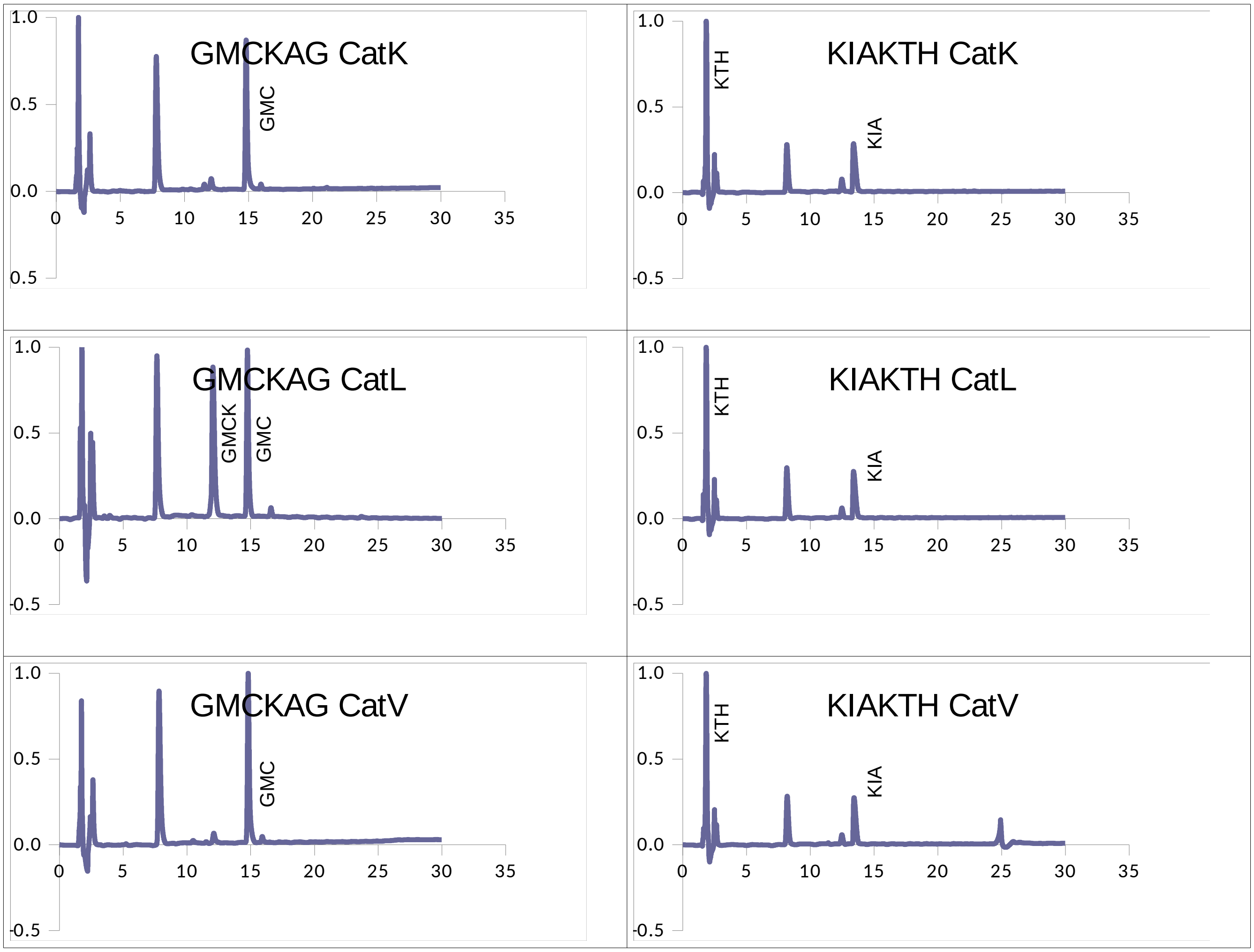
GMCKAG CatK
GMC
KIAKTH CatK
KTH
KIA
GMCKAG CatL
GMCK
GMC
KIAKTH CatL
KTH
KIA
GMCKAG CatV
GMC
KIAKTH CatV
KTH
KIA
1.0
0.5
0.0
-0.5
0
5
10
15
20
25
30
35

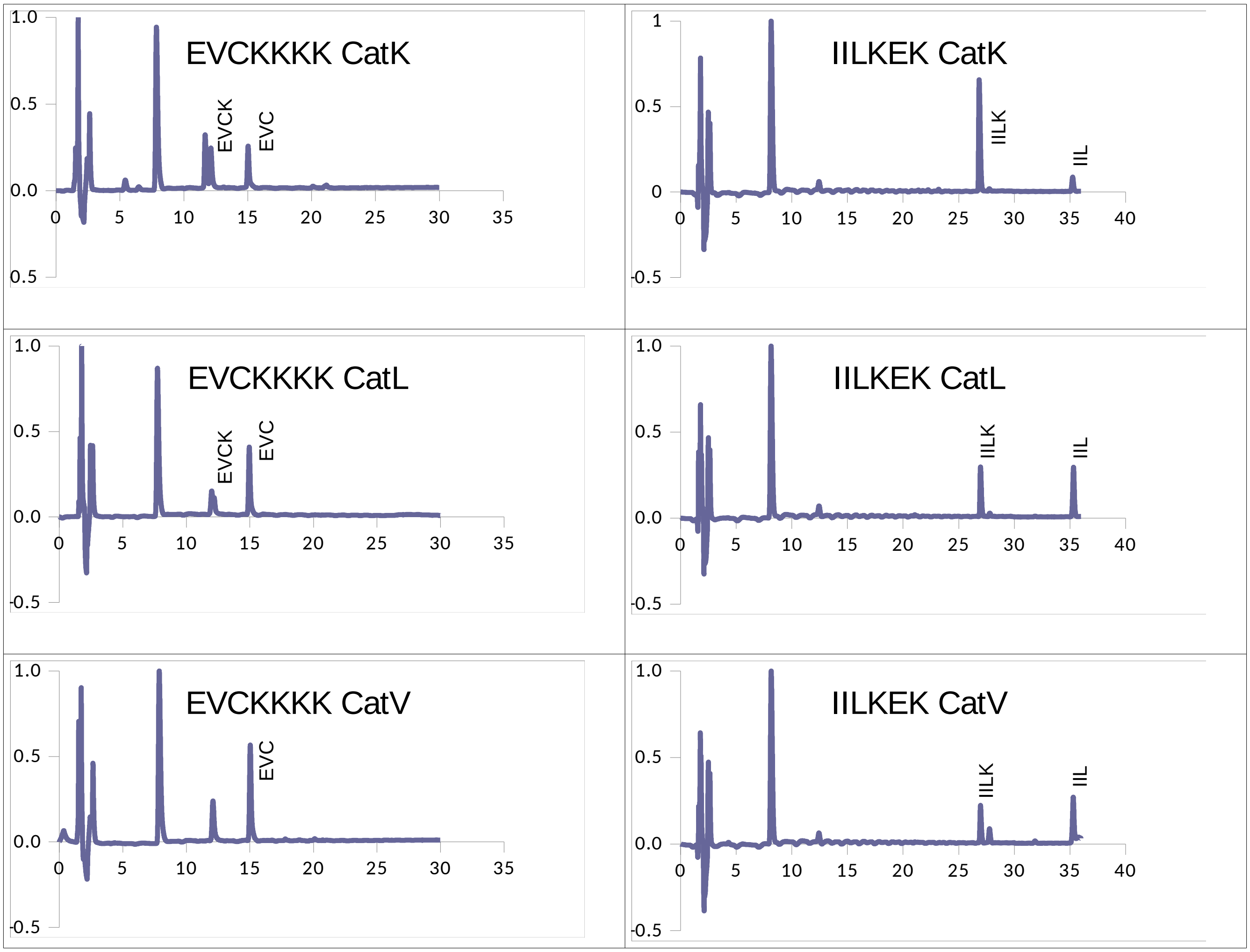
EVCKKKK CatK
EVCK
EVC
IILKEK CatK
IILK
IIL
EVCKKKK CatL
EVCK
EVC
IILKEK CatL
IILK
IIL
EVCKKKK CatV
EVC
IILKEK CatV
IILK
IIL
1.0
0.5
0.0
-0.5
0 5 10 15 20 25 30 35 40

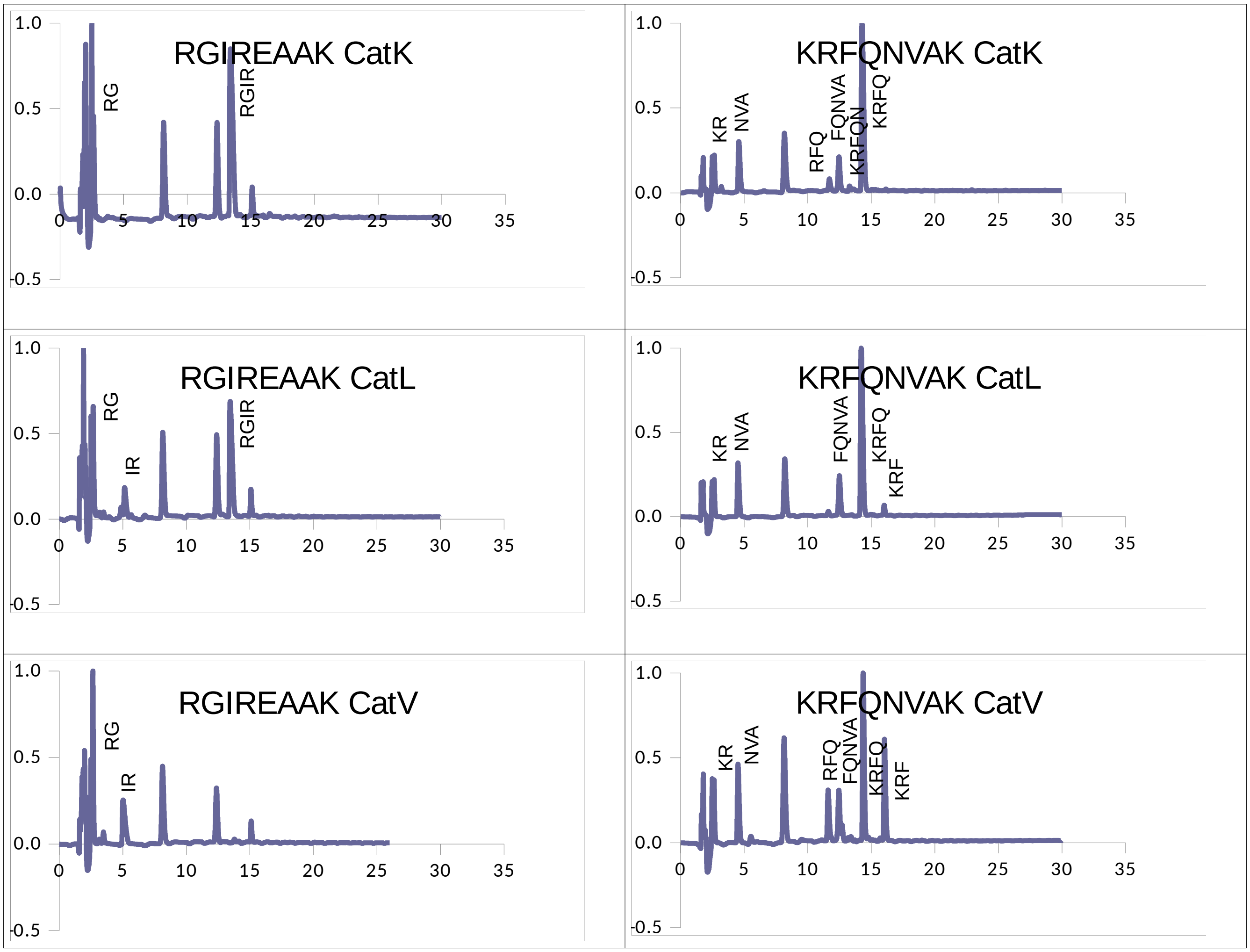

RGIREAAK CatK
RG
RGIR
KRFQNVAK CatK
KR
NVA
RFQ
FQNVA
KRFQN
KRFQ
RGIREAAK CatL
RG
IR
RGIR
KRFQNVAK CatL
KR
NVA
FQNVA
KRFQ
KRF
RGIREAAK CatV
RG
IR
KRFQNVAK CatV
KR
NVA
RFQ
FQNVA
KRFQ
KRF

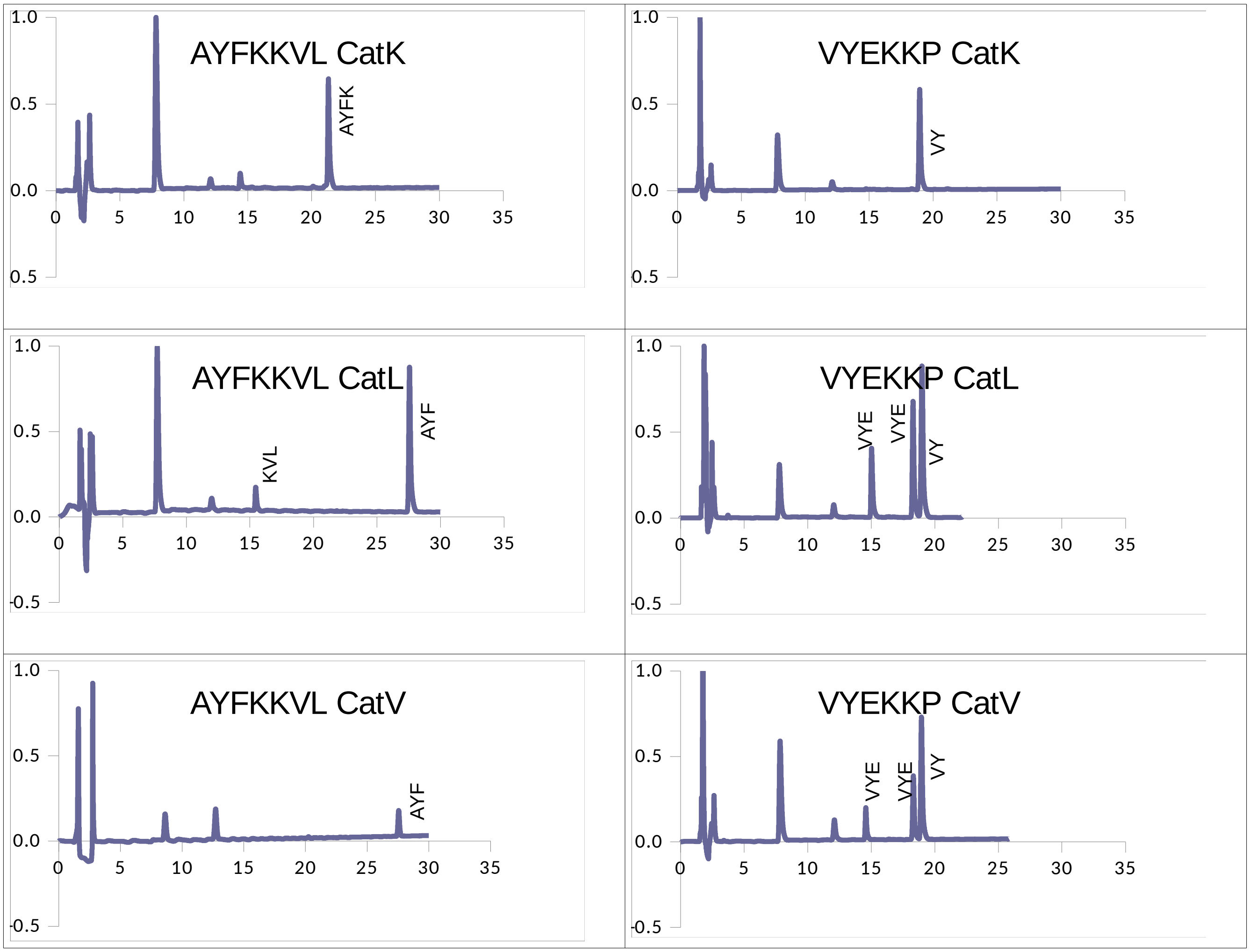
AYFKKVL CatK
AYFK
VYEKKP CatK
VY
AYFKKVL CatL
KVL
AYF
VYEKKP CatL
VYE
VYE
VY
AYFKKVL CatV
AYF
VYEKKP CatV
VYE
VYE
VY
1.0
0.5
0.0
-0.5
0
5
10
15
20
25
30
35

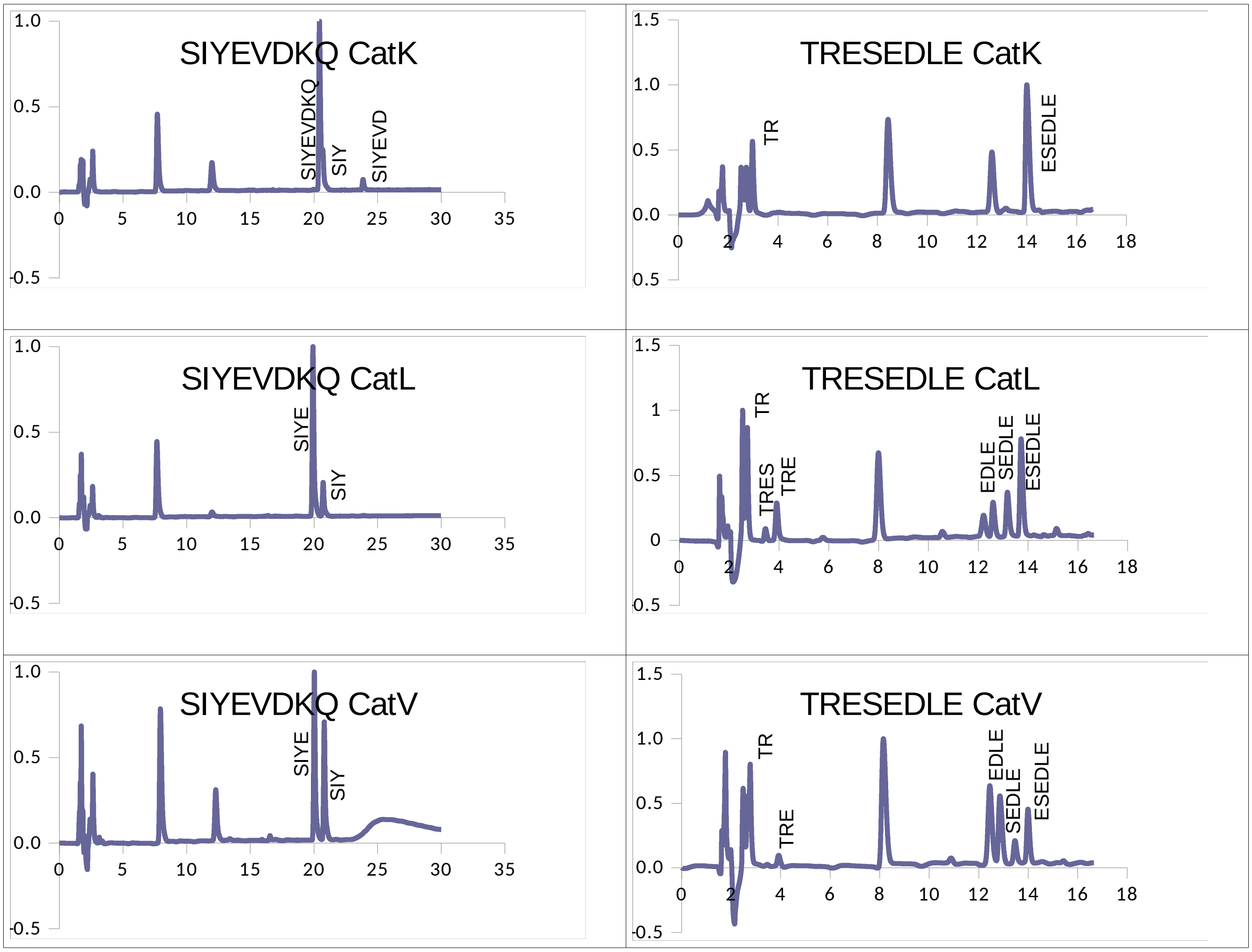

SIYEVDKQ CatK
SIYEVDKQ
SIY
SIYEVD
TRESEDLE CatK
TR
ESEDLE
SIYEVDKQ CatL
SIYE
SIY
TRESEDLE CatL
TR
TRES
TRE
EDLE
SEDLE
ESEDLE
SIYEVDKQ CatV
SIYE
SIY
TRESEDLE CatV
TR
TRE
EDLE
SEDLE
ESEDLE

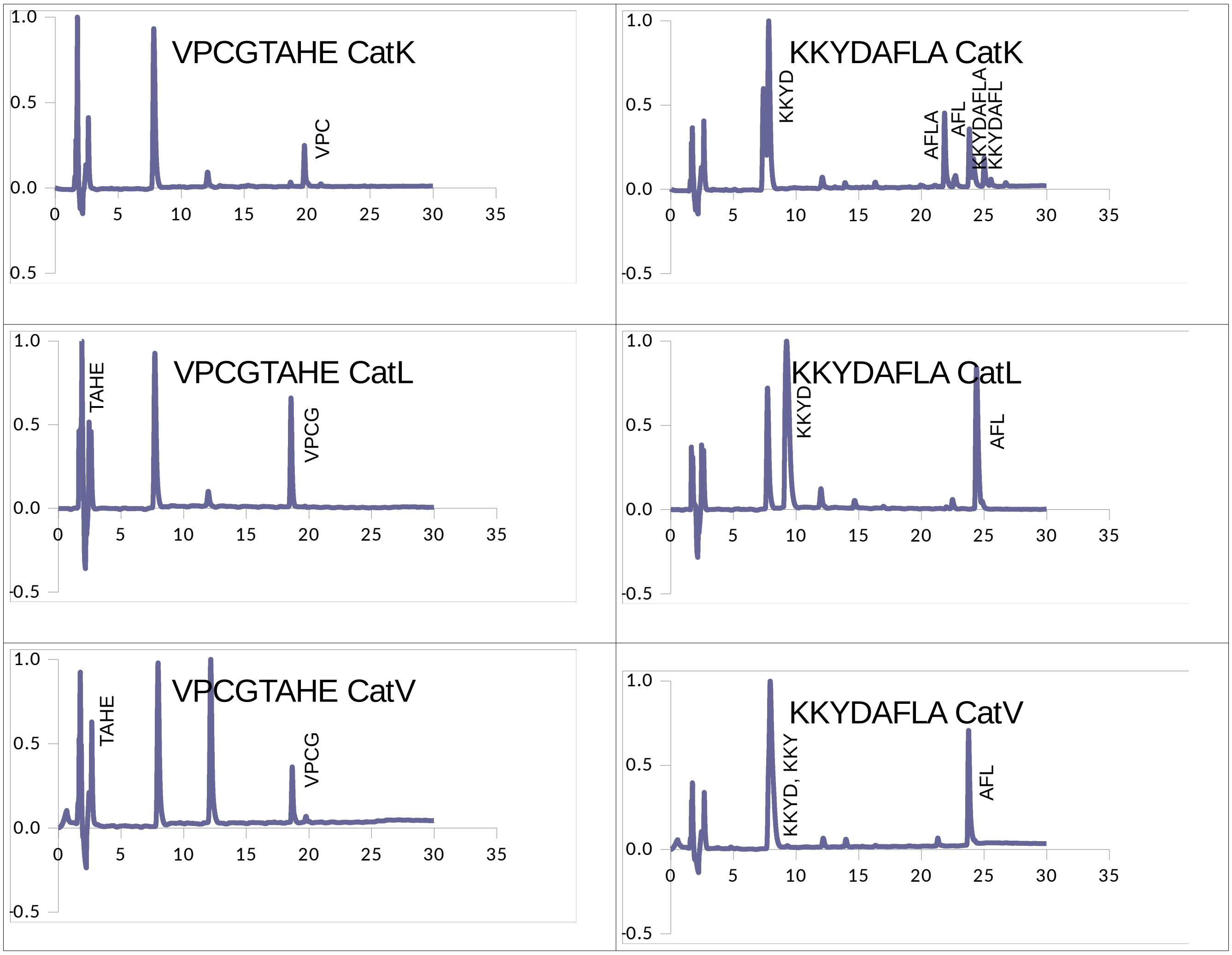
VPCGTAHE CatK
VPC
KKYDAFLA CatK
KKYD
AFLA
AFL
KKYDAFLA
KKYDAFL
VPCGTAHE CatL
TAHE
VPCG
KKYDAFLA CatL
KKYD
AFL
VPCGTAHE CatV
TAHE
VPCG
KKYDAFLA CatV
KKYD, KKY
AFL

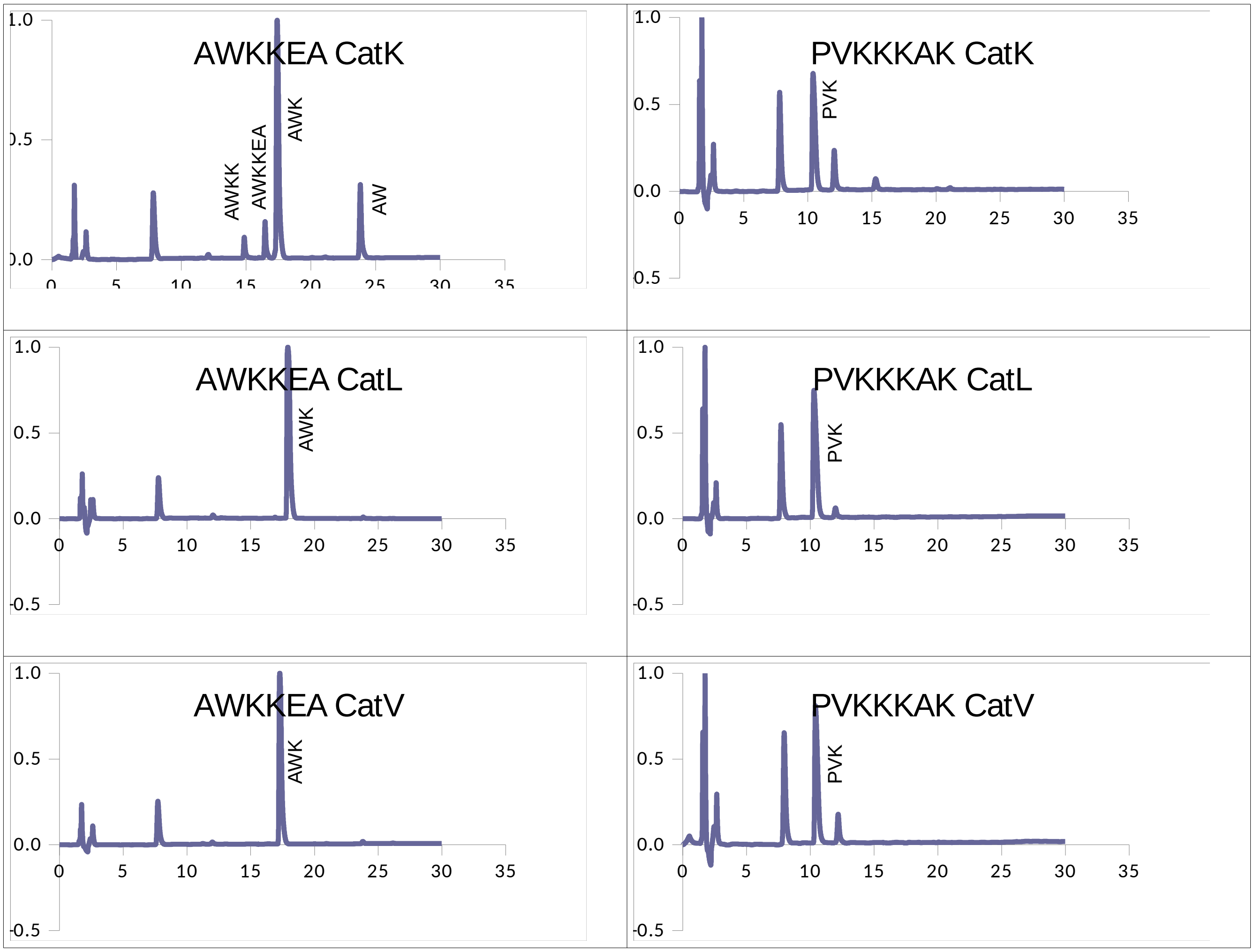

AWKKEA CatK
AWKK
AWKKEA
AWK
AW
PVKKKAK CatK
PVK
AWKKEA CatL
AWK
PVKKKAK CatL
PVK
AWKKEA CatV
AWK
PVKKKAK CatV
PVK
1.0
0.5
0.0
-0.5
0
5
10
15
20
25
30
35

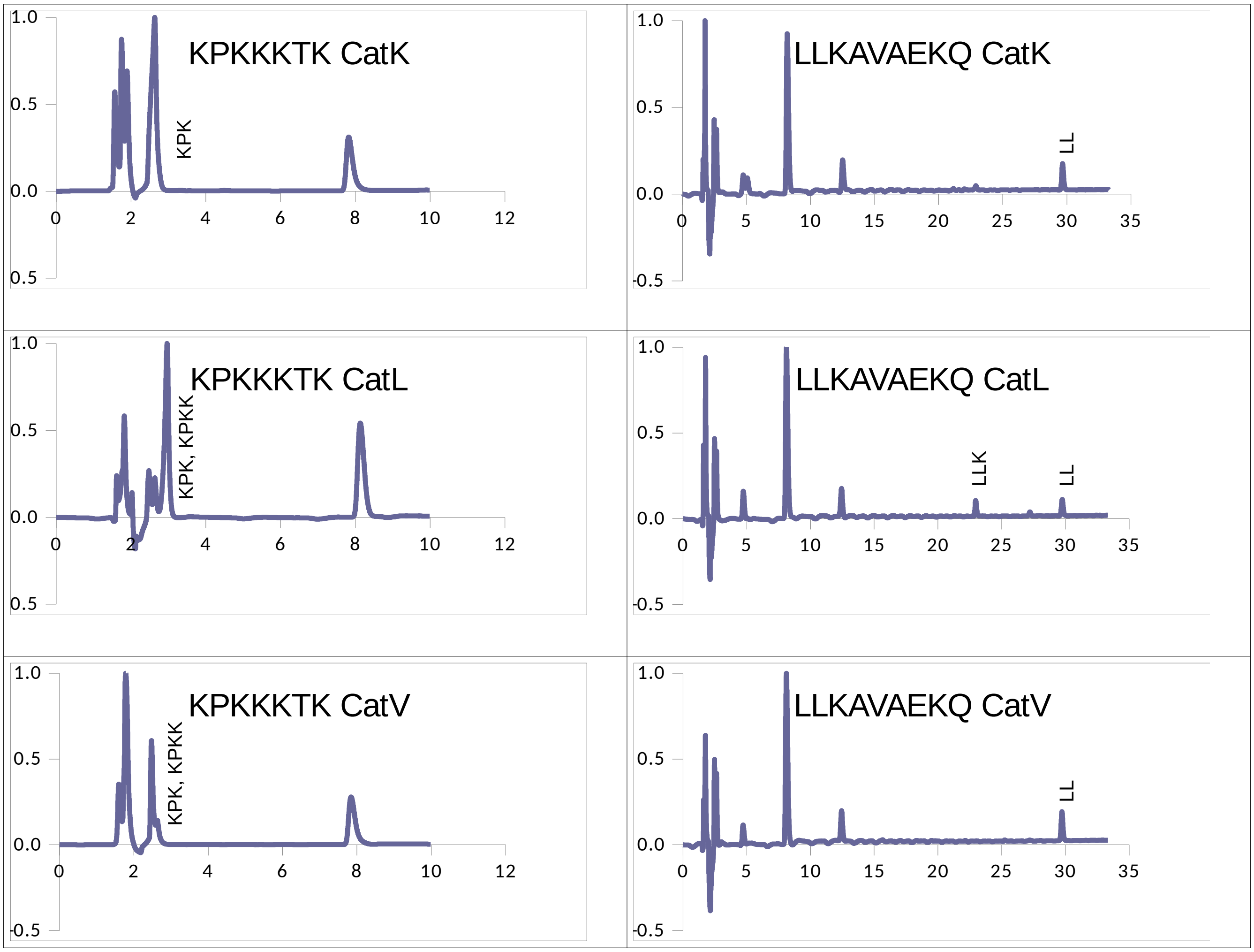
KPKKKTK CatK
KPK
LLKAVAEKQ CatK
LL
KPKKKTK CatL
KPK, KPKK
LLKAVAEKQ CatL
LLK
LL
KPKKKTK CatV
KPK, KPKK
LLKAVAEKQ CatV
LL

b. Processing of peptides AYFKKVL and KVLATVTK from 5 s–60 min. Peptides treated with cathepsins were applied on RP-HPLC. Peaks representing the peptide fragments were captured. Separation was monitored at 214 nm. Y-axis show signal of absorbance at A_{214} and x-axis represent separation time in minutes. Response at Y-axis is not normalized in order to quantitatively compare fragment signals at different time points. Peptide sequences as identified with MALDI-TOF are written on top or next to their corresponding HPLC signal. Characteristics signals at around 8 min and 12 min correspond to buffer component dithiotheritrol (DTT) and cathepsin, respectively. Aliquots were taken after 5 s, 30 s, 2 min, 6 min, 20 min, and 60 min of incubation with cathepsins V and L (both peptides) or K (peptide KVLATVTK).

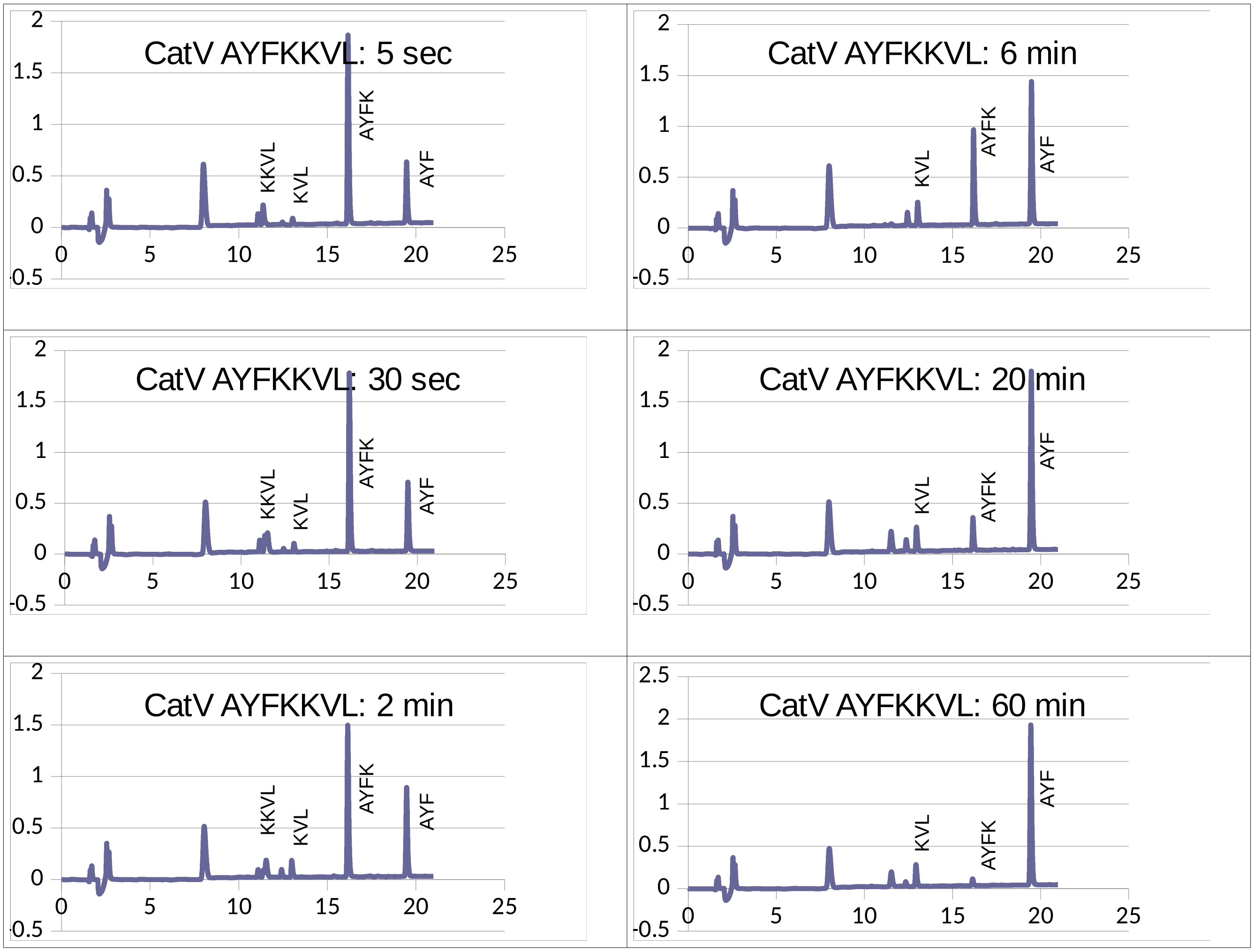

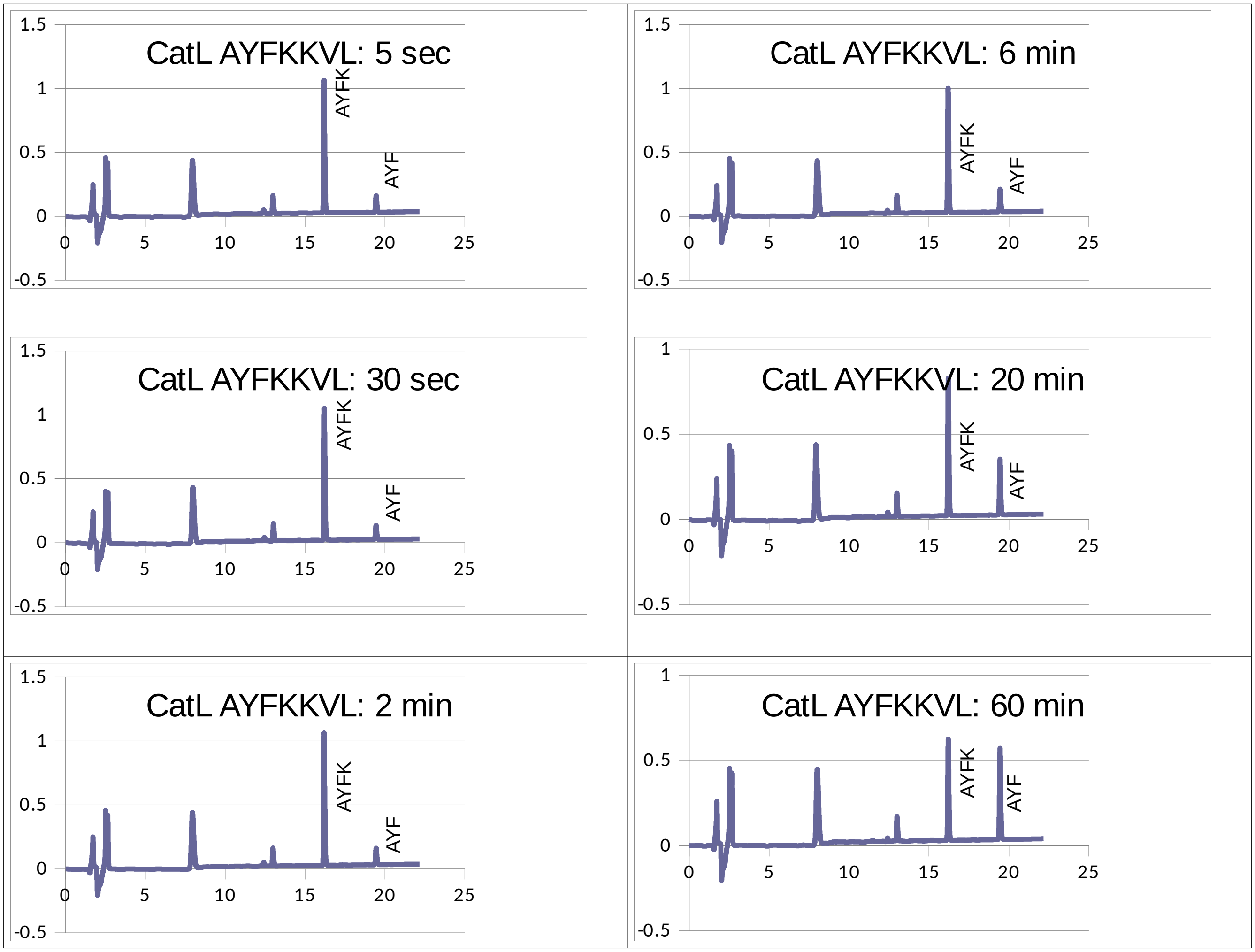

CatL AYFKKVL: 5 sec
AYFK
AYF
CatL AYFKKVL: 6 min
AYFK
AYF
CatL AYFKKVL: 30 sec
AYFK
AYF
CatL AYFKKVL: 20 min
AYFK
AYF
CatL AYFKKVL: 2 min
AYFK
AYF
CatL AYFKKVL: 60 min
AYFK
AYF
1.5
1
0.5
0
-0.5
0
5
10
15
20
25

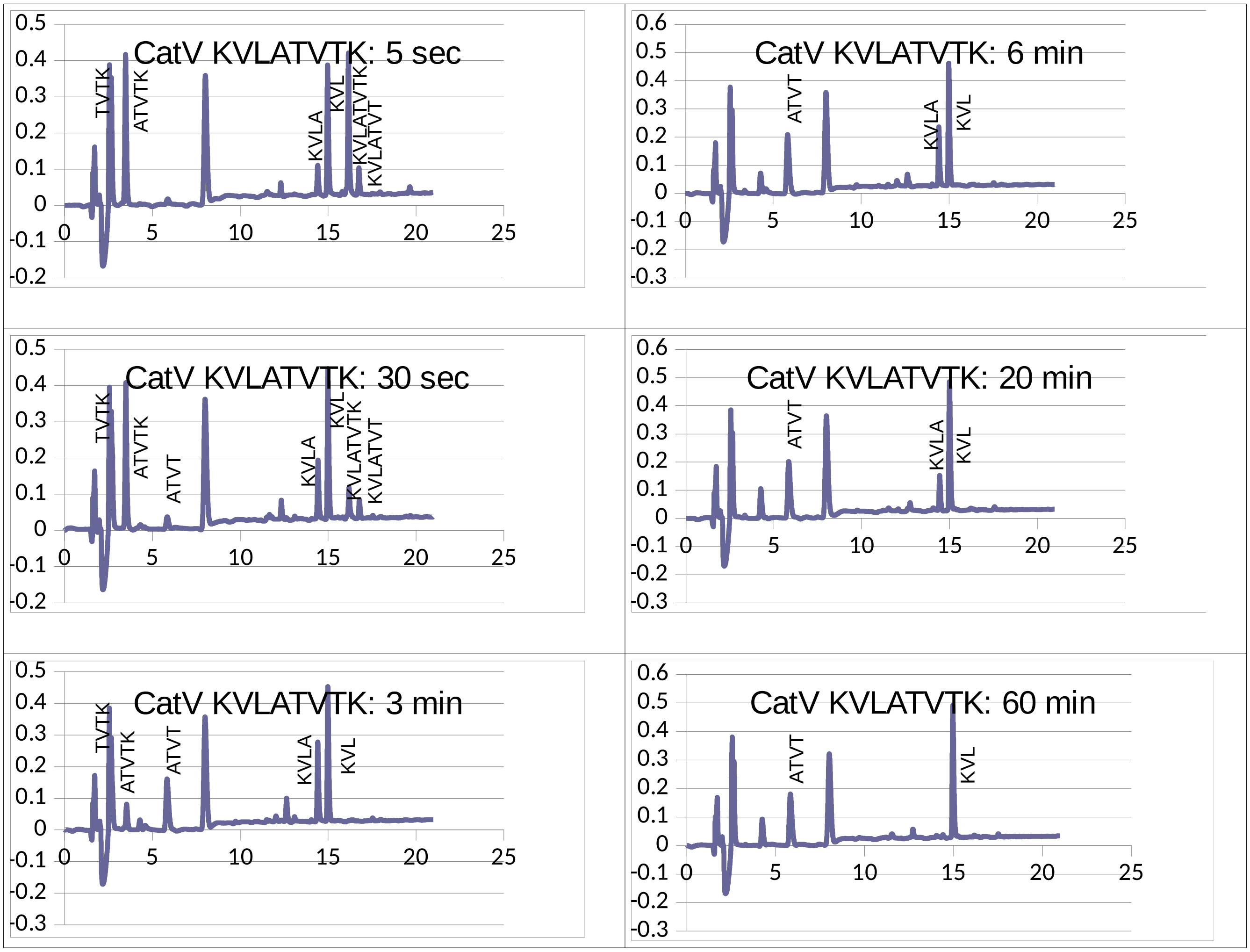

CatV KVLATVTK: 5 sec
TVTK
ATVTK
KVLA
KVL
KVLATVTK
KVLATVT
CatV KVLATVTK: 6 min
ATVT
KVLA
KVL
CatV KVLATVTK: 30 sec
TVTK
ATVTK
ATVT
KVLA
KVL
KVLATVTK
KVLATVT
CatV KVLATVTK: 20 min
ATVT
KVLA
KVL
CatV KVLATVTK: 3 min
TVTK
ATVTK
ATVT
KVLA
KVL
CatV KVLATVTK: 60 min
ATVT
KVL

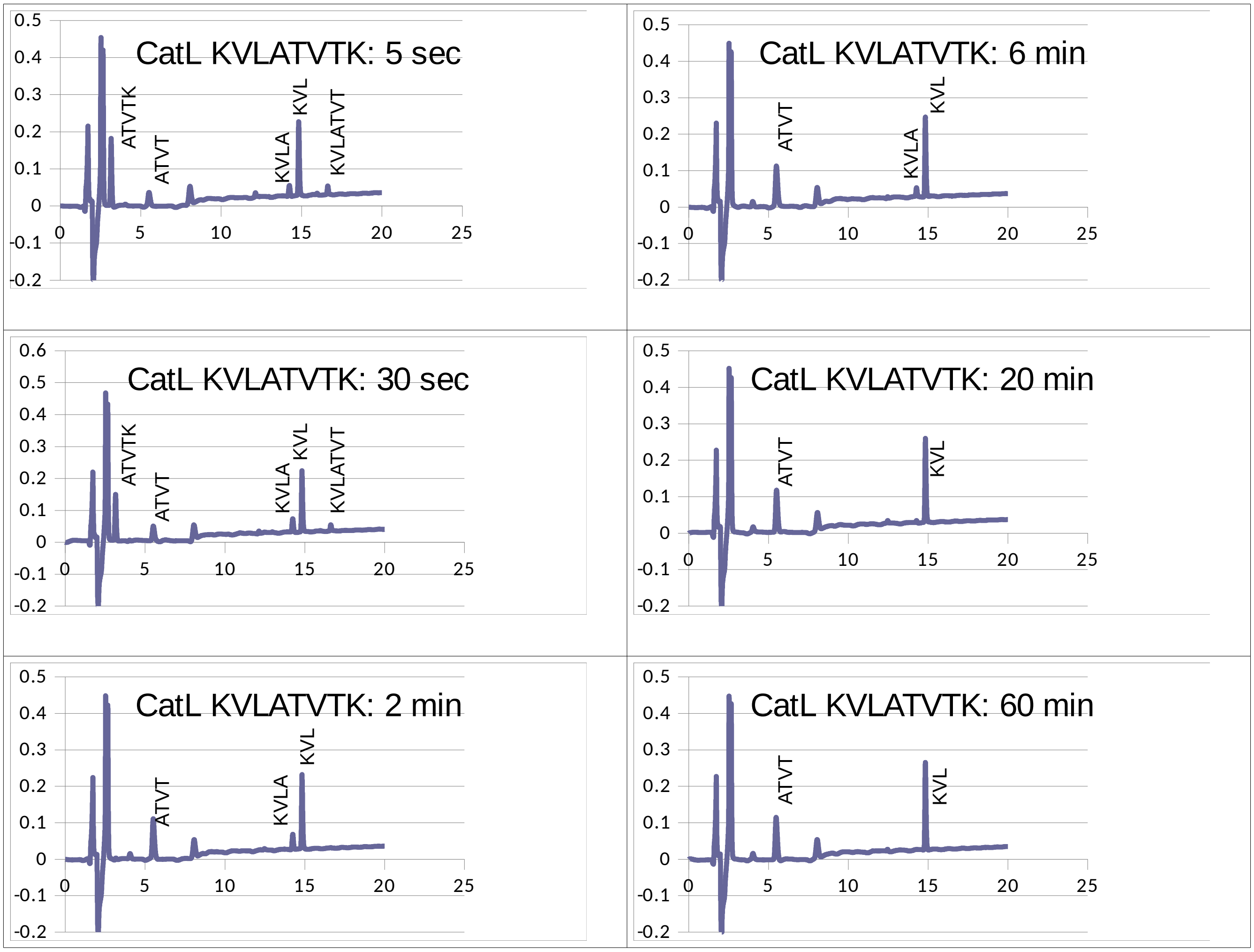

CatL KVLATVTK: 5 sec
ATVTK
ATVT
KVLA
KVL
KVLATVT
CatL KVLATVTK: 6 min
ATVT
KVLA
KVL
CatL KVLATVTK: 30 sec
ATVTK
ATVT
KVLA
KVL
KVLATVT
CatL KVLATVTK: 20 min
ATVT
KVL
CatL KVLATVTK: 2 min
ATVT
KVLA
KVL
CatL KVLATVTK: 60 min
ATVT
KVL

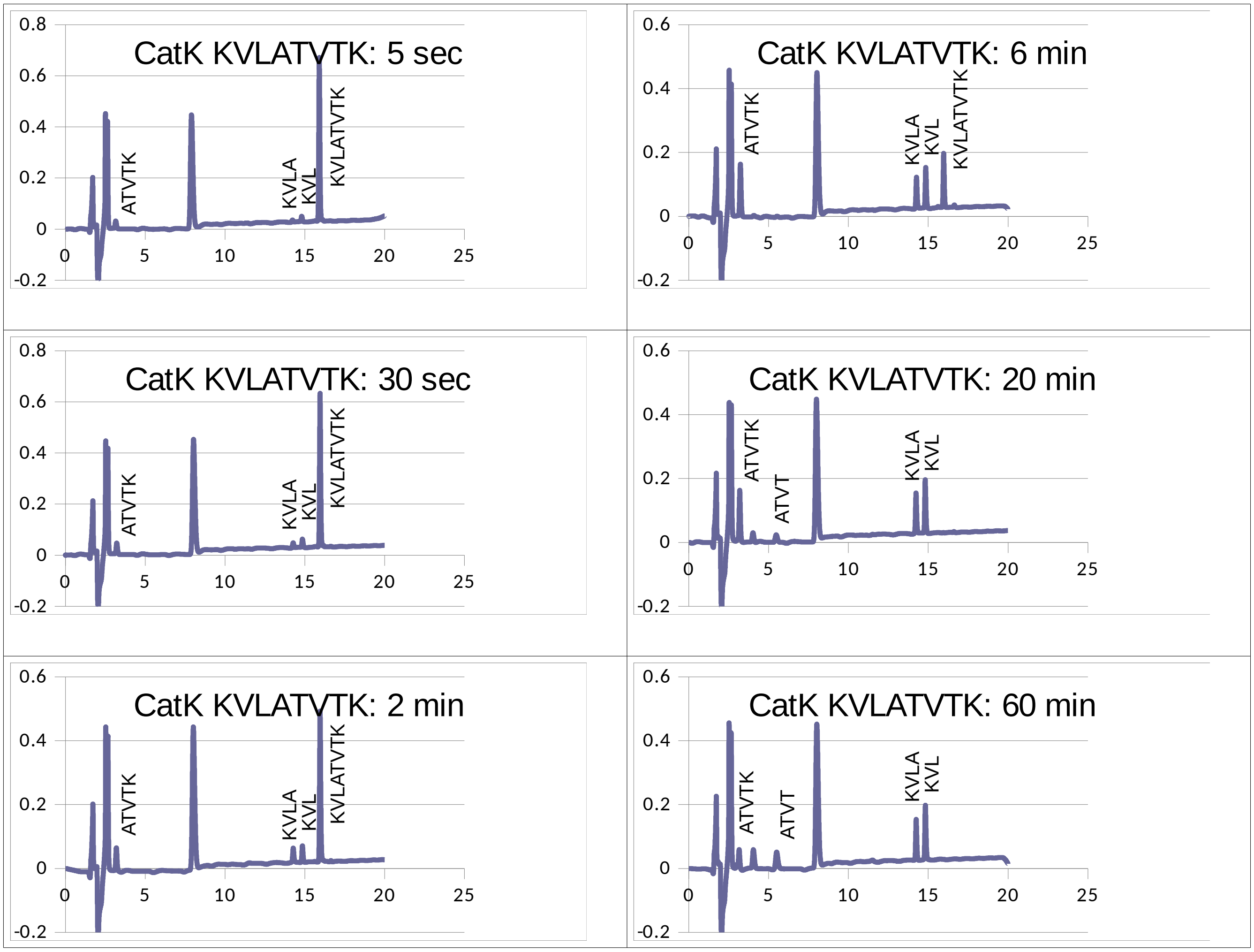
CatK KVLATVTK: 5 sec
ATVTK
KVLA
KVL
KVLATVTK
CatK KVLATVTK: 6 min
ATVTK
KVLA
KVL
KVLATVTK
CatK KVLATVTK: 30 sec
ATVTK
KVLA
KVL
KVLATVTK
CatK KVLATVTK: 20 min
ATVTK
ATVT
KVLA
KVL
CatK KVLATVTK: 2 min
ATVTK
KVLA
KVL
KVLATVTK
CatK KVLATVTK: 60 min
ATVTK
ATVT
KVLA
KVL
