# Supplementary material for: Proteomic data and structure analysis combined reveal interplay of structural rigidity and flexibility on selectivity of cysteine cathepsins
Source: Commun Biol. 2023 Apr 24;6:450. doi: 10.1038/s42003-023-04772-8 (PMC10124925; doi:10.1038/s42003-023-04772-8)
Supplement: Supplementary file 6 — Reporting Summary [file 42003_2023_4772_MOESM6_ESM.pdf]

Corresponding author(s): Gevaert, Turk

Last updated by author(s): 12 April 2023

Reporting Summary

Nature Portfolio wishes to improve the reproducibility of the work that we publish. This form provides structure for consistency and transparency in reporting. For further information on Nature Portfolio policies, see our Editorial Policies and the Editorial Policy Checklist.

Statistics

For all statistical analyses, confirm that the following items are present in the figure legend, table legend, main text, or Methods section.

n/a	Confirmed	
☐	☒	The exact sample size (*n*) for each experimental group/condition, given as a discrete number and unit of measurement
☒	☐	A statement on whether measurements were taken from distinct samples or whether the same sample was measured repeatedly
☒	☐	The statistical test(s) used AND whether they are one- or two-sided<br>*Only common tests should be described solely by name; describe more complex techniques in the Methods section.*
☒	☐	A description of all covariates tested
☒	☐	A description of any assumptions or corrections, such as tests of normality and adjustment for multiple comparisons
☐	☒	A full description of the statistical parameters including central tendency (e.g. means) or other basic estimates (e.g. regression coefficient) AND variation (e.g. standard deviation) or associated estimates of uncertainty (e.g. confidence intervals)
☒	☐	For null hypothesis testing, the test statistic (e.g. *F*, *t*, *r*) with confidence intervals, effect sizes, degrees of freedom and *P* value noted<br>*Give P values as exact values whenever suitable.*
☒	☐	For Bayesian analysis, information on the choice of priors and Markov chain Monte Carlo settings
☒	☐	For hierarchical and complex designs, identification of the appropriate level for tests and full reporting of outcomes
☒	☐	Estimates of effect sizes (e.g. Cohen's *d*, Pearson's *r*), indicating how they were calculated

Our web collection on statistics for biologists contains articles on many of the points above.

Software and code

Policy information about availability of computer code

Data collection

a. Identification of peptides of cathepsins K, V, B, L, S, and F: The recorded MS/MS spectra were searched with MASCOT using the MASCOT Daemon interface (version 2.4.0, www.matrixscience.com) in the Swiss-Prot database (release of November 11, 2011, January 25, 2012 and April 18, 2012). Potential false positive peptide identifications were selected and automatically removed by the Peptizer software application. Neo-N-terminal peptides were then loaded into the TOPPR database, and re-mapped onto the latest version of the Swiss-Prot database (release of January 22, 2014) to generate final lists of total cleavage sites (Supplementary Data 1). All together 29,674 peptides were identified.
b. By using search tools in UniProt database (https://www.uniprot.org/) for 29,674 peptides (originated from 3,167 proteins) PDB codes were gathered and later downloaded from PDB protein data bank by using their program tools (https://www.rcsb.org/). We downloaded structures determined by x-ray (1,592) (in January 2019).
c. MEROPS data sets of cathepsins K, V, B, L, and S substrates (10,443) were downloaded in August 2022 (https://www.ebi.ac.uk/merops/).
d. MEROPS data sets of substrates for the following enzymes peptidyl-Lys metallopeptidase, caspase-3, granzyme B, caspase-7, glutamyl endopeptidase I, cathepsin E, cathepsin D, cathepsin G substrates (12,439) were downloaded in August 2022.
e. 21 structures of complexes cathepsin V - peptides were determined by using XDS Program Package (Version February 2021 https://xds.mr.mpg.de/), PHASER-2.8.3 (CCP4: Supported program https://www.ccp4.ac.uk/html/phaser.html), MAIN program (release in 2021 https://www-bmb.ijs.si/) and RASTER 3D 3.0 (September 2020 http://www.bmsc.washington.edu/raster3d/).
f. Identification of cleaved selected peptides (28) by cathepsins K, L, V: the MS spectra were acquired, processed and calibrating by using FlexControl 3.0 program and FlexAnalysis software (Bruker). All together 150 peptides were identified.

Data analysis

Data sets were processed with SAPS-ESI software platform (Statistical Approach to Peptidyl Substrate-Enzyme Specific Interactions) consisting of codes in GNU fortran 10.2.0 (gcc 10.2.0) and SAS for Windows (SAS 9.4 TS Level 1M7, X64_10PRO platform, licensed to CIPKeBiP, site 70126232), with programs MAIN (released in 2021) and PCSS server (https://modbase.compbio.ucsf.edu/peptide/). The results were post-

processed, analyzed and plotted by using SAS for Windows (SAS 9.4 TS Level 1M7, X64_10PRO platform, licensed to CIPKeBiP, site 70126232), MAIN program (release in 2021 https://www-bmb.ijs.si/), RASTER 3D 3.0 (September 2020 http://www.bmsc.washington.edu/raster3d/), Microsoft Office LTSC Professional Plus 2021: Excel (licensed to IJS) and Microsoft Visio Premium 2010 (licensed to IJS).
Computer codes in SAS and Fortran as part of SAPS-ESI platform are available upon request. MAIN program and RASTER 3D 3.0 are publicly available.

For manuscripts utilizing custom algorithms or software that are central to the research but not yet described in published literature, software must be made available to editors and reviewers. We strongly encourage code deposition in a community repository (e.g. GitHub). See the Nature Portfolio guidelines for submitting code & software for further information.

Data

Policy information about availability of data

All manuscripts must include a data availability statement. This statement should provide the following information, where applicable:

- Accession codes, unique identifiers, or web links for publicly available datasets
- A description of any restrictions on data availability
- For clinical datasets or third party data, please ensure that the statement adheres to our policy

Data sets of K, V, B, L, S, and F cathepsins substrates (excel files), SVM models (zip file with txt files) and results of HPLC analysis (pdf file) are available as Supplementary Data 1 to 4.
The data sets from MEROPS database are publicly available (https://www.ebi.ac.uk/merops/).
Cathepsin V – peptide complexes were deposited to Protein Data Bank (PDB) and were assigned following entries: Cathepsin V – EVCKKKK (7Q8H), Cathepsin V – TRESEDLE (7Q8D), Cathepsin V - GNYKEAKK (7Q8F), Cathepsin V – VPCGTAHE (7Q8L), Cathepsin V – KPKKKTK (7Q8M), Cathepsin V – KKYDAFLA (7Q8N), Cathepsin V – AVAEKQ (7Q8I), Cathepsin V - RLSAKP (protected; 7Q9C), Cathepsin V - RLSAKP (non-protected; 7Q8Q), Cathepsin V - LLKAVAEKQ (7Q9H), Cathepsin V - GAKSAA (non-protected; 7QO2), Cathepsin V - GAKSAA (protected; 7QHJ), Cathepsin V – LLKVAL (co-crystallized; 7Q8K), Cathepsin V - LLKVAL (soaked; 7Q8P), Cathepsin V - VACKSSQP (7QFF), Cathepsin V - AYFKKVL (7QFH), Cathepsin V - ALAASS (7Q8G), Cathepsin V – LLSGKE (7Q8O), Cathepsin V – IILKEK (7Q8J), Cathepsin V - QLRQQE (7QHK), and Cathepsin V - VYEKKP (7QNS).
The mass spectrometry proteomics data have been deposited to the ProteomeXchange Consortium via the PRIDE partner repository with the dataset identifier PXD041185and 10.6019/PXD041185.

Human research participants

Policy information about studies involving human research participants and Sex and Gender in Research.

Reporting on sex and gender	*Use the terms sex (biological attribute) and gender (shaped by social and cultural circumstances) carefully in order to avoid confusing both terms. Indicate if findings apply to only one sex or gender; describe whether sex and gender were considered in study design whether sex and/or gender was determined based on self-reporting or assigned and methods used. Provide in the source data disaggregated sex and gender data where this information has been collected, and consent has been obtained for sharing of individual-level data; provide overall numbers in this Reporting Summary. Please state if this information has not been collected. Report sex- and gender-based analyses where performed, justify reasons for lack of sex- and gender-based analysis.*
Population characteristics	*Describe the covariate-relevant population characteristics of the human research participants (e.g. age, genotypic information, past and current diagnosis and treatment categories). If you filled out the behavioural & social sciences study design questions and have nothing to add here, write "See above."*
Recruitment	*Describe how participants were recruited. Outline any potential self-selection bias or other biases that may be present and how these are likely to impact results.*
Ethics oversight	*Identify the organization(s) that approved the study protocol.*

Note that full information on the approval of the study protocol must also be provided in the manuscript.

Field-specific reporting

Please select the one below that is the best fit for your research. If you are not sure, read the appropriate sections before making your selection.

☒ Life sciences ☐ Behavioural & social sciences ☐ Ecological, evolutionary & environmental sciences

For a reference copy of the document with all sections, see nature.com/documents/nr-reporting-summary-flat.pdf

Life sciences study design

All studies must disclose on these points even when the disclosure is negative.

Sample size	The existing datasets of substrates of cathepsins K (9,583), V (4,415), B (4,254), L (4,117), S (3,805), and F (3,500) were used for analysis. For modelling by using Support Vector Machine algorithm the training set consisted of "positive" peptides of cathepsins K (2,253), V (2,081), B (4,006), L (1,938), S (1,792), and F (3,277) and "negative" peptides 3,526 (K), 4,508 (V), 4,508 (B), 3,948 (L), 3,526 (S), and 4,508 (F) separately for individual cathepsins. The total number of peptides of cathepsins K, V, B, L, S, and F for testing of SVM models were 10,466, 2,002, 1,326,

1,978, 1,773, and 993, respectively.
For validation of heterogeneous and homogeneous positions the substrates of K (2,190), V (1,649), B (581), L (2,911), and S (3,112) cathepsins downloaded from MEROPS (https://www.ebi.ac.uk/merops/) were used. Additionally, heterogeneous and homogeneous positions were tested for substrates of the following enzymes: peptidyl-Lys metallopeptidase (2,104), caspase-3 (680), granzyme B (1,900), caspase-7 (499), glutamyl endopeptidase I (4,324), cathepsin E (1,586), cathepsin D (899), and cathepsin G (447).
35 selected peptides of cathepsin V were used for determination of complexes cathepsin V - peptide. Identification of cleaved selected peptides (28) by cathepsins K, L, V resulted in 150 peptides.

Data exclusions

In the case of clusters, 4 peptides out of 4,254 of cathepsin B and 4 peptides out of 4,117 for cathepsin L were excluded because they didn't have all heterogeneous positions. An example of excluded peptide is peptide M P - V K K K R K S P G V A A A V A.
In the case of peptides selected for training SVM models, some peptides were excluded due to mismatches between peptide sequences read from the input file and what was stored in modbase of PCSS server. From training sets for SVM models 80 proteins were excluded in total because they contained some of 35 peptides selected for structural studies (21) and additional cleavages of native cathepsins K, V, and L (sample of 150 peptides). S protein of SARS-CoV-2 in total was not included into training sets of any cathepsin.

Replication

Processing of data sets and their analysis can be replicated.

Randomization

Randomization was not relevant.

Blinding

Blinding was not relevant.

Reporting for specific materials, systems and methods

We require information from authors about some types of materials, experimental systems and methods used in many studies. Here, indicate whether each material, system or method listed is relevant to your study. If you are not sure if a list item applies to your research, read the appropriate section before selecting a response.

Materials & experimental systems

n/a	Involved in the study	
☒	☐	Antibodies
☒	☐	Eukaryotic cell lines
☒	☐	Palaeontology and archaeology
☒	☐	Animals and other organisms
☒	☐	Clinical data
☒	☐	Dual use research of concern

Methods

n/a	Involved in the study	
☒	☐	ChIP-seq
☒	☐	Flow cytometry
☒	☐	MRI-based neuroimaging
